# Investigation of Genetic Causes in Patients with Congenital Heart Disease in Qatar: Findings from the Sidra Cardiac Registry

**DOI:** 10.3390/genes13081369

**Published:** 2022-07-30

**Authors:** Sarah Okashah, Dhanya Vasudeva, Aya El Jerbi, Houssein Khodjet-El-khil, Mashael Al-Shafai, Najeeb Syed, Marios Kambouris, Sharda Udassi, Luis R. Saraiva, Hesham Al-Saloos, Jai Udassi, Kholoud N. Al-Shafai

**Affiliations:** 1 Department of Biomedical Sciences, College of Health Sciences, QU Health, Qatar University, Doha P.O. Box 2713, Qatar; so1404563@qu.edu.qa (S.O.); helkhil@qu.edu.qa (H.K.-E.-k.); malshafai@qu.edu.qa (M.A.-S.); 2 Translational Medicine Division, Sidra Research, Sidra Medicine, Doha P.O. Box 26999, Qatar; dvasudeva@sidra.org (D.V.); nsyed@sidra.org (N.S.); lsaraiva@sidra.org (L.R.S.); 3 Cardiology Division, Heart Center Department, Sidra Medicine, Doha P.O. Box 26999, Qatar; aeljerbi@sidra.org (A.E.J.); halsaloos@sidra.org (H.A.-S.); jai.udassi1@hsc.wvu.edu (J.U.); 4 Pathology & Laboratory Medicine Department, Genetics Division, Sidra Medicine, Doha P.O. Box 26999, Qatar; mkambouris@sidra.org; 5 Division of General Academic Pediatrics, Sidra Medicine, Doha P.O. Box 26999, Qatar; sharda.udassi@hsc.wvu.edu; 6 Pediatric Hospital Medicine Division, WVU Medicine, West Virginia, WV 26506, USA; 7 College of Health and Life Sciences, Hamad Bin Khalifa University, Doha P.O. Box 34110, Qatar; 8 Monell Chemical Senses Center, 3500 Market Street, Philadelphia, PA 19104, USA; 9 Children’s Heart Center, WVU Children’s Hospital, WVU School of Medicine, West Virginia University, West Virginia, WV 26506, USA

**Keywords:** congenital heart defect, Qatar, genetic investigation, whole exome sequencing, single-nucleotide variant, chromosomal abnormalities

## Abstract

Congenital heart disease (CHD) is one of the most common forms of birth defects worldwide, with a prevalence of 1–2% in newborns. CHD is a multifactorial disease partially caused by genetic defects, including chromosomal abnormalities and single gene mutations. Here, we describe the Sidra Cardiac Registry, which includes 52 families and a total of 178 individuals, and investigate the genetic etiology of CHD in Qatar. We reviewed the results of genetic tests conducted in patients as part of their clinical evaluation, including chromosomal testing. We also performed whole exome sequencing (WES) to identify potential causative variants. Sixteen patients with CHD had chromosomal abnormalities that explained their complex CHD phenotype, including six patients with trisomy 21. Moreover, using exome analysis, we identified potential CHD variants in 24 patients, revealing 65 potential variants in 56 genes. Four variants were classified as pathogenic/likely pathogenic based on the American College of Medical Genetics and Genomics and the Association for Molecular Pathology (ACMG/AMP) classification; these variants were detected in four patients. This study sheds light on several potential genetic variants contributing to the development of CHD. Additional functional studies are needed to better understand the role of the identified variants in the pathogenesis of CHD.

## 1. Introduction

Congenital heart disease (CHD) is one of the most common birth defects worldwide, with a general prevalence of 1–2% in newborns [1]. The prevalence of specific manifestations of CHD varies significantly in different populations, and their genetic etiology is incredibly complex [2]. For example, sporadic cases account for 5–10% of all cases of CHD with a genetic component, and only a small fraction of CHD cases is described as familial [2]. Moreover, isolated cases of CHD are more likely to be caused by mutations in single genes compared to chromosomal aberrations. To date, ~400 genes have been shown to be associated with this disease, but ~60% of CHD cases remain unexplained.

Epidemiological studies show a higher incidence of CHD in Arab countries due to high consanguinity and endogamy [3,4]. For instance, it was reported that the incidence of CHD in the province of Al Qassim in Saudi Arabia doubled compared to that in the United States of America (USA) [5]. The annual incidence of CHD among live births is estimated to be ~9–15/1000 in Qatar, ~12/1000 in Jordan, ~11/1000 in Lebanon, ~7/1000 in Oman, and ~10/1000 in Gaza city in Palestine [6,7,8,9,10]. In Egypt, consanguinity was reported in 44% of CHD cases, whereas it was around 30.5% in Tunisia [11,12]. Moreover, in 2015, 65% of fetuses affected by CHD in the United Arab Emirates came from consanguineous families [4].

Here, we established the Sidra Cardiac Registry in Qatar to collect research data and samples from patients with CHD, cardiomyopathies, and other cardiac phenotypes, as well as from their relatives. We also studied the genetic architecture of 52 patients with CHD, identifying the potential variants and genes that may explain their clinical phenotype. Our findings may help shed light on the possible increased prevalence of hereditary forms of CHD in Arabs and the importance of genetic research among the Arab and Middle Eastern populations, which remain insufficiently studied.

## 2. Materials and Methods

### 2.1. Ethical Approval

Ethical approval was obtained from the Institutional Review Boards of Sidra Medicine (IRB #1500769) and Qatar University (IRB #1666-E/22) in accordance with the Declaration of Helsinki. Informed consent/assent was obtained from all study participants. The criteria for inclusion in the Sidra Cardiac Registry were patients from Sidra Medicine with cardiac phenotypes (e.g., CHD, cardiomyopathy, channelopathy), first- and second-degree relatives of patients with cardiac phenotypes, Qatari and non-Qatari participants, as well as children and adults. Eligible participants who could not provide informed consent/assent due to medical reasons or language barriers were excluded.

### 2.2. Study Participants and Data Collection

The study included 52 patients with CHD enrolled in the Sidra Cardiac Registry at Sidra Medicine in Qatar, in addition to 126 family members (2 affected, 124 unaffected). The classification of patients’ congenital malformations corresponded to the nomenclature of the International Classification of Diseases, Tenth Revision (ICD-10) [13]. Demographic, clinical, and family history data were collected from the study participants along with 1–10 mL of whole blood, which was required for genetic investigation. Family pedigrees were generated using HaploPainter (http://haplopainter.sourceforge.net/) (accessed on 1 January 2022). Data about cytogenetic tests, such as karyotyping, chromosomal microarray, Fluorescence In Situ Hybridization (FISH), performed as part of a patient’s evaluation, were obtained along with the pathogenicity interpretation report that was provided by the laboratory where the test was performed.

### 2.3. Whole Exome Sequencing (WES)

Patients who did not undergo chromosomal testing (n = 25) and patients who had a negative result for chromosomal abnormalities (n = 11) were included in the whole exome sequencing (WES) analysis together with their family members who were enrolled in the study (n = 77). Genomic DNA was extracted from blood, and WES libraries were prepared using the SureSelect kit (Agilent, Santa Clara, CA, USA), followed by sequencing using a HiSeq 4000 (Illumina, San Diego, CA, USA) at Integrated Genomic Services, Sidra Medicine.

### 2.4. Sequence Quality, Alignment, and Variant Calling

The WES pipeline involved the following: multiplexed raw sequencing files (.bcl files) were converted into separate FastQ files, and the quality of the sequencing reads was assessed using FASTQC [14]. Adaptor sequences were trimmed using trimadap (https://github.com/lh3/trimadap) (accessed on 1 January 2022) and high-quality reads were mapped to the National Center for Biotechnology Information (NCBI) human reference genome CRGh37/hg19 (National Institute of Health, New York, USA) using Burrows-Wheeler Aligner (BWA) v0.7.8 (Broad Institute, Cambridge, Massachusetts, USA), in particular, the BWA-MEM algorithm (arXiv:1303.3997[q-bio-GN]). Duplicate reads were marked after alignment using SAMBLASTER [15], and BAM files were sorted using SAMtools [16]. These steps were accomplished using the BWA-kit package. Picard and Mosdepth tools were used to perform the alignment quality control. After alignment, the reads were recalibrated, and variants were called using the Genome Analysis Toolkit (GATK) v4.1 [17]. The Exome-Agilent-V6.bed file was fed into the variant calling step to limit the variant calling to exome regions. The individual g.VCF files were joined to generate a single merged VCF file for all samples. We also ran peddy on the merged VCF file to ensure the pedigree assignment was correct before performing segregation analysis. The joined VCF file was split, decomposed, and normalized using VT software v0.57 (University of Michigan, Michigan, USA) [18]. The joint normalized VCF file was annotated using SnpEff v4.3 (http://pcingola.github.io/SnpEff/) (accessed on 1 January 2022) [19], classifying variants into high, moderate, and low impact, based on their predicted pathogenic effect on the gene [19]. Variants were also annotated with the following:

(a) Allele frequencies, obtained from the Genome Aggregation Database (gnomAD) (https://gnomad.broadinstitute.org/) (accessed on 1 January 2022), Qatar 1000 Genomes [20,21], and Greater Middle East (GME) Variome Project (http://igm.ucsd.edu/gme/) (accessed on 1 January 2022) [22].
(b) Variant pathogenicity prediction scores, including Sorting Intolerant From Tolerant (SIFT), Polymorphism Phenotyping (PolyPhen), and Combined Annotation Dependent Depletion (CADD).
(c) Variant phenotypic and abnormality-related information using the available databases, mainly the Human Gene Mutation Database (HGMD^®^), ClinVar (https://www.ncbi.nlm.nih.gov/clinvar/) (accessed on 1 January 2022) and Genome-Wide Association Study (GWAS) Catalog.
(d) Information on the phenotypic abnormalities of the captured genes, obtained from Human Phenotype Ontology (HPO|) (https://hpo.jax.org/app/) (accessed on 1 January 2022) [23].

### 2.5. Single-Nucleotide Variant (SNV) Segregation Analysis and Filtration

**Figure 1:**
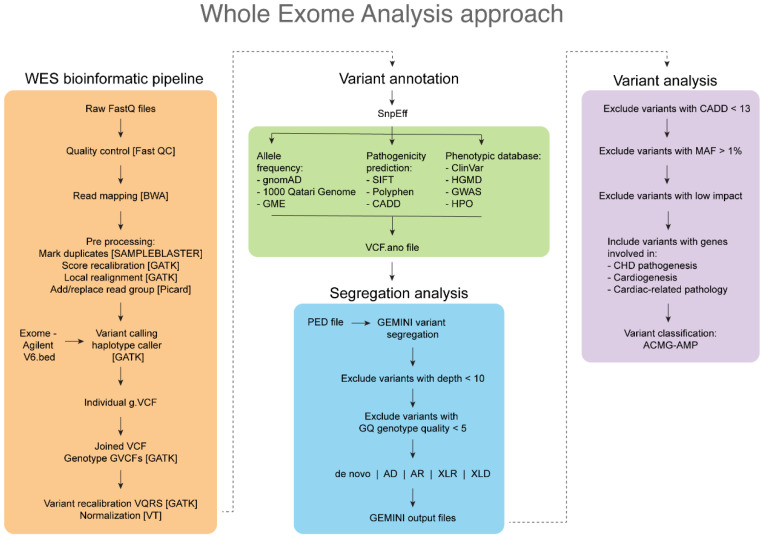
A flowchart of the whole exome sequencing analysis (WES) approach performed in this study.

Familial segregation analysis was performed using genome mining (Gemini) v0.30.2 (https://gemini.readthedocs.io/en/latest/) (accessed on 1 January 2022). The final annotated VCF file was imported into the Gemini database along with the pedigree (PED) file using vcf2db utils. Gemini provided various segregation patterns (i.e., de novo, autosomal dominant, autosomal recessive, compound heterozygous, X-linked recessive, and X-linked dominant), and a custom Python script was used to write the Gemini output data to Excel sheets. Then, the obtained variants were filtered by removing those with a CADD value of <13, minor allele frequency (MAF) of >1% in the control databases (gnomAD, Qatar 1000 genome, GME), and low impact according to the SnpEff annotation [24] (Figure 1).

Prioritization of the genes was conducted by searching PubMed (https://pubmed.ncbi.nlm.nih.gov/), Google (http://google.com), and Google Scholar (https://scholar.google.com/) (accessed on 1 February to March 2022). For search queries, we combined the gene code AND “cardiac” OR “heart” OR “cardiac defect” OR “congenital cardiac defect” OR “malformation” OR “defect”. We extracted data on the molecular role of the gene in cardiogenesis or cardiac pathology, in vitro or in vivo experiments, as well as case reports of an association between the gene or its variant and CHD.

The American College of Medical Genetics and Genomics and the Association for Molecular Pathology (ACMG/AMP) guidelines [25] were used to classify variants into “benign”, “likely benign”, “uncertain significance”, “likely pathogenic”, and “pathogenic” using the InterVar online tool (https://wintervar.wglab.org/) (accessed on 1 February to 1 March 2022). The novelty of the variant was established if it had not been reported in the literature or publicly available databases.

### 2.6. Statistical Analysis

Fisher’s exact tests were used to examine the association between obtaining a positive result in chromosomal testing or WES analysis and the demographic data of patients. Correlations were considered significant if the *p*-value was <0.05 across the demographic data (i.e., survival, ethnicity, gender, family history of CHD, family history of congenital disease, and consanguinity). Statistical tests were performed separately for patients who underwent chromosomal testing (n = 27) and for patients included in the WES analysis (n = 36). All statistical analyses were performed using IBM-SPSS v.28.

## 3. Results

### 3.1. Demographic and Clinical Characteristics

**Figure 2:**
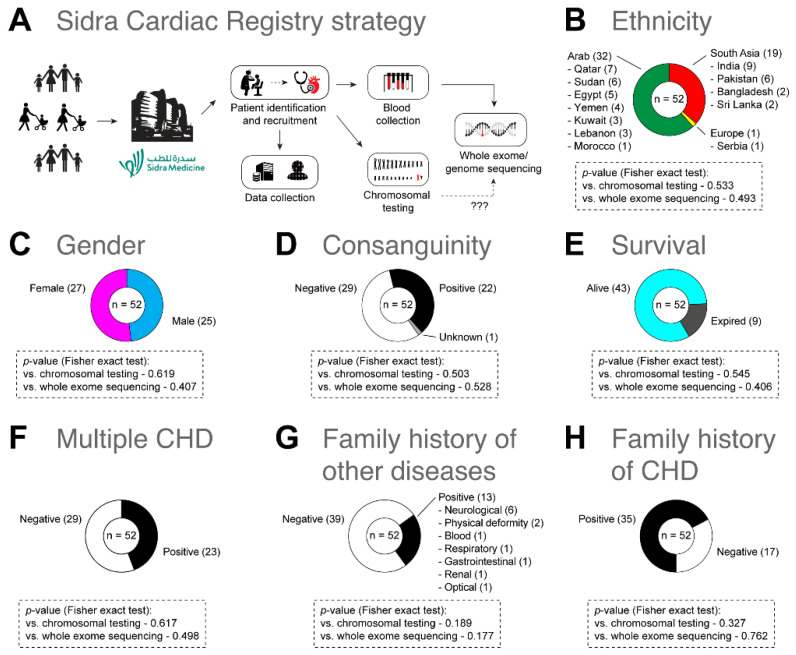
(**A**) Sidra Cardiac Registry strategy. (**B**–**H**) A summary of demographic and clinical characteristics of patients with CHD (n = 52). Fisher’s exact tests were used to examine the association between obtaining a positive result in chromosomal testing or whole exome sequencing (WES) analysis and the demographic data of patients.

The Sidra Cardiac Registry included 52 patients suffering from CHD, as well as a total of 126 family members. While 43 out of 52 families contained at least a trio (i.e., a proband and both parents), the remaining 9 had a proband with only a mother or father. Of all patients, 61.50% (n = 32) were Arab from Qatar, Syria, Yemen, Tunisia, Morocco, Egypt, Sudan, Lebanon, and Kuwait, followed by 36.50% (n = 19) South Asian from India, Pakistan, Sri Lanka, and Bangladesh, and one European from Serbia. Of our patients, 48% (n = 25) were females and 52% (n = 27) were males. All 52 patients belonged to the pediatric population with the mean age of 1 year and 2 months (SD = ±3.6). The maximum age of patients was 18 years, while the minimum was 2 days. The consanguinity rate was 2.30% (n = 22). Of 52 patients with CHD, 44.20% (n = 23) had several cardiac defects. In addition, 32.70% (n = 17) had a positive family history of CHD, and 25% (n = 13) had a positive family history of non-cardiac congenital diseases, including neurological (n = 6), blood (n = 1), gastrointestinal (n = 2), respiratory (n = 1), limb deformity (n = 1), renal (n = 1), and ocular (n = 1) diseases. Approximately 82.7% (n = 43) of patients were alive during the study; however, 17.30% (n = 9) expired after enrollment due to cardiac complications or systemic failure (Figure 2). The patients ‘pedigree is provided in the Supplementary Material.

**Figure 3:**
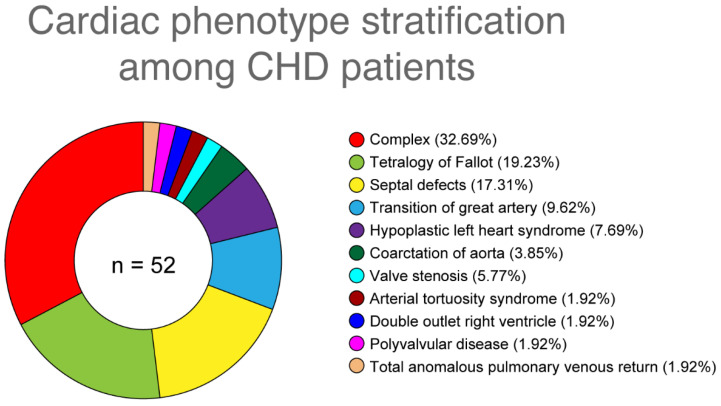
Cardiac phenotypes reported in patients with CHD (n = 52).

Eleven types of CHD phenotypes were identified in 52 patients, distributed as follows: 19.23% (n = 10) of cases presented with tetralogy of Fallot (TOF), 17.30% (n = 9) had septal defects (arterial septal defect, ventricular septal defect), 7.69% (n = 4) had hypoplastic left heart syndrome (HLHS), 9.61% (n = 5) had transposition of the great arteries (TGA), 1.92% (n = 1) had valve stenosis (pulmonary valve stenosis), 3.84% (n = 2) had coarctation of the aorta (COA), 1.92% (n = 1) had arterial tortuosity syndrome (ATS), 1.92% (n = 1) had double outlet right ventricle (DORV), 1.92% (n = 1) had polyvalvular disease, 1.92% (n = 1) had total anomalous pulmonary venous return (TAPVR), and 32.69% (n = 17) had a combination of several CHD types (Figure 3).

### 3.2. Clinical Cytogenetic Testing

**Figure 4:**
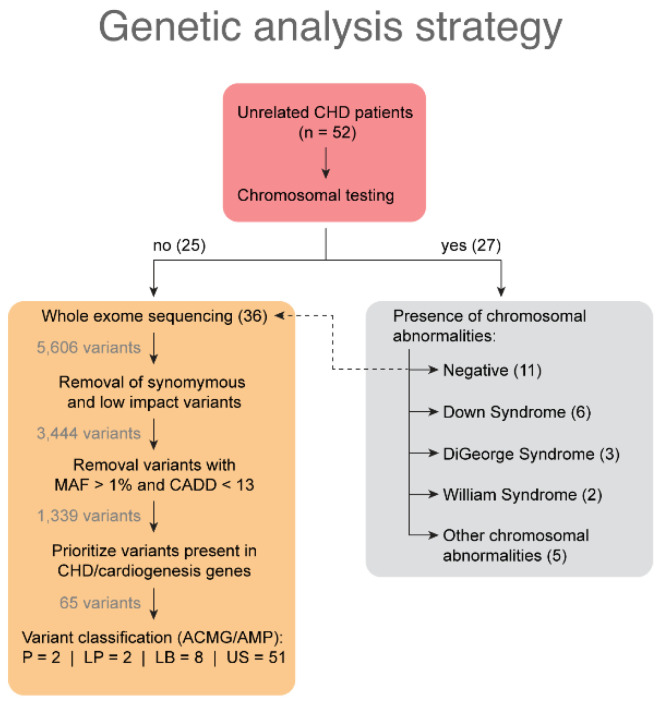
A flowchart illustrating genetic investigations conducted in patients with CHD and the outcomes obtained from chromosomal testing and whole exome sequencing analysis. LB: likely benign; LP: likely pathogenic; P: pathogenic; US: uncertain significance. wo variants could not be classified due to limited data availability.

As part of the clinical evaluation, 27 out of 52 patients (52%) with CHD underwent cytogenetic testing for the presence of chromosomal abnormalities, and 16 out of 27 patients (59.2%) had positive results for chromosomal abnormalities (Figure 4). No significant relationship was found between the demographic characteristics studied in 27 patients with CHD and chromosomal abnormalities (*p* > 0.05).

Six patients had trisomy 21 (Down Syndrome), three had a 22q11.2 deletion (DiGeorge Syndrome), two had a 7q11.23 deletion (William Syndrome), and five had other chromosomal abnormalities (Table 1, Table S1). Additional chromosomal abnormalities were a 9q34.3 deletion (Adam Oliver Syndrome), mosaic trisomy 14, chromosome 6 deletion, 16p11.2 deletion, and 8p11.21 duplication. In addition, two patients (cardio-32.A and cardio-37.A) presented with two different chromosomal aberrations: one patient had a deletion of 10q26.3 and 7q11.23, and the other had a 22q11.2 deletion with mosaic trisomy 8 (Table S1). In all patients, extracardiac phenotypes were identified along with the cardiac phenotype, as shown in Table S1. The clinical laboratory provided interpretation of all chromosomal aberrations, with the exception of cardio-3.A (chromosome 6 deletion) and cardio-45.A (8p11.21 duplication), where no interpretation was provided (Table S1).

Parental testing was performed in seven patients with positive results for chromosomal abnormalities. Three out of seven patients had negative results for any aberrations in both parents (Table S1). In three cases, the mother had normal chromosomal testing results, while the father either had no records, was not enrolled, or had inconclusive results. In one case, there was the same copy number variation (CNV) (16p11.2 deletion) in the father (cardio-56.C) as in the proband (cardio-9.A), and the aberration was classified as likely benign (Table S1). All patients with positive results for chromosomal abnormalities (n = 16) had syndromic CHD with extracardiac manifestations, and 62.50% (n = 10) of the 16 patients manifested with septal defects.

### 3.3. Variants Identified by Whole Exome Sequencing Analysis

Thirty-six patients with CHD, along with 77 family members, were included in the WES analysis, conducted to identify potential gene variants that could cause the disease (Figure 4). Of the 36 families, 28 were trios and included a proband and both parents; three included a proband, both parents, and at least one sibling; four included a proband and only one parent; and one included a proband, one parent, and proband siblings. Across 24 patients, the mean depth of coverage for all genes was 74.31X (SD = ±15.4).

Initially, 5606 variants were detected, while only 65 remained after we applied a series of filtrations (Figure 4). These 65 variants were detected in 56 genes associated with CHD pathogenesis, cardiogenesis, or cardiac pathology; they were present in 24 out of 36 patients. In the remaining 12 patients with CHD, no variants within the prioritized genes were detected.

Based on the ACMG/AMP guidelines, 51 variants (78.46%) were classified as uncertain significant (US), eight (12.30%) were likely benign, two (3.07%) were pathogenic, two (3.07%) were likely pathogenic, and two (3.07%) variants (c.1437-6delT and c.4154-7dupT) could not be classified due to limited data availability (Figure S1). Of the 65 variants, 23 were novel variants that had not been previously identified or reported; they were located in 21 different genes (Table S2).

Our segregation analysis using Gemini showed that 12 (18.46%) variants followed an autosomal recessive inheritance pattern, 2 (3.07%) were autosomal dominant, 20 (30.76%) were compound heterozygous, 2 (3.07%) were X-linked recessive, and 1 (1.53%) showed a double-inheritance autosomal dominant and compound heterozygous pattern. One variant (1.53%) followed uniparental disomy (UPD), eighteen (27.69%) were de novo, and nine (13.84%) could not be attributed to a certain inheritance pattern, as the parents were not enrolled in the study (Figure S1).

Of the 65 variants, 46 were found to be inherited: 16 variants (autosomal dominant, compound heterozygous, or X-linked recessive) were inherited from the mother, 9 were inherited from the father (autosomal dominant or compound heterozygous), and 4 were inherited from both parents (autosomal recessive). In 17 cases, the origin of the variant could not be accurately determined, as one of the parents was not enrolled in the study. Therefore, the origin of the variant in those 17 cases could have been either autosomal dominant or compound heterozygous (Table S4). We identified four pathogenic or likely pathogenic variants in four patients. A patient from Yemen (cardio-27.A), who presented with syndromic CHD (TOF, long QT interval, and hearing loss), had a pathogenic de novo variant c.6292C>T in the CHD7 gene. A patient from Sri Lanka (cardio-12.A), who had multiple cardiac defects, including COA, VSD, and PDA (Table 2), was found to have a pathogenic de novo variant c.2883-1G>T in the ROBO1 gene. In addition, a patient from Pakistan (cardio-15.A) with Shone’s complex had a likely pathogenic variant c.817+1G>C in the SMAD6 gene, and a patient from Yemen (cardio-15.A) with ATS had a possible pathogenic variant c.243C>G in the SLC2A10 gene (Table 2 and Table S4).

We also identified eight likely benign variants in 20.8% (5/24) of patients, which were inherited from either one (n = 3) or both parents (n = 5) (Table S4). Compound heterozygous variants, such as c.98390A>G in TTN, were detected in a patient from Qatar (cardio-2.A) with multiple cardiac defects and an extracardiac phenotype. Other examples were c.3401G>T in IFT172, seen in a patient from Sudan (cardio-36.A) with TOF, and c.2867G>C in COL5A2, seen in a patient with HLHS from Sudan (cardio-31.A). Moreover, we identified two patients, cardio-15.A from Pakistan and cardio-18.A from Qatar, who were diagnosed with multiple cardiac defects. Those patients had homozygous likely benign variants such as p.Val1457Ile in CCDC141 and c.6035C>T and c.1751A>C in CMYAS, respectively. HLHS was present in two patients from Sudan (cardio-31.A and cardio-45.A), with likely benign variants. Three variants were detected in cardio-31.A, including two homozygous variants in DNAAF3 (c.1606G>A and c.131G>T) and one variant c.2867G>C in COL5A2. 

Moreover, we identified 51 different variants classified as US. Most of these variants had been reported previously (n = 30), while others were novel (n = 21) (Table S4). Among the novel US variants, 8 were de novo: c.*16G>A in CCNC, c.49G>C in GGN, c.2054C>T in NUP133, c.123C>G in FOXP1, c.584G>A in LRRC14B, c.343G>A in HIST1H1E, c.2957G>T in ZFPM2, and c.1817A>C in FLT1. Other novel US variants were inherited from the mother in an X-lined recessive pattern: c.10570T>A in HUWE1 and c.463C>T in TBC1D25, detected in patients cardio-12.A and cardio-23.A (Table S4). Other novel US variants were passed on to a patient from only one parent in an autosomal dominant or compound heterozygous inheritance pattern: c.848G>C in CRELD1, c.2204T>A in ADAMTS8, and c.66A>T in SLC24A4 (Table S4). Two US homozygous variants, c.43A>T in BUB1 and c.926A>G in ITGAL, followed an autosomal recessive inheritance pattern; they were detected in two patients from Qatar (cardio-18.A and cardio-38.A) with multiple cardiac defects (Table S4). The presence of 30 US variants identified here had been previously reported by other studies or identified in either control or clinical databases. Nine US variants had been reported among patients with other diseases, for example c.5695G>A in DYNC2H1 (Table S3). The remaining 25 US variants have been reported in control databases at a low frequency but have not been found in the published literature, for example c.5437G>A in PRR12 (Table S3).

## 4. Discussion

In this study, we established the Sidra Cardiac Registry in Qatar and investigated the genetic etiology of 52 patients with CHD and their 126 relatives. As for chromosomal testing, out of 52 patients, 30.7% (n = 16) had confirmed chromosomal abnormalities. In previous studies, it was estimated that 11–13% of CHD cases are associated with chromosomal abnormalities [26,27,28]. Our prevalence rates appear to be higher than the reported prevalence; however, our results may not be representative of the general population due to the small sample size. In 6 of the 16 positive cases (37%), trisomy 21 was detected, with septal defects being the most prominent cardiac finding. These results support former studies that have identified septal defects as the most common form of cardiac abnormality in patients with trisomy 21 [29,30]. Other chromosomal aberrations encountered in our sample were William syndrome and DiGeorge syndrome, both of which have a strong association with CHD [31,32]. Two patients with 22q11.2 deletion had conotruncal heart defects (TOF) along with valve stenosis; however, in one of these cases (cardio-37.A), another aberration was encountered, which was a mosaic nondisjunction in chromosome 8. Previous studies have reported conotruncal defects, such as TOF and DORV, often present in patients with 22q11.2 deletion.

Although discussion of the cardiac phenotype of mosaic trisomy 8 is limited due to a paucity of published data, cardiovascular [33], laterality [34], and septal [35] defects have been reported in several cases. Mosaic trisomy 14 is another chromosomal aberration that was observed in a patient with syndromic CHD (cardio-28.A); it is typically associated with cardiac defects [36], such as patent ductus arteriosus [37], and TOF [38,39]. However, CHD does not seem to always co-occur with trisomy 14. In addition, we encountered four cases with CNVs, such as a 9q34.3 deletion in cardio-9.A, who had multiple cardiac defects along with extracardiac manifestations. The deleted region houses a group of genes, such as *NOTCH1,* associated with Adams–Oliver syndrome. This condition is often accompanied by cardiac malformations, including septal defects, vascular defects, and TOF [40,41]. Deletion of 10q26.3 and chromosome 6 was observed in two patients (cardio-32.A and cardio-3.A) with septal defects and TGA, respectively. 10q26 deletion syndrome has been reported in cases with septal defects [42] and patent ductus arteriosus [43]. In total, 16 patients with CHD had chromosomal abnormalities, mainly as part of a syndrome with multiple systemic and developmental comorbidities. Most had trisomy 21, a 7q11.23 deletion, and a 22q11.2 deletion, while rare CNVs were less common.

From the WES analysis, we identified four pathogenic or likely pathogenic variants in five different genes in six patients with CHD. A de novo pathogenic variant c.2883-1G>T in *ROBO1* was detected in a patient from Sri Lanka (cardio-12.A) with multiple cardiac defects and an extracardiac phenotype of congenital hydronephrosis. The *ROBO1* gene pathway interacts with other genes that play an essential role in the pathogenesis of CHD, such as *TBX1*, *TBX5*, *NOTCH1*, and *NKX2.5* [44]. Several case studies have reported loss of function of SNVs in *ROBO1* among patients with septal defects and TOF along with extracardiac phenotype [45]. We report the first case of a patient with multiple cardiac defects, including conotruncal and septal defects, with a detected variant in *ROBO1*. Moreover, the CADD score for this variant was 27.3, which supports its potential role in the etiology of the disease in the patient (cardio-12.A). Exploring a correlation between genotype and phenotype may be challenging, as the *ROBO1* gene has not yet been fully studied. Moreover, in silico analysis may not be sufficient to obtain a definitive answer regarding its role in the pathogenicity of CHD, given the fact that this variant is detected in a patient with multiple cardiac defects for the first time. A functional assessment of the variant needs to be pursued in order to confirm its possible pathogenicity and to determine the mechanisms by which it leads to disease.

Variant c.243C>G in *SLC2A10* was found in a homozygous state in a patient with ATS from Yemen coming from a consanguineous family. The variant c.243C>G has been previously identified in a homozygous state in 15 unrelated patients from Qatar belonging to Bedouin tribes with the same clinical manifestation of ATS [46], as well as in several Mediterranean families [47]. The variant c.243C>G has not been detected in the gnomAD or GME datasets; however, it has been detected in the Qatar 1000 genome project, with a MAF of 0.007. To our knowledge, this is the first time this variant is encountered in a patient from Yemen. Given its recessive pattern of inheritance and the high consanguinity rate among Arabs compared to other populations [3], it is possible that this variant is more prevalent in Arabs and is a founder mutation that causes disease in homozygosity.

Both variants c.817+1G>C in *SMAD6* and c.6292C>T in *CHD7* were likely pathogenic and were identified in a heterozygous state in cardio-15.A and cardio-27.A, respectively. It has been reported that *SMAD6* plays a role in the cardiac development in chick models, where the induction of *SMAD6* and *Nkx2-5* contributed to the pathogenesis of CHD. Nonsynonymous variants in *SMAD6* have been observed in cardiovascular malformations, such as a bicuspid aortic valve with mild aortic stenosis and aortic coarctation [48]. Considering the supposed role of the gene in cardiovascular malformation, it is possible that c.817+1G>C in *SMAD6* had influenced the manifestation of Shone’s complex in our patient.

Variant c.6292C>T in *CHD7* was detected in cardio-27.A, who had syndromic CHD. Variants of loss of function in *CHD7* are associated with CHARGE syndrome (OMIM# 214800), a combination of multiple congenital malformations in which 70–92% of cases have cardiac defects [49]. Variable expressivity has been observed in patients with positive *CHD7* variants, where patients did not meet the diagnostic criteria for CHARGE syndrome [50]. This finding highlights the difficulty in establishing a clear genotype–phenotype correlation among patients positive for *CHD7* variants, as well as the possibility of missing *CHD7*-positive cases who fail to meet the diagnostic criteria for CHARGE syndrome.

We detected eight variants classified as likely benign in five patients with CHD. Five variants followed an autosomal recessive pattern of inheritance; they were detected in a homozygous state in three patients (cardio-15.A, cardio-18.A, and cardio-31.A) coming from consanguineous families. Variant p.Val1457Ile in *CCDC141* was observed in one patient (cardio-15.A) with Shone’s complex, who also had a pathogenic variant in *SMAD6*. Variants in *CCDC141* have been linked to cardiovascular malformations and were observed to play a role in the cardiac development in zebrafish [51]. Variants c.6035C>T and c.1751A>C were present in the *CMYA5* gene in one patient (cardio-18.A). Both variants were seen in homozygosity and were inherited from both parents. These variants were also detected in unaffected siblings, but in a heterozygous state. It was noted that all the likely benign variants found in our cohort had a higher MAF in the GME and Qatar 1000 genome control databases compared to the gnomAD database. This may be influenced by the high consanguinity rate in the Middle Eastern population, considering the fact that five variants followed a recessive pattern of inheritance. Consequently, there is a need for further assessments of the role of c.6035C>T and c.1751A>C in *CMYA5* and c.3401G>T in *IFT172* to determine their effect on the function of the corresponding genes, given the contradictory results of in silico analyses and their relatively high frequency in the Middle Eastern population.

We reported 51 variants classified as US in 49 genes of 23 patients with CHD. Fourteen variants were de novo, while the rest were inherited from either one parent or both. We reported five US variants that were inherited from both parents in an autosomal recessive pattern, including c.43A>T in BUB1, c.926A>G in ITGAL, c.884A>G in SMYD4, c.4867C>A in SHROOM3, and c.937G>T in GLA. Homozygous variantc.43A>T in BUB1 was detected in a patient from Qatar (cardio-18.A) coming from a consanguineous family, who had aortic stenosis and left ventricular hypertrophy. Interestingly, this variant has been exclusively reported in the Qatar 1000 genome project among individuals from Qatar, but not in the gnomAD or GME datasets. In silico variant effect predictions, including SIFT and PolyPhen, suggested the possibility of a benign effect, but the CADD score for this variant was 21.2. Using bioinformatic computational analysis, it was shown that *BUB1* plays a potential role in CHD. However, its exact role in the pathogenesis of CHD remains unknown, and no reported cases have supported this claim [52].

Variant c.926A>G in *ITGAL* was detected in a homozygous state in a patient from Qatar (cardio-38.A) coming from a consanguineous family. This variant is novel since it has not been previously reported in any control and clinical datasets. SIFT and PolyPhen consider the variant deleterious and possibly damaging; however, limited published data explain the role of *ITGAL* in the context of cardiac defects. One study has suggested that it plays an indirect role in cardiovascular diseases [53]. Moreover, the variant c.884A>G in *SMYD4* was seen in homozygosity in a patient from Sudan (cardio-31.A) with HLHS. The variant was considered deleterious and damaging based on SIFT and PolyPhen, as well as the CADD of 28.1. Nevertheless, the variant was detected in all control datasets that we analyzed. In the gnomAD dataset, no “probably healthy” subject was homozygous for this variant, which increases the possibility that it may cause HLHS.

Variant c.937G>T in the *GLA* gene, located in the X chromosome, was observed in a patient (cardio-50.A) with TOF; it was inherited from the mother in a UPD pattern. This variant has been detected in other patients with hypertrophic cardiomyopathy, Fabry disease, angiokeratoma corporis diffusum, and sudden unexplained death (Table S3). Other published case studies have shown that loss-of-function variants in *GLA* are associated with hypertrophic cardiomyopathy [54]. The difference in phenotype between our patient and previously reported cases with the same variant highlights the difficulty of establishing genotype–phenotype correlations and, consequently, establishing a genetic diagnosis. Overall, many US variants were not fully able to explain the CHD phenotype in our patients, despite the fact that they were located in genes that play a potential role in the pathogenesis of CHD.

In our study, explaining the CHD phenotype in the context of SNVs was challenging. We identified 13 variants shared between our patients and patients from other populations with a variable range of clinical phenotypes; none of them showed a phenotype similar to our patients. Possible explanations may be the variable expressivity and penetrance of the variant, the influence of modifier genes, and the effect of ethnic background. Around 400 genes have been linked to the pathogenesis of CHD, and the American Heart Association (AHA) has issued a statement [1] discussing the genetic architecture of CHD in the context of more than 200 genes and loci. Of them, 27 genes were classified as very commonly associated, 16 as associated, and 21 as occasionally associated. In the Sidra registry cohort, we were able to only capture variants in six genes *(KRAS, CHD7, KMT2D, DYNC2H1, SMAD6, CRELD1)* [1].

This limited number of fully explained genes is of no value for clinical practice and genetic counseling, where many other genes are frequently seen, especially in patients with an isolated CHD phenotype or those from understudied populations. We observed several variants inherited in a homozygous pattern among Arab consanguineous families, such as c.243C>G in *SLC2A10, c.6035C>T* and *c.1751A>C* in *CMYA5, c.1606G>A* and *c.131G>T* in *DNAAF3, c.43A>T* in *BUB1, c.926A>G* in *ITGAL,* and *c.884A>G* in *SMYD4.* MAF values indicate a relatively higher prevalence of those variants in the Arab population. For example, c.243C>G in *SLC2A10* has a MAF of 0.0071 in the Qatar 1000 genome, while this variant was not detected in other control datasets. Similar observations were noted for c.6035C>T and c.1751A>C in *CMYA5,* as well as for c.1606G>A and c.131G>T in *DNAAF*, all of which have a higher MAF in the GME and Qatar 1000 genome dataset compared to gnomAD. Given the endogamous nature of the Middle Eastern population, it is expected that such a variant may have a higher allele frequency than reported. Therefore, the role of these variants in the pathogenesis of CHD must be rigorously assessed.

Our study has some limitations that may have biased our results and their interpretation, including a small sample size, the inability to determine the inheritance of some variants due to the fact that parents were not included in the WES analysis component of the study, as well as the inability to access the original reports for some patients. On the other hand, our study focused on examining nuclear DNA rather than mitochondrial DNA; therefore, there is a possibility that mitochondrial variants were missed in unexplained cases [55,56]. Considering the above, we emphasize the importance of expanding research efforts in the field of CHD genetic architecture.

## 5. Conclusions

We identified a potential genetic etiology of CHD in 40 out of 52 studied patients with CHD. Sixteen of them were explained by cytogenetic abnormalities, while 24 patients had one or more variants identified by our WES analysis that may have contributed to the pathogenesis of CHD. We identified four pathogenic/likely pathogenic SNVs that could explain the phenotype of CHD in four patients. We detected some recessive variants, such as c.884A>G in *SMYD6*, which appear to have higher frequencies in Arab and Middle Eastern populations compared to others. However, the exact role of these variants in cardiac pathogenesis has not yet been established. Future in vitro and in vivo functional assessments are needed to better understand the role of these variants in the pathogenicity of CHD.

## Figures and Tables

**Table 1:** Results of the chromosomal testing conducted in 27 patients with CHD as part of their clinical evaluation.

Patient	Cardiac Phenotype	Extracardiac Phenotype	Chromosomal Abnormality	Genes Encompassed	Associated Condition	Interpretation of Test Results	Parental Testing
Cardio-1.A	ASD, VSD	Hypotonia, dysmorphic features, developmental delay	47, XY, +21	Gain of one full copy of chromosome 21	Down Syndrome	Pathogenic	No record
Cardio-3.A	TGV	Vertebral abnormalities, anal atresia, cardiac abnormalities, tracheoesophageal fistula, renal anomalies, limb defects	Chromosome 6 deletion *	Unknown	Unknown	Unknown	No record
Cardio-4.A	TOF, PA	Thymus hypoplasia, compromised immune system, absent left kidney, idiopathic left club foot, global developmental delay with central hypotonia	22q11.2 deletion	40 genes (*TBX1* and *COMT*)	DiGeorge Syndrome	Pathogenic	Mother is normal; father has no records
Cardio-6.A	VSA	Congenital nasolacrimal duct obstruction, esotropia, failure to thrive, hypothyroidism, supraventricular tachycardia, thrombocytopenia	22q11.2 deletion	40 genes (*TBX1* and *COMT*)	DiGeorge Syndrome	Pathogenic	No records
Cardio-9.A	ASD, DORV, PA, Hypoplastic mitral valve and left ventricle	Hypotension, acidosis, bradycardia, severe developmental delay, seizures	9q34.3 deletion	*SNAPC4, PMPCA, INPP5E, SEC16A, NOTCH1*	Adams Oliver Syndrome	Likely Pathogenic	Mother is normal; father is inconclusive
Cardio-28.A	TOF	Acute renal failure, fluid overload, skin pigmentation, undescended testicles	Mosaic 47, XY, +14	Gain of one full copy of chromosome 14 in some somatic cells	Mosaic trisomy 14	Pathogenic	Parents are normal
Cardio-30.A	TOF	Bilateral hydronephrosis, hypotonia, dysmorphic features	47, XY, +21	Gain of one full copy of chromosome 21	Down Syndrome	Pathogenic	No records
Cardio-32.A	VSD	Dysphagia, gastroesophageal reflux disease, failure to thrive, global developmental delay, hypotonia, central sleep apnea, right ankle contracture, asymmetric leg length	7q11.23 deletion,10q26.3 deletion,	*ELN, LIMK1, BAZ1B, CLIP2, GTF2IRD, NSUN5, CLDN4, EIF4H, LAT2, MLXIPL, TBL2, WBSCR18, WBSCR22, WBSCR27*—Not defined for 10q26.3 deletion	William Syndrome and 10q26.3 deletion	Pathogenic	Parents are normal
Cardio-33.A	ASD	Polydactyly, dysmorphic features, delayed motor development	47, XY, +21	Gain of one full copy of chromosome 21	Down Syndrome	Pathogenic	Parents are normal
Cardio-37.A	TOF, PS	Failure to thrive, hypothyroidism	47, XY +8 22q11.21 deletion	Gain of one full copy of chromosome 8	DiGeorge syndrome and mosaic trisomy 8	Pathogenic	No records
Cardio-39.A	VSD	Dysmorphic features, hypotonia	47, XY, +21	Gain of one full copy of chromosome 21	Down Syndrome	Pathogenic	No records
Cardio-44.A	AS, PS	Anal stenosis, dysmorphic features	7q11.23 deletion	*ELN, LIMK1, BAZ1B, CLIP2, GTF2IRD, NSUN5, CLDN4, EIF4H, LAT2, MLXIPL, TBL2, WBSCR18, WBSCR22, WBSCR27*	William Syndrome	Pathogenic	No records
Cardio-45.A	HLHS	Depressed nasal bridge, developmental delay	8p11.21 duplication	Not reported	Not specified	Unknown	Mother is normal
Cardio-56.A	ASD, PA, TAPVD, Heterotaxy	Bowel obstruction, osteomyelitis, asplenia	16p11.2 deletion	*SH2B1* gene	Not specified	Likely benign	Mother is normal; father has the same deletion
Cardio-60.A	ASD	Laryngomalacia, swallowing dysfunction	47, XX, +21	Gain of one full copy of chromosome 21	Down Syndrome	Pathogenic	No records
Cardio-62.A	ASD	Hypotonia, dysmorphic features	47, XY, +21	Gain of one full copy of chromosome 21	Down Syndrome	Pathogenic	No records

**ASD**: atrial septal defect; **DORV:** double outlet right ventricle; **HLHS**: hypoplastic left heart syndrome; **PA:** pulmonary atresia; **PS**: pulmonary stenosis; **TAPVD:** total anomalous pulmonary venous drainage; **TGV**: transposition of the great arteries; **TOF**: tetralogy of Fallot; **VSD**: ventricular septal defect. *: the detailed laboratory report is not available for this patient.

**Table 2:** Pathogenic/likely pathogenic variants detected in CHD patients.

Gene	Amino Acid Change	Nucleotide Change	Variant Type	Variant Impact	Familial Segregation	Zygosity	Inheritance	ACMG/AMP Classification	CHD Phenotype	Extra Phenotype	Patient
*ROBO1*	-	c.2883-1G>T	SNP	splice acceptor	de novo	Heterozygous	None	Pathogenic	Multiple CHD	None	Cardio-12.A
*SMAD6*	-	c.817+1G>C	SNP	splice site donor	de novo	Heterozygous	None	Likely pathogenic	Shone’s complex	None	Cardio-15.A
*SLC2A10*	p.Ser81Arg	c.243C>G	SNP	Missense	AR	Homozygous	Both parents	Likely pathogenic	ATS	None	Cardio-5.A
*CHD7*	p.Arg2098 *	c.6292C>T	SNP	stop gained	de novo	Heterozygous	None	Pathogenic	TOF	Prolonged QT interval, hearing loss	Cardio-27.A

**AR**: autosomal recessive; **ATS**: arterial tortuosity syndrome; **CHD**: congenital heart disease; **TOF:** tetralogy of Fallot. * means that the amino acid Arginine2098 is predcited to change to a stop codon.

## Data Availability

For reasons of privacy and confidentiality, the data from this study are available from the corresponding authors upon reasonable request.

## Supplementary Materials

The following supporting information can be downloaded at: https://www.mdpi.com/article/10.3390/genes13081369/s1; Figure S1. Stratification of variants identified in 24 patients with CHD. Table S1: Positive results of chromosomal investigation done for the 27 of our patient’s cohort as part of their clinical evaluation. Table S2: Summary of the novel variants identified in 24 CHD patients through whole exome sequencing (WES) analysis. Table S3: Summary of the shared variants identified in 24 CHD patients through whole exome sequencing (WES) analysis. Table S4: Summary of the variants identified which were classified based on ACMG/AMP guideline as (a) pathogenic/likely pathogenic, (b) likely benign and (c) variants of uncertain significance. References [57,58,59,60,61,62,63,64,65,66,67,68,69,70,71] are cited in the Supplementary Materials.

## References

[1] Pierpont M.E., Brueckner M., Chung W.K., Garg V., Lacro R.V., McGuire A.L., Mital S., Priest J.R., Pu W.T., Roberts A. (2018). Genetic basis for congenital heart disease: Revisited: A scientific statement from the American Heart Association. Circulation.

[2] Fahed A.C., Gelb B.D., Seidman J., Seidman C.E. (2013). Genetics of congenital heart disease: The glass half empty. Circ. Res..

[3] Tadmouri G.O., Nair P., Obeid T., Al Ali M.T., Al Khaja N., Hamamy H.A. (2009). Consanguinity and reproductive health among Arabs. Reprod. Health.

[4] Hamdan M.A., Chedid F., Bekdache G.N., Begam M., Alsafi W., Sabri Z., Mirghani H.M. (2015). Perinatal outcome of congenital heart disease in a population with high consanguinity. J. Perinat. Med..

[5] Aburawi E.H., Aburawi H.E., Bagnall K.M., Bhuiyan Z.A. (2015). Molecular insight into heart development and congenital heart disease: An update review from the Arab countries. Trends Cardiovasc. Med..

[6] Robida A., Folger G.M., Hajar H.A. (1997). Incidence of congenital heart disease in Qatari children. Int. J. Cardiol..

[7] Iyad A., Fares A., Laila T. (2017). Incidence of congenital heart disease in jordanian children born at jordan university hospital: A seven-year retrospective study. Jordan Med. J..

[8] Bitar F.F., Baltaji N., Dbaibo G., Yunis K., Obeid M. (1999). Congenital heart disease at a tertiary care center in Lebanon. Middle East J. Anaesthesiol..

[9] Subramanyan R., Joy J., Venugopalan P., Sapru A., Khusaiby S.A. (2000). Incidence and spectrum of congenital heart disease in Oman. Ann. Trop. Paediatr..

[10] Zaqout M., Aslem E.S., Oweida F.S., De Wolf D. (2014). Prevalence of congenital heart disease among Palestinian children born in the Gaza Strip. Cardiol. Young.

[11] Al-Fahham M.M., Ali Y.A. (2021). Pattern of congenital heart disease among Egyptian children: A 3-year retrospective study. Egypt Heart J..

[12] Hammami O., Salem B., Boujemaa Z., Chebbi Y., Aoun S., Meddeb I., Abid F., Gandoura N. (2007). Epidemiologic and clinical features of congenital heart diseases in children at the Bizerta Hospital. Tunis. Med..

[13] World Health Organization, Geneva, Switzerland (2020). ICD-10 Coding Manual-List of All Reportable Congenital Malformations.

[14] Sundaresan V., Mambetisaeva E., Andrews W., Annan A., Knöll B., Tear G., Bannister L. (2004). Dynamic expression patterns of Robo (Robo1 and Robo2) in the developing murine central nervous system. J. Comp. Neurol..

[15] Faust G.G., Hall I.M. (2014). SAMBLASTER: Fast duplicate marking and structural variant read extraction. Bioinformatics.

[16] Li H., Handsaker B., Wysoker A., Fennell T., Ruan J., Homer N., Marth G., Abecasis G., Durbin R. (2009). The sequence alignment/map format and SAMtools. Bioinformatics.

[17] McKenna A., Hanna M., Banks E., Sivachenko A., Cibulskis K., Kernytsky A., Garimella K., Altshuler D., Gabriel S., Daly M. (2010). The Genome Analysis Toolkit: A MapReduce framework for analyzing next-generation DNA sequencing data. Genome Res..

[18] Tan A., Abecasis G.R., Kang H.M. (2015). Unified representation of genetic variants. Bioinformatics.

[19] Cingolani P., Platts A., Wang L.L., Coon M., Nguyen T., Wang L., Land S.J., Lu X., Ruden D.M. (2012). A program for annotating and predicting the effects of single nucleotide polymorphisms, SnpEff: SNPs in the genome of Drosophila melanogaster strain w1118; iso-2; iso-3. Fly.

[20] Razali R.M., Rodriguez-Flores J., Ghorbani M., Naeem H., Aamer W., Aliyev E., Jubran A., Clark A.G., Fakhro K.A., Mokrab Y. (2021). Thousands of Qatari genomes inform human migration history and improve imputation of Arab haplotypes. Nat. Commun..

[21] Fakhro K.A., Staudt M.R., Ramstetter M.D., Robay A., Malek J.A., Badii R., Al-Marri A.A.-N., Khalil C.A., Al-Shakaki A., Chidiac O. (2016). The Qatar Genome: A Population-specific Tool for Precision Medicine in the Middle East. Hum. Genome Var..

[22] Scott E.M., Halees A., Itan Y., Spencer E.G., He Y., Azab M.A., Gabriel S.B., Belkadi A., Boisson B., Abel L. (2016). Characterization of Greater Middle Eastern genetic variation for enhanced disease gene discovery. Nat. Genet..

[23] Doğan T. (2018). HPO2GO: Prediction of human phenotype ontology term associations for proteins using cross ontology annotation co-occurrences. PeerJ.

[24] Rentzsch P., Witten D., Cooper G.M., Shendure J., Kircher M. (2019). CADD: Predicting the deleteriousness of variants throughout the human genome. Nucleic Acids Res..

[25] Li Q., Wang K. (2017). InterVar: Clinical interpretation of genetic variants by the 2015 ACMG-AMP guidelines. Am. J. Hum. Genet..

[26] Dadvand P., Rankin J., Shirley M.D., Rushton S., Pless-Mulloli T. (2009). Descriptive epidemiology of congenital heart disease in Northern England. Paediatr. Perinat. Epidemiol..

[27] Hartman R.J., Rasmussen S.A., Botto L.D., Riehle-Colarusso T., Martin C.L., Cragan J.D., Shin M., Correa A. (2011). The contribution of chromosomal abnormalities to congenital heart defects: A population-based study. Pediatr. Cardiol..

[28] Pradat P. (1992). Epidemiology of major congenital heart defects in Sweden, 1981–1986. J. Epidemiol. Community Health.

[29] Abbag F.I. (2006). Congenital heart diseases and other major anomalies in patients with Down syndrome. Saudi Med. J..

[30] Stoll C., Dott B., Alembik Y., Roth M.-P. (2015). Associated congenital anomalies among cases with Down syndrome. Eur. J. Med. Genet..

[31] Cirillo E., Giardino G., Grasso F., Gallo V., Pignata C. (2022). DiGeorge Syndrome. Genetic Syndromes: A Comprehensive Reference Guide.

[32] Griffith E., Alfonso N., Hehmeyer K., Pope K. (2022). Genetic syndromes and their associations with congenital heart disease. Prog. Pediatric Cardiol..

[33] Sherer D., Dalloul M., Pinard V., Sheu J., Abulafia O. (2017). Fetal trisomy 8 mosaicism associated with truncus arteriosus Type I. Ultrasound Obstet. Gynecol..

[34] Alkuraya F.S., Harris D.J. (2005). Trisomy 8 mosaicism in a patient with heterotaxia. Birth Defects Res. Part A Clin. Mol. Teratol..

[35] Belengeanu V., Boia M., Farcas S., Popa C., Stoian M., Belengeanu A. (2010). Trisomy 8 mosaicism with atypical phenotypic features. J. Pediatr..

[36] Fujimoto A., Allanson J., Crowe C.A., Lipson M.H., Johnson V.P. (1992). Natural history of mosaic trisomy 14 syndrome. Am. J. Med. Genet..

[37] Fran Lynch M., Fernandes C.J., Shaffer L.G., Potocki L. (2004). Trisomy 14 mosaicism: A case report and review of the literature. J. Perinatol..

[38] Kunst G., Gillbe C. (2005). General anesthesia for cardiac catheterization in a child with trisomy 14 mosaicism. Anesth. Analg..

[39] Tunca Y., Wilroy R.S., Kadandale J.S., Martens P.R., Gunther W.M., Tharapel A.T. (2000). Hypomelanosis of Ito and a ‘mirror image’whole chromosome duplication resulting in trisomy 14 mosaicism. Annales de Genetique.

[40] Lin A.E., Westgate M.-N., van der Velde M.E., Lacro R.V., Holmes L.B. (1998). Adams-Oliver syndrome associated with cardiovascular malformations. Clin. Dysmorphol..

[41] Algaze C., Esplin E.D., Lowenthal A., Hudgins L., Tacy T.A., Selamet Tierney E.S. (2013). Expanding the phenotype of cardiovascular malformations in Adams–Oliver syndrome. Am. J. Med. Genet. A.

[42] Irving M., Hanson H., Turnpenny P., Brewer C., Ogilvie C.M., Davies A., Berg J. (2003). Deletion of the distal long arm of chromosome 10; is there a characteristic phenotype? A report of 15 de novo and familial cases. Am. J. Med. Genet. A.

[43] Yatsenko S., Kruer M., Bader P., Corzo D., Schuette J., Keegan C., Nowakowska B., Peacock S., Cai W., Peiffer D. (2009). Identification of critical regions for clinical features of distal 10q deletion syndrome. Clin. Genet..

[44] Medioni C., Bertrand N., Mesbah K., Hudry B., Dupays L., Wolstein O., Washkowitz A.J., Papaioannou V.E., Mohun T.J., Harvey R.P. (2010). Expression of Slit and Robo genes in the developing mouse heart. Dev. Dyn..

[45] Kruszka P., Tanpaiboon P., Neas K., Crosby K., Berger S.I., Martinez A.F., Addissie Y.A., Pongprot Y., Sittiwangkul R., Silvilairat S. (2017). Loss of function in ROBO1 is associated with tetralogy of Fallot and septal defects. J. Med. Genet..

[46] Faiyaz-Ul-Haque M., Zaidi S., Wahab A., Eltohami A., Al-Mureikhi M., Al-Thani G., Peltekova V., Tsui L.C., Teebi A.S. (2008). Identification of a p. Ser81Arg encoding mutation in SLC2A10 gene of arterial tortuosity syndrome patients from 10 Qatari families. Clin. Genet..

[47] Callewaert B., Willaert A., Kerstjens-Frederikse W., De Backer J., Devriendt K., Albrecht B., Ramos-Arroyo M., Doco-Fenzy M., Hennekam R., Pyeritz R. (2008). Arterial tortuosity syndrome: Clinical and molecular findings in 12 newly identified families. Hum. Mutat..

[48] Tan H.L., Glen E., Töpf A., Hall D., O’Sullivan J.J., Sneddon L., Wren C., Avery P., Lewis R.J., ten Dijke P. (2012). Nonsynonymous variants in the SMAD6 gene predispose to congenital cardiovascular malformation. Hum. Mutat..

[49] Corsten-Janssen N., Kerstjens-Frederikse W.S., du Marchie Sarvaas G.J., Baardman M.E., Bakker M.K., Bergman J.E., Hove H.D., Heimdal K.R., Rustad C.F., Hennekam R.C. (2013). The cardiac phenotype in patients with a CHD7 mutation. Circ. Cardiovasc. Genet..

[50] Jongmans M., Admiraal R., Van Der Donk K., Vissers L., Baas A., Kapusta L., van Hagen J.M., Donnai D., De Ravel T., Veltman J. (2006). CHARGE syndrome: The phenotypic spectrum of mutations in the CHD7 gene. J. Med. Genet..

[51] Tillman S. (2021). Congenital Heart Defects and the Expression of Ccdc141. Honors Thesis.

[52] Zhang J., An X., Sun X., Yu K., Gong T. Screening of Candidate Key Genes Associated with Congenital Heart Disease Using Bioinformatics Data Analysis. Proceedings of the 2020 International Conference on Modeling, Big Data Analytics and Simulation (MBDAS2020).

[53] Kim D.S., Burt A.A., Crosslin D.R., Robertson P.D., Ranchalis J.E., Boyko E.J., Nickerson D.A., Furlong C.E., Jarvik G.P. (2013). Novel common and rare genetic determinants of paraoxonase activity: FTO, SERPINA12, and ITGAL [S]. J. Lipid Res..

[54] Bittencourt M.I. (2019). Description of a New GLA Gene Variant in a Patient with Hypertrophic Cardiomyopathy. Is it Fabry Disease?. Arq. Bras. Cardiol..

[55] Lee S.R., Han J. (2017). Mitochondrial mutations in cardiac disorders. Mitochondrial Dyn. Cardiovasc. Med..

[56] Majamaa-Voltti K., Peuhkurinen K., Kortelainen M.-L., Hassinen I.E., Majamaa K. (2002). Cardiac abnormalities in patients with mitochondrial DNA mutation 3243A> G. BMC Cardiovasc. Disord..

[57] Savarese M., Maggi L., Vihola A., Jonson P.H., Tasca G., Ruggiero L., Bello L., Magri F., Giugliano T., Torella A. (2018). Interpreting Genetic Variants in Titin in Patients with Muscle Disorders. JAMA Neurol..

[58] Faiyaz-Ul-Haque M., Zaidi S.H.E., Al-Sanna N., Alswaid A., Momenah T., Kaya N., Al-Dayel F., Bouhoaigah I., Saliem M., Tsui L.-C. (2009). A Novel Missense and a Recurrent Mutation in Slc2a10 Gene of Patients Affected with Arterial Tortuosity Syndrome. Atherosclerosis.

[59] [VCV000773847.2]. National Center for Biotechnology Information. https://www.ncbi.nlm.nih.gov/clinvar/variation/VCV000773847.2.

[60] National Center for Biotechnology Information [Vcv000167010.9]. ClinVar. https://www.ncbi.nlm.nih.gov/clinvar/variation/VCV000167010.9.

[61] [VCV000722668.2]. National Center for Biotechnology Information. ClinVar. https://www.ncbi.nlm.nih.gov/clinvar/variation/VCV000722668.2.

[62] Information, National Center for Biotechnology Clinvar. [Vcv000722667.2]. https://www.ncbi.nlm.nih.gov/clinvar/variation/VCV000722667.2.

[63] Zhu T., Gong X., Bei F., Ma L., Sun J., Wang J., Qiu G., Sun J., Sun Y., Zhang Y. (2021). Primary Immunodeficiency-Related Genes in Neonatal Intensive Care Unit Patients with Various Genetic Immune Abnormalities: A Multicentre Study in China. Clin. Transl. Immunol..

[64] Sun Y., Sun J., Li N., Cai C., Gong X., Ma L. (2020). Phenotypic Spectrum of Typical Charge Syndrome in a Chinese Male Neonate: A Case Report. Transl. Pediatr..

[65] National Center for Biotechnology Information [VCV000136948.9]. Clinvar. https://www.ncbi.nlm.nih.gov/clinvar/variation/VCV000136948.9.

[66] [VCV000707217.7]. National Center for Biotechnology Information. https://www.ncbi.nlm.nih.gov/clinvar/variation/VCV000707217.7.

[67] [VCV000129948.9]. National Center for Biotechnology Information. ClinVar. https://www.ncbi.nlm.nih.gov/clinvar/variation/VCV000129948.9.

[68] Kanthi A., Hebbar M., Bielas S.L., Girisha K.M., Shukla A. (2019). Bi-Allelic C. 181_183deltgt in Btb Domain of Klhl7 Is Associated with Overlapping Phenotypes of Crisponi/Ciss1-Like and Bohring-Opitz Like Syndrome. Eur. J. Med. Genet..

[69] [VCV000216489.13]. National Center for Biotechnology Information. https://www.ncbi.nlm.nih.gov/clinvar/variation/VCV000216489.13.

[70] National Center for Biotechnology Information [VCV000258032.9]. https://www.ncbi.nlm.nih.gov/clinvar/variation/VCV000258032.9.

[71] [VCV000010738.32]. National Center for Biotechnology Information. ClinVar. https://www.ncbi.nlm.nih.gov/clinvar/variation/VCV000010738.32.

