# Supplementary material for: Investigation of Genetic Causes in Patients with Congenital Heart Disease in Qatar: Findings from the Sidra Cardiac Registry
Source: Genes (Basel). 2022 Jul 30;13(8):1369. doi: 10.3390/genes13081369 (PMC9407366; doi:10.3390/genes13081369)
Supplement: Supplementary file 1 [file genes-13-01369-s001.zip › supplementary tables/Table S3.pdf]

Table S3 :Summary of the shared variants identified in 24 CHD patients through whole exome sequencing (WES) analysis

Patient ID	Cardiac Phenotype	Extra Cardiac phenotype	Age	Gender	Country of origin	Gene	Protein change	Nucleotide change	Gemini segregation	Zygosity	Inheritance	ClinVar	ACMG/AMP classification	ACMG/AMP Sub classification	Reported in other studies	Reported cases country of origin	*References*
Cardio-2.A	TOF	None	6 months	F	Qatar	*TTN*	p.Asn32797Ser	c.98390A>G	CH	Heterozygous	Paternal	Conflict interpretation	Likely benign	BP1, BP6, PP3	Late onset progressive distal myopathy	European	[1]
Cardio-5.A	ATS	None	2 months	M	Yemen	*SLC2A10*	p.Ser81Arg	c.243C>G	AR	Homozygous	Both parents	Pathogenic	Likely pathogenic	PM1,PM2,PP3,PP5	ATS	Qatari, KSA	[2, 3]
Cardio-11.A	ASD	Squint, polydactyly	3 months	F	India	*PRR12*	p.Gly1813Arg	c.5437G>A	AR	Homozygous	Both parents	None	US	PM1,BP1	none	-	-
Cardio-12.A	multiple CHD	Failure to thrive	20 days	F	Sri Lanka	*KMT2D*	p.Pro3665Ala	c.10993C>G	CH	Heterozygous	Maternal	Conflicting interpretation	US	BP1	Kabuki syndrome	Unknown	[4]
Cardio-15.A	Shone's complex (hypoplastic transverse arch, VSD, PDA)	None	7 days	F	Pakistan	*FLT1*	-	c.1437-6delT	*de novo*	Heterozygous	None	None	none	-	none	-	-
						CCDC141	p.Val1457Ile	-	AR	Homozygous	Both parents	Likely Benign	Likely benign	BS1,BP6	Reported (unknown condition)	-	[4]
Cardio-16.A	TOF	None	9 months	M	Sudan	*SORBS2*	p.Phe1188Leu	c.3564C>A	Could not be identified	Heterozygous	Not reported *	None	US	PM1	none	-	-
						DYNC2H1	p.Val1899Ile	c.5695G>A	CH	Heterozygous	Maternal	Conflicting interpretation	US	PM1,PM2	Short rib Thoracic dysplasia	Multiple submitters(unknown ethnicities)	[5]
						BMP10	p.Thr200Ser	c.599C>G	*de novo*	Heterozygous	None	None	US	PM1,BS1	none	-	-
						KYNU	p.Thr25Met	c.74C>T	*de novo*	Heterozygous	None	Benign	US	PM1,PM2,BP6	none	-	-
						PLCD4	p.Val364Met	c.1090G>A	*de novo*	Heterozygous	None	None	US	PM1,BP4	none	-	-
						MLIP	p.Pro459Arg	c.1376C>G	*de novo*	Heterozygous	None	None	US	PM1	none	-	-
						PBX3	p.His401Gln	c.1203T>A	*de novo*	Heterozygous	None	None	US	PM1,PM2	none	-	-
						OR51E1	p.Ala156Thr	c.466G>A	*de novo*	Heterozygous	None	None	US	PM1	none	-	-
Cardio-18.A	Aortic stenosis & insufficiency, mild LVH, frequent PACs	none	18 years	M	Qatari	*CMYA5*	p.Ser2012Phe	c.6035C>T	AR	Homozygous	One copy was maternally inherited(all siblings are carrriers)**	Benign	likely benign	BS1,BP6	Reported (unknown condition)	Unknown	[6]
						CMYA5	p.Glu584Ala	c.1751A>C	AR	Homozygous	One copy was maternally inherited(all siblings are carriers)**	Benign	likely benign	PM1,PM2	Reported (unknown comnditon)	Unknown	[7]
Cardio-27.A	TOF	Prolonged QT interval, hearing loss		F	Yemen	*CHD7*	p.Arg2098*	c.6292C>T	*de novo*	Heterozygous	none	Pathogenic	Pathogenic	PVS1,PM2,PP3,PP5	Immune deficiency, CHARGE syndrome	China	[8, 9]
Cardio-31.A	HLH	None	6 years	F	Sudan	*DNAH11*	c.9946-7T>C	-	AR	Homozygous	One copy paternally inherited ***	None	None	PM2	none	-	-
						DNAAF3	p.Val536Met	c.1606G>A	AR	Homozygous	One copy paternally inherited ***	Benign/likely benign	Likely Bening	PM1,BS1,BP1,BP4,BP6	Hypertrophic cardiomyopathy, Dilated Cardiomyopathy, Recessive, Familial restrictive cardiomyopathy, Primary ciliary dyskinesia	-	-
						DNAAF3	p.Trp44Leu	c.131G>T	AR	Homozygous	One copy paternally inherited ***	Benign	likely benign	PM1,BS1,BP1,BP6	Primary ciliary dyskinesia	-	-
						COL5A2	p.Arg956Pro	c.2867G>C	CH	Heterozygous	Paternal	Benign	Likely benign	PM1,BS1,BP6	Ehler Danlos Syndrome, cardiovascular phenotype	Unknown	[10]
						SMYD4	p.Tyr295Cys	c.884A>G	AR	Homozygous	One copy paternally inherited ***	None	US	PM1	none	-	-
Cardio-36.A	TOF	None	5 months	F	Sudan	*IFT172*	p.Val1204Leu	c.3610G>C	CH	heterozygous	Maternal	Benign /likely benign	US	BP6	Retinitis pigmentosa 71,Short-rib thoracic dysplasia 10 with or without polydactyly	Unknown	[11]
						IFT172	p.Arg1134Leu	c.3401G>T	CH	Heterozygous	Paternal	Benign/likely benign	Likely benign	BS1,BP6	Retinitis pigmentosa 71,Short-rib thoracic dysplasia 10 with or without polydactyly	Unknown	[11]
						SLC24A4	p.Gln22His	c.66A>T	CH	Heterozygous	Paternal	None	US	PM1,PM2	none	-	-
						ATP10D	p.Arg266His	c.797G>A	AD	Heterozygous	Maternal(brother is also carrier)	None	US	PM1,PM3	none	-	-
						CDH20	p.Gln371Arg	c.1112A>G	AR	homozygous	Both parents	None	US	PM1,BS1	none	-	-
						PLEC	p.Ala1545Val	c.4634C>T	CH	Heterozygous	Maternal	Benign	US	PM1,PM2	Limb-girdle muscular dystrophy, type 2Q.Epidermolysis bullosa simplex with muscular dystrophy	Unknown	[12]
Cardio-38.A	Poly Valvular disease, Mitral valve prolapse	None	11 years	M	Qatar	*LRRC56*	p.Pro305His	c.914C>A	Could not be identified	Heterozygous	Not reported *	None	US	-	none	-	-
cardio-40.A	Tricuspid Atresia, restrictive VSD,Large PDA	None	5 months	F	India	*DNAH9*	p.Arg1517Gln	c.4550G>A	CH	Heterozygous	Maternal	None	US	PM1	Crisponi/CISS1-like and Bohring-Opitz like syndrome	Turkish	[13]
						SAAL1	p.Pro95Leu	c.284C>T	CH	Heterozygous	Paternal	None	US	PM1	none	-	-
Cardio-41.A	Fontan,Right isomerism,Interrupted IVC, mitral atresis, DORV,Large VSD, severe PS	None	16 years	F	Yemen	*DYNC2H1*	p.Leu592Phe	c.1774C>T	AD	Heterozygous	Maternal	Uncertain significant	US	PM2,PP3	Intellectual disability, Jeune thoracic dystrophy, Short-rib thoracic dysplasia 3 with or without polydactyly	Unknown	[14]
Cardio-42.A	TOF	None	6 months	M	Kuwait	*DNAH5*	p.Asn1420Asp	c.4258A>G	CH	Heterozygous	Paternal	VUS	US	PM1,PM2	Primary ciliary dyskinesia	Unknown	[15]

Cardio-47.A	TGA	None	15 days	M	Egypt	*INPP5F*	p.Leu131Met	c.391C>A	CH	Heterozygous	Paternal	None	US	PM1,PM2	None	-	-
Cardio-48.A	HLH	None	20 days	F	Pakistan	*SHROOM3*	p.Gln1623Lys	c.4867C>A	AR	Homozygous	Both parents	None	US	PM1	None	-	-
						SCN10A	p.Ser470Tyr	c.1409C>A	CH	Heterozygous	Maternal	None	US	BP4, BP1, BS1, PP3	None	-	-
						PDE4DIP	p.Gly1542Arg	c.4624G>A	CH	Heterozygous	Paternal	Likely benign	US	PM2	None	-	-
Cardio-49.A	DILV, TGA	None	22 days	F	Pakistan	*NUP210*	p.Pro1742Arg	c.5225C>G	CH	Heterozygous	Maternal	None	US	PM2	None	-	-
						COL6A2	p.Arg784His	c.2351G>A	CH	Heterozygous	Maternal	Benign /likely benign	US	PM1,PM2,BP6	None	-	-
Cardio-50.A	TOF	None	4 months	M	India	*GLA*	p.Asp313Tyr	c.937G>T	UPD	Homozygous	Maternal	Conflicting interpretations of pathogenicity	US	BP6, PP3	Hypertrophic cardiomyopathy, Fabry disease, Angiokeratoma corporis diffusum,sudden unexplained death, cardiac variant	Unknown	[16]
Cardio-53.A	COA	None	3 months	M	Morocco	*KARS*	p.Glu120Gln	c.358G>C	CH	Heterozygous	Maternal	None	US	PM1,PM2	None	-	-
						PLEC	p.Gln2111His	c.6333G>C	CH	Heterozygous	Maternal	None	US	PM1,PM2,PP3	none	-	-
						ANKS6	p.Pro736Ala	c.2206C>G	CH	Heterozygous	Maternal	None	US	PM1,PM2,PP3,BP6	None	-	-

-Abbreviations:

TOF: tetralogy of Fallot, CHD: congenital cardiac defects.: HLH: hypoplastic left heart syndrome, ASD: atrial septal defect, ATSL arterial tortuosity syndrome, VSD: ventricular septal defect, PDA: patent ductus arteriousus,TAPVD: total anomalous pulmonary venous defect,DORV: double outlet right ventricle,TGA: transposition of great artery,DILV: double inlet left ventricle,COA: cortication of aorta F: female, M: Male, XLR: X-linked recessive, CH: compound heterozygous , AD: Autosomal dominant, AR: autosomal recessive,US: uncertain significance, UPD: uniparental disomy.

-ACMG/AMP sub-classification:

PM1: Located in a mutational hot spot and/or critical and well-established functional domain (*e.g.* active site of an enzyme) without benign variation

PM2: Absent from controls (or at extremely low frequency if recessive in Exome Sequencing Project, 1000 Genomes or ExAC

PM5: Novel missense change at an amino acid residue where a different missense change determined to be pathogenic has been seen before

BS1: Allele frequency is greater than expected for disorder

BP1: Missense variant in a gene for which primarily truncating variants are known to cause disease

BP4: Multiple lines of computational evidence suggest no impact on gene or gene product (conservation, evolutionary, splicing impact, etc)

BP6: Reputable source recently reports variant as benign but the evidence is not available to the laboratory to perform an independent evaluation

PVS1: Null variant (nonsense, frameshift, canonical +/−1 or 2 splice sites, initiation codon, single or multi-exon deletion) in a gene where loss of function (LOF) is a known mechanism of disease

PS2: *De novo* (both maternity and paternity confirmed) in a patient with the disease and no family history

PP2: Missense variant in a gene that has a low rate of benign missense variation and where missense variants are a common mechanism of disease

PP3: Multiple lines of computational evidence support a deleterious effect on the gene or gene product (conservation, evolutionary, splicing impact, etc)

PP5: Reputable source recently reports variant as pathogenic but the evidence is not available to the laboratory to perform an independent evaluation

*: number of enrolled family member is not enough to identify the source of the variant.

**: father was not enrolled, could not identify the source of the other variant copy.

***: Mother was not enrolled, could not identify the source of the other variant copy.

References

1. Savarese, Marco, Lorenzo Maggi, Anna Vihola, Per Harald Jonson, Giorgio Tasca, Lucia Ruggiero, Luca Bello, Francesca Magri, Teresa Giugliano, and Annalaura Torella. "Interpreting Genetic Variants in Titin in Patients with Muscle Disorders." *JAMA Neurol.* 75, no. 5 (2018): 557-65.
2. Faiyaz-Ul-Haque, M, SHE Zaidi, AA Wahab, A Eltohami, MS Al-Mureikhi, G Al-Thani, VD Peltekova, L-C Tsui, and AS Teebi. "Identification of a P. Ser81arg Encoding Mutation in Slc2a10 Gene of Arterial Tortuosity Syndrome Patients from 10 Qatari Families." *Clin. Genet.* 74, no. 2 (2008): 189-93.
3. Faiyaz-Ul-Haque, Muhammad, Syed HE Zaidi, Nouriyah Al-Sanna, Abdulrahman Alswaid, Tariq Momenah, Namik Kaya, Fouad Al-Dayel, Issam Bouhoaigah, Mohammed Saliem, and Lap-Chee Tsui. "A Novel Missense and a Recurrent Mutation in Slc2a10 Gene of Patients Affected with Arterial Tortuosity Syndrome." *Atherosclerosis* 203, no. 2 (2009): 466-71.
4. [VCV000773847.2], National Center for Biotechnology Information. https://www.ncbi.nlm.nih.gov/clinvar/variation/VCV000773847.2 (
5. National Center for Biotechnology Information. . "[Vcv000167010.9]." ClinVar, https://www.ncbi.nlm.nih.gov/clinvar/variation/VCV000167010.9 (
6. [VCV000722668.2], National Center for Biotechnology Information. ClinVar, https://www.ncbi.nlm.nih.gov/clinvar/variation/VCV000722668.2 (
7. Information, National Center for Biotechnology. "Clinvar; [Vcv000722667.2]." https://www.ncbi.nlm.nih.gov/clinvar/variation/VCV000722667.2 (
8. Zhu, Tianwen, Xiaohui Gong, Fei Bei, Li Ma, Jingjing Sun, Jian Wang, Gang Qiu, Jianhua Sun, Yu Sun, and Yongjun Zhang. "Primary Immunodeficiency-Related Genes in Neonatal Intensive Care Unit Patients with Various Genetic Immune Abnormalities: A Multicentre Study in China." *Clin. Transl. Immunol* 10, no. 3 (2021): e1266.
9. Sun, Yifan, Jingjing Sun, Na Li, Cheng Cai, Xiaohui Gong, and Li Ma. "Phenotypic Spectrum of Typical Charge Syndrome in a Chinese Male Neonate: A Case Report." *Transl. Pediatr.* 9, no. 2 (2020): 180.
10. National Center for Biotechnology Information, [VCV000136948.9]. "Clinvar." https://www.ncbi.nlm.nih.gov/clinvar/variation/VCV000136948.9 (
11. [VCV000707217.7], National Center for Biotechnology Information. https://www.ncbi.nlm.nih.gov/clinvar/variation/VCV000707217.7 (
12. [VCV000129948.9], National Center for Biotechnology Information. "National Center for Biotechnology Information. [Vcv000129948.9]." ClinVar, https://www.ncbi.nlm.nih.gov/clinvar/variation/VCV000129948.9 (
13. Kanthi, Anil, Malavika Hebbar, Stephanie L Bielas, Katta M Girisha, and Anju Shukla. "Bi-Allelic C. 181_183deltgt in Btb Domain of Klhl7 Is Associated with Overlapping Phenotypes of Crisponi/Ciss1-Like and Bohring-Opitz Like Syndrome." *Eur. J. Med. Genet.* 62, no. 6 (2019): 103528.
14. [VCV000216489.13], National Center for Biotechnology Information. https://www.ncbi.nlm.nih.gov/clinvar/variation/VCV000216489.13 (
15. National Center for Biotechnology Information, [VCV000258032.9]. "National Center for Biotechnology Information,[Vcv000258032.9]." https://www.ncbi.nlm.nih.gov/clinvar/variation/VCV000258032.9 (
16. [VCV000010738.32], National Center for Biotechnology Information. "National Center for Biotechnology Information. [Vcv000010738.32]." ClinVar, https://www.ncbi.nlm.nih.gov/clinvar/variation/VCV000010738.32 (
