# Supplementary figures and images for: Investigation of Genetic Causes in Patients with Congenital Heart Disease in Qatar: Findings from the Sidra Cardiac Registry
Source: Genes (Basel). 2022 Jul 30;13(8):1369. doi: 10.3390/genes13081369 (PMC9407366; doi:10.3390/genes13081369)

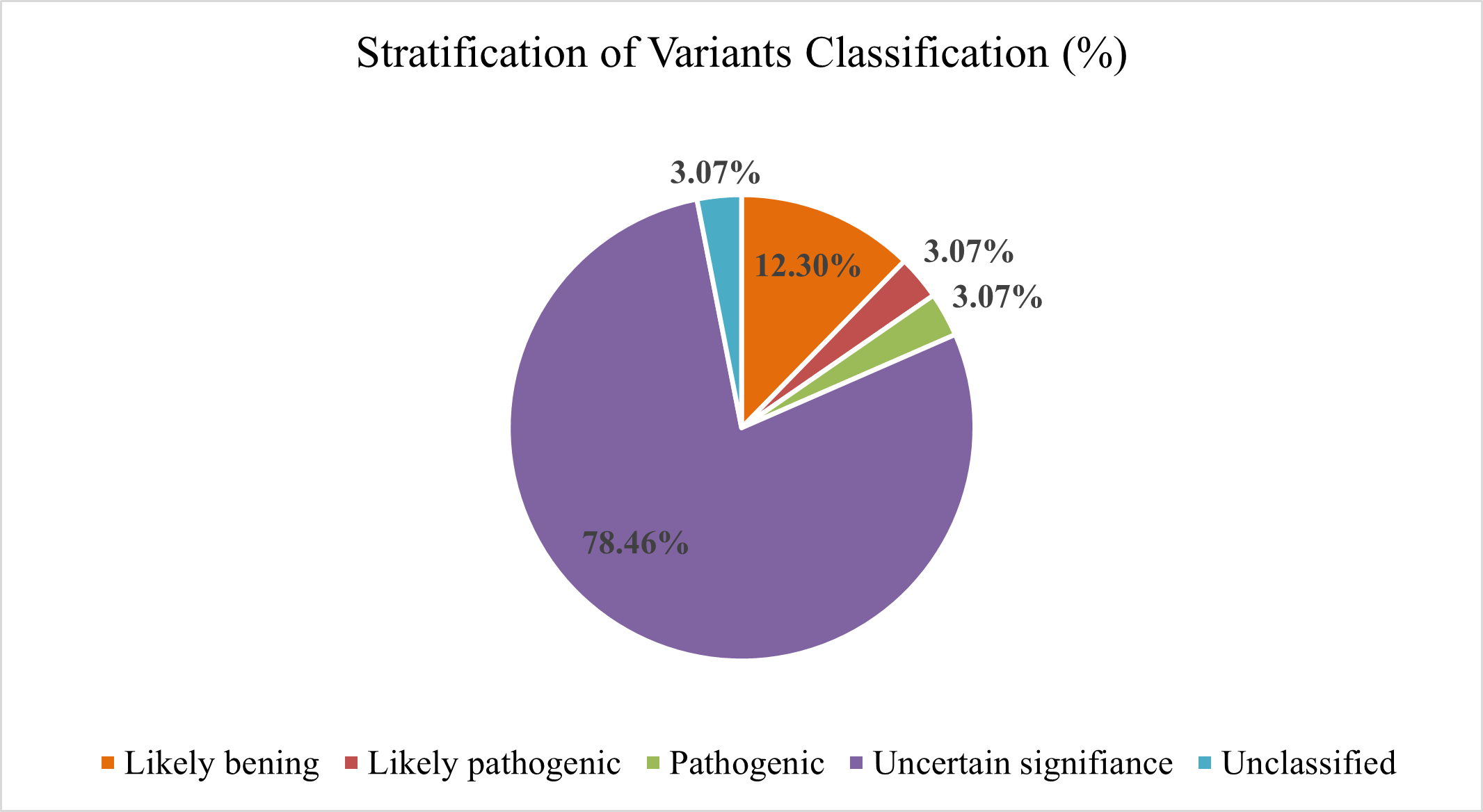

Supplement: Supplementary file 1 [file genes-13-01369-s001.zip › supplementary figures/Figure S1.png]

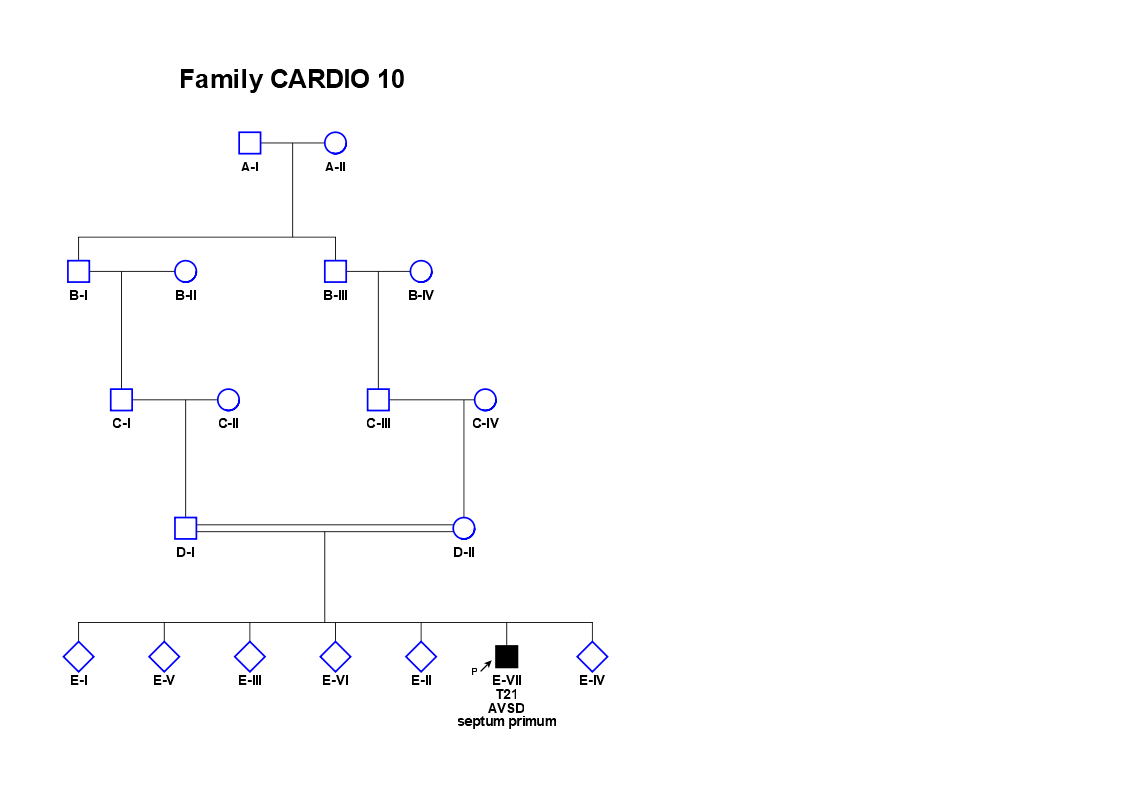

Supplement: Supplementary file 1 [file genes-13-01369-s001.zip › supplementary figures/pedigree/family-CARDIO 10.png]

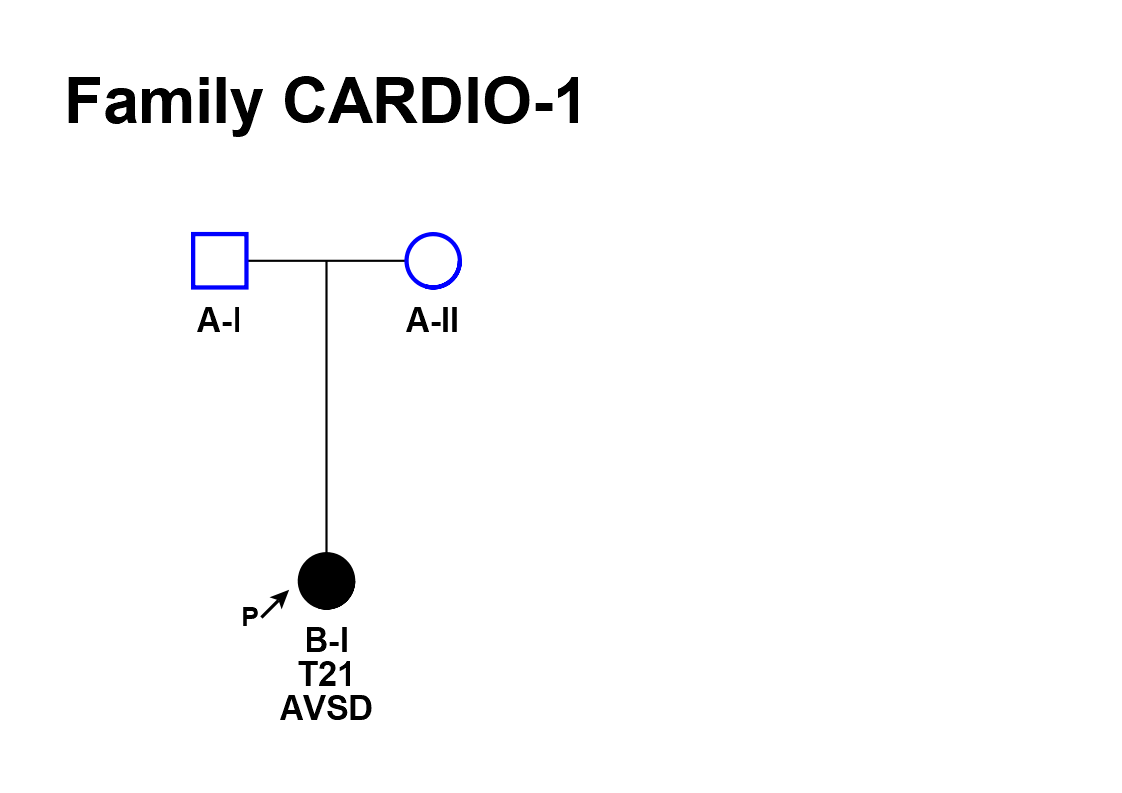

Supplement: Supplementary file 1 [file genes-13-01369-s001.zip › supplementary figures/pedigree/family-CARDIO-1.png]

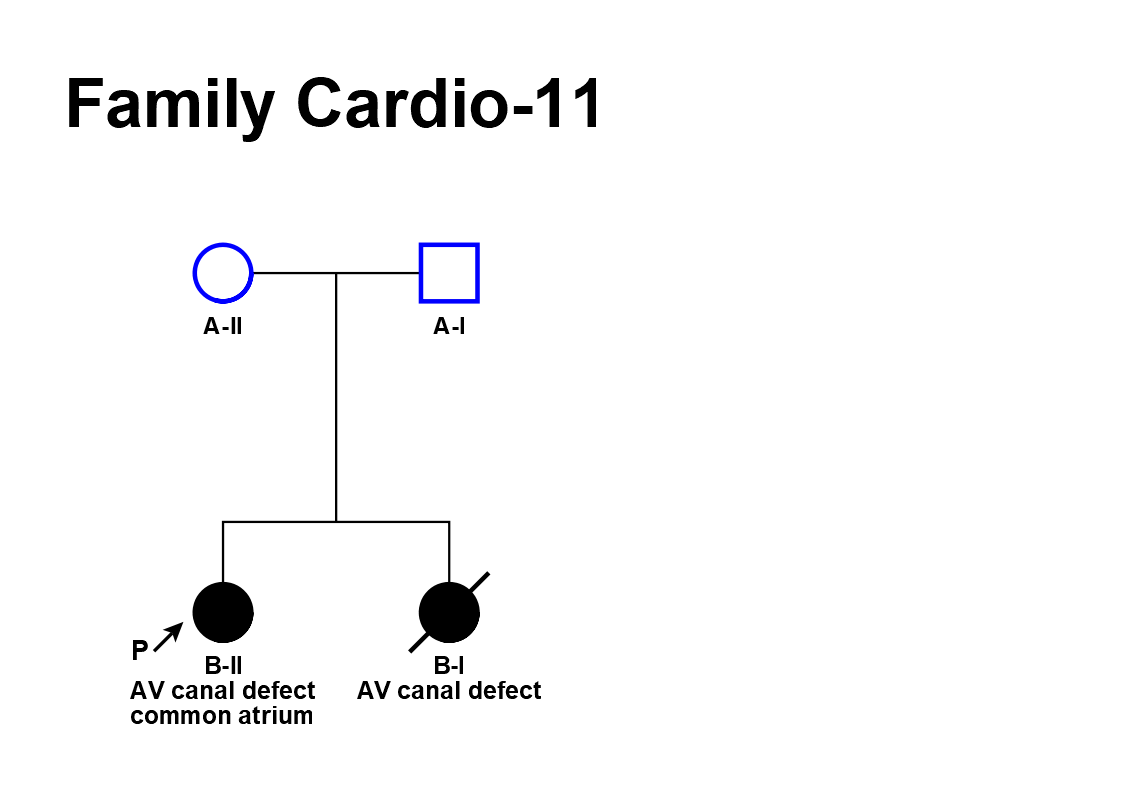

Supplement: Supplementary file 1 [file genes-13-01369-s001.zip › supplementary figures/pedigree/family-Cardio-11.png]

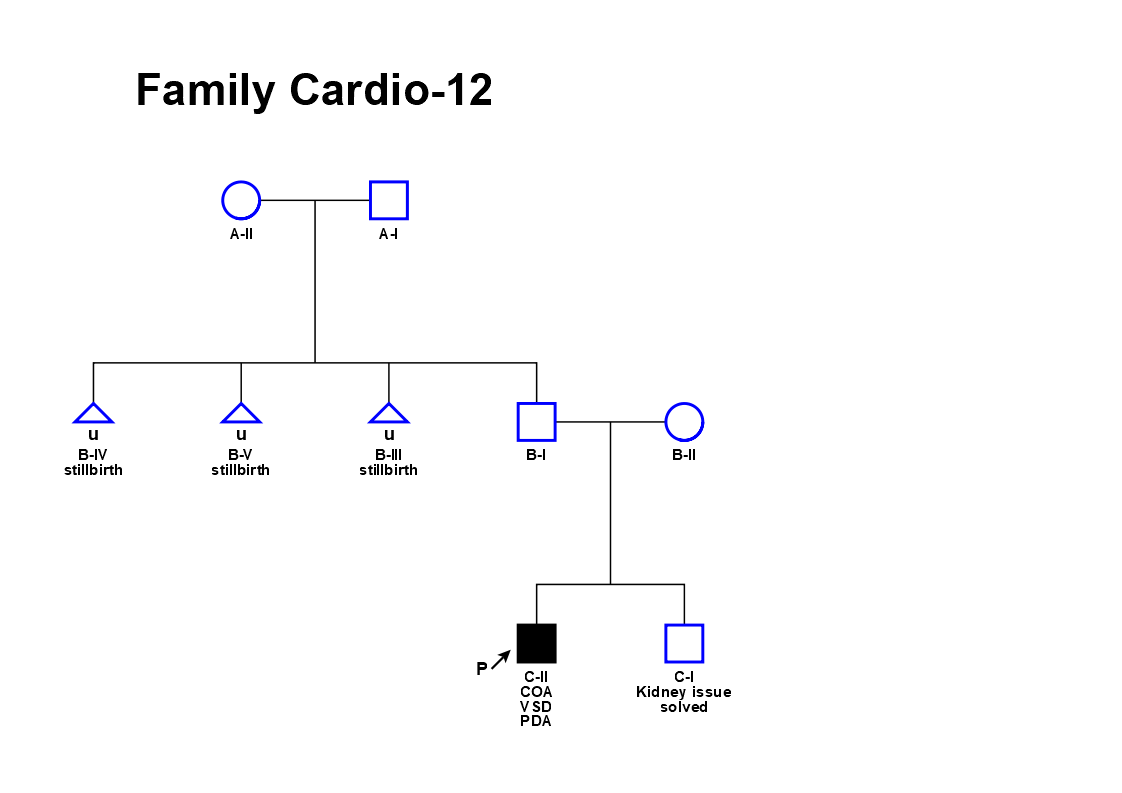

Supplement: Supplementary file 1 [file genes-13-01369-s001.zip › supplementary figures/pedigree/family-Cardio-12.png]

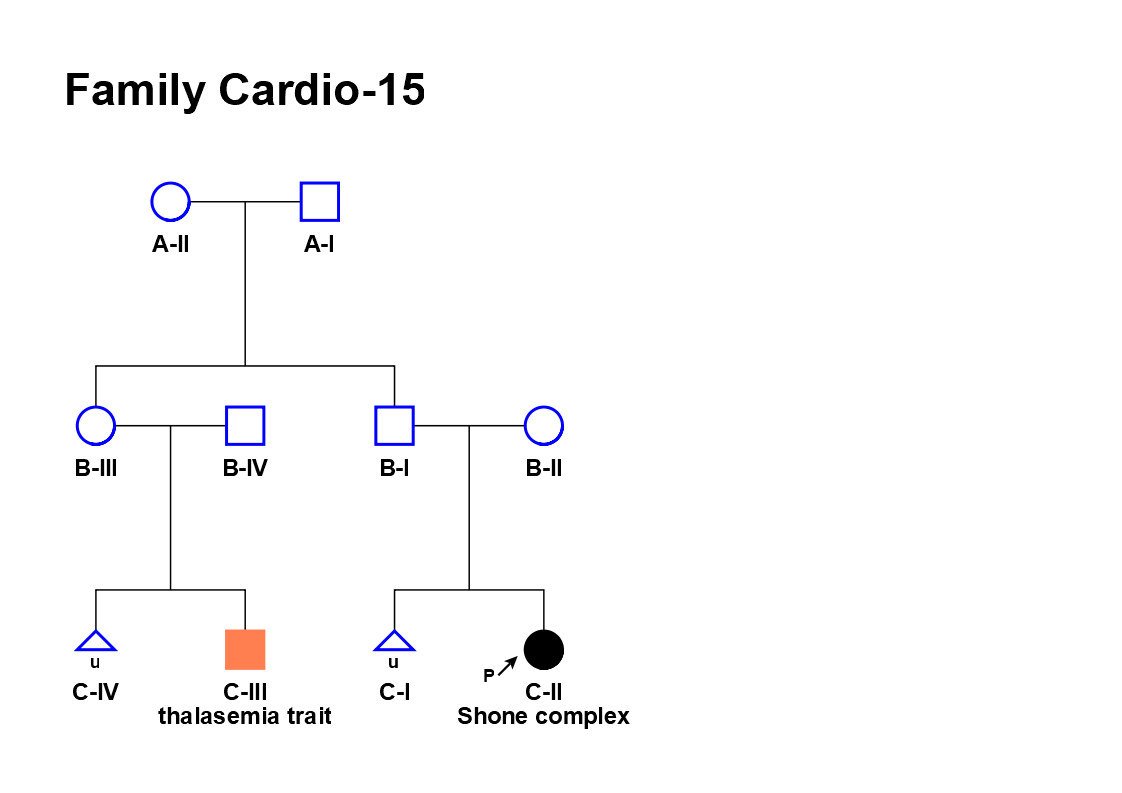

Supplement: Supplementary file 1 [file genes-13-01369-s001.zip › supplementary figures/pedigree/family-Cardio-15.png]

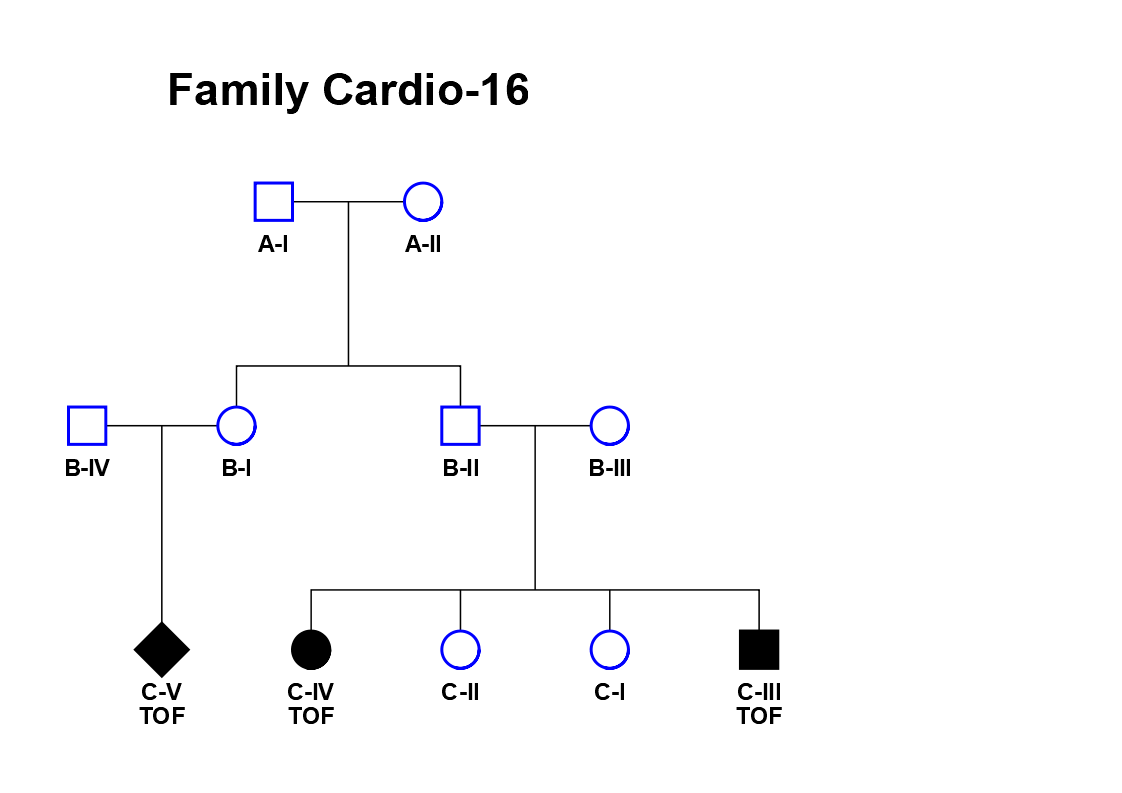

Supplement: Supplementary file 1 [file genes-13-01369-s001.zip › supplementary figures/pedigree/family-Cardio-16.png]

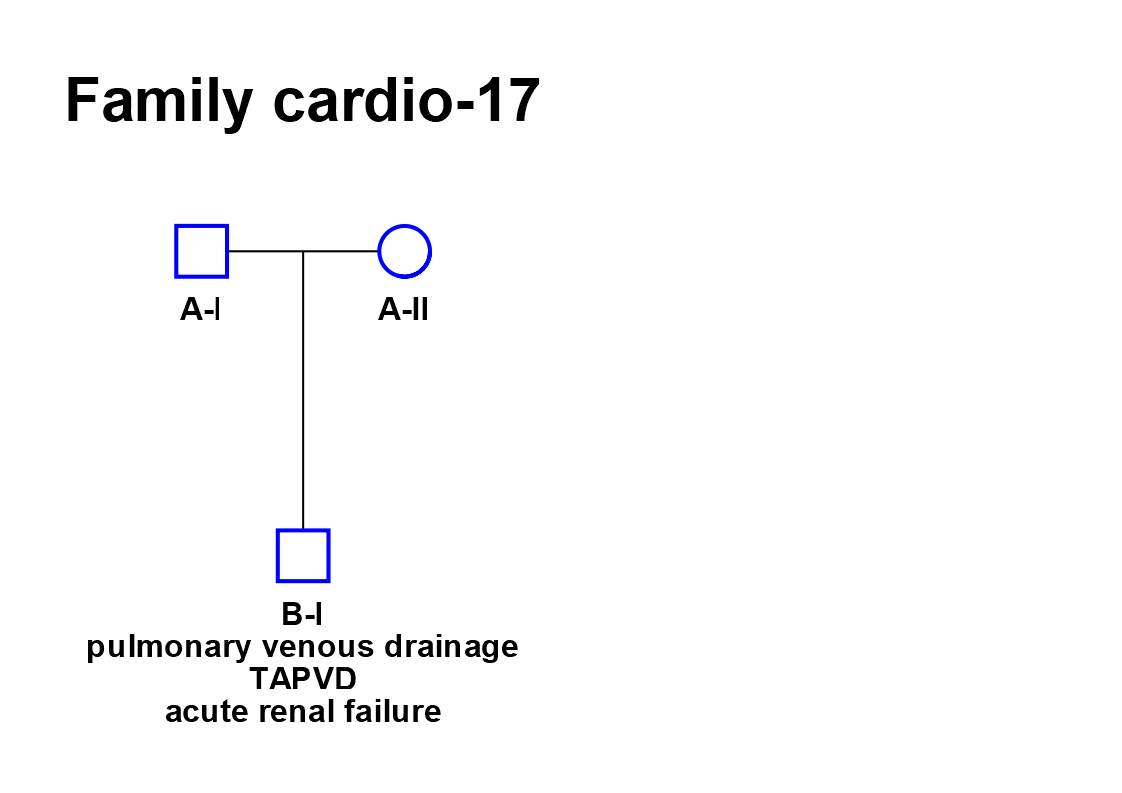

Supplement: Supplementary file 1 [file genes-13-01369-s001.zip › supplementary figures/pedigree/family-cardio-17.png]

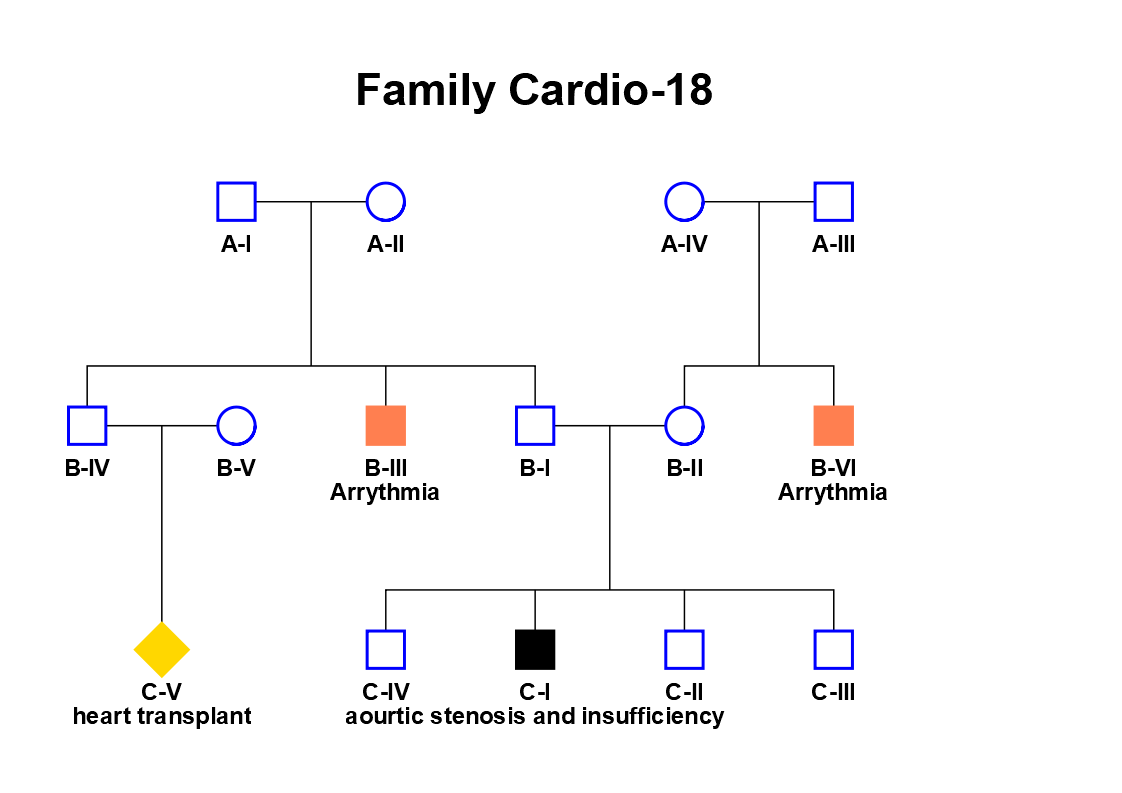

Supplement: Supplementary file 1 [file genes-13-01369-s001.zip › supplementary figures/pedigree/family-Cardio-18.png]

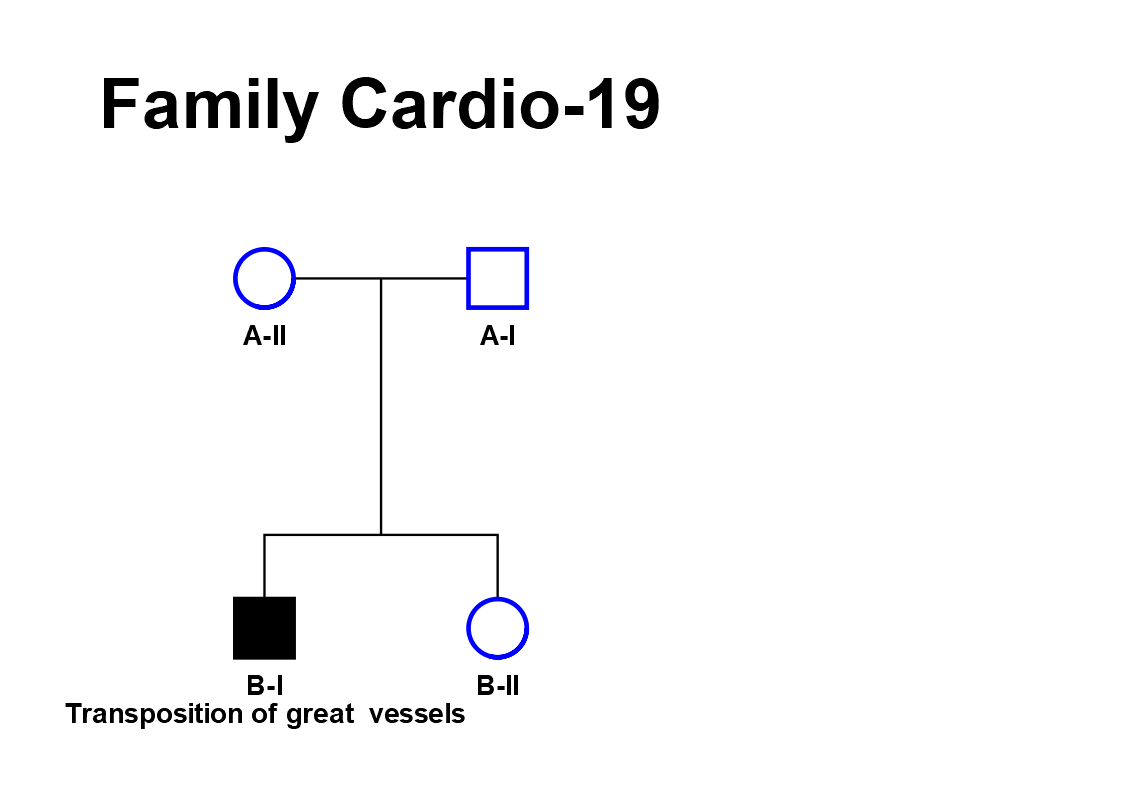

Supplement: Supplementary file 1 [file genes-13-01369-s001.zip › supplementary figures/pedigree/family-Cardio-19.png]

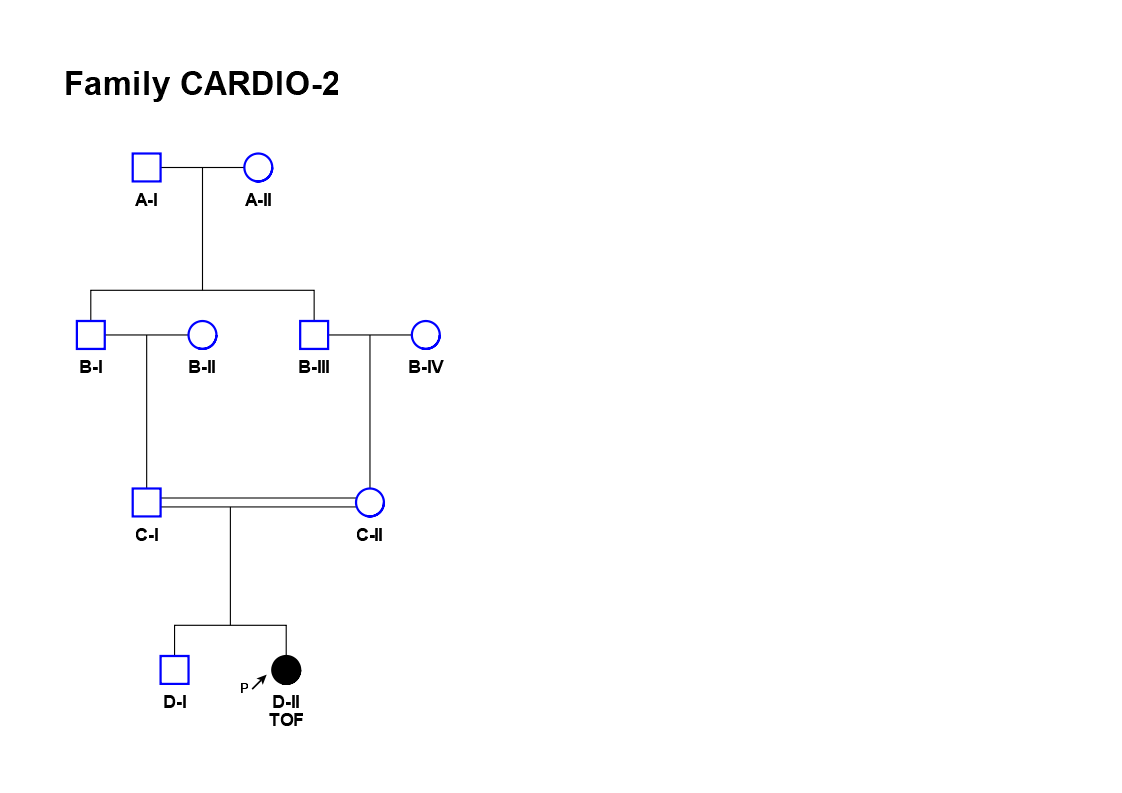

Supplement: Supplementary file 1 [file genes-13-01369-s001.zip › supplementary figures/pedigree/family-CARDIO-2.png]

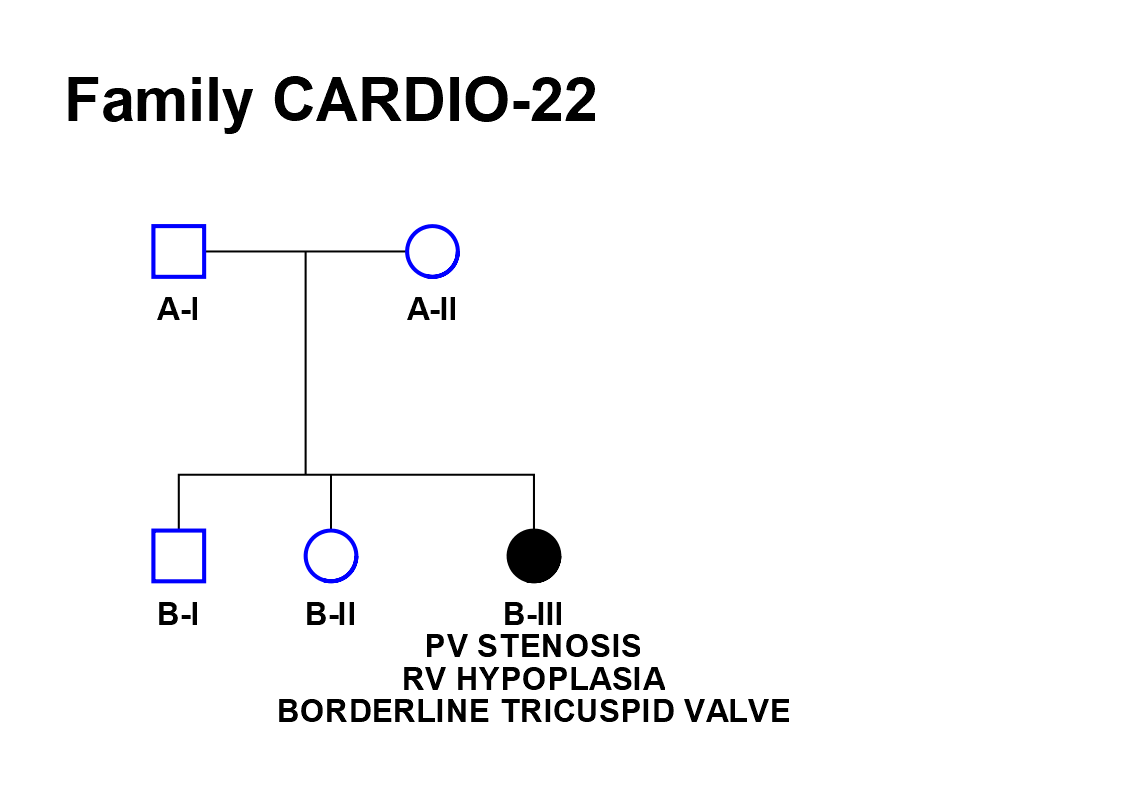

Supplement: Supplementary file 1 [file genes-13-01369-s001.zip › supplementary figures/pedigree/family-CARDIO-22.png]

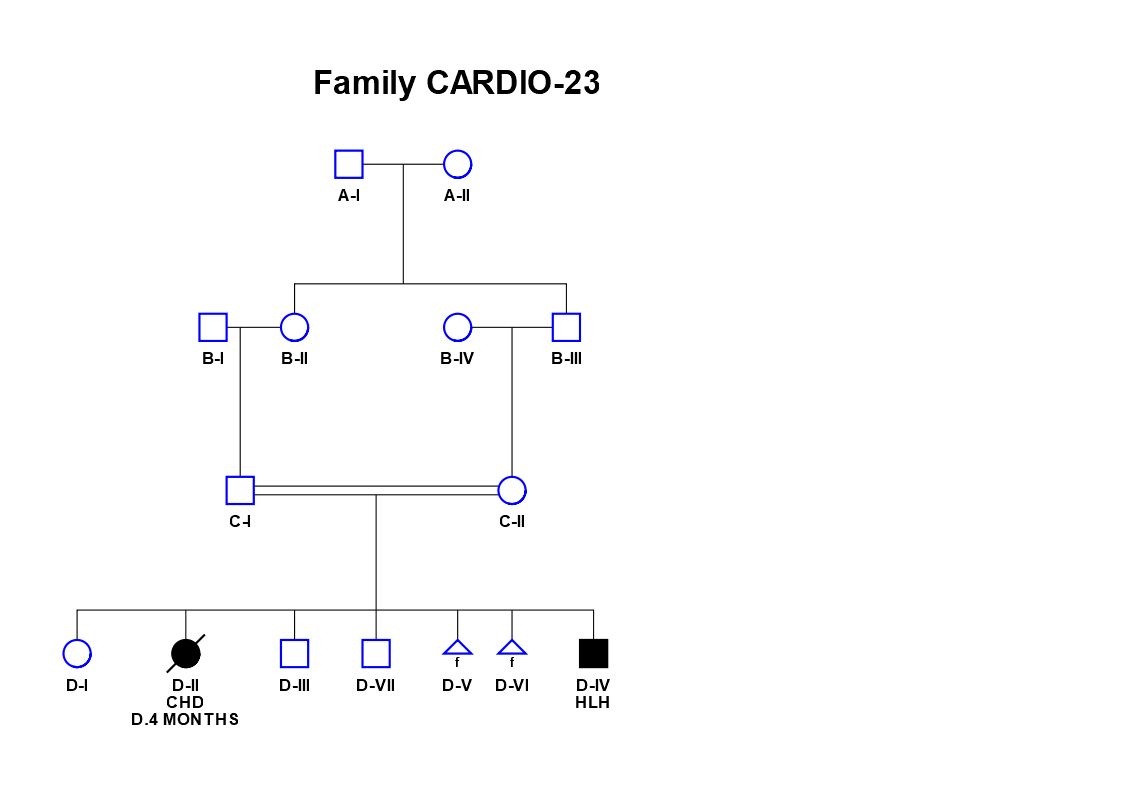

Supplement: Supplementary file 1 [file genes-13-01369-s001.zip › supplementary figures/pedigree/family-CARDIO-23.png]

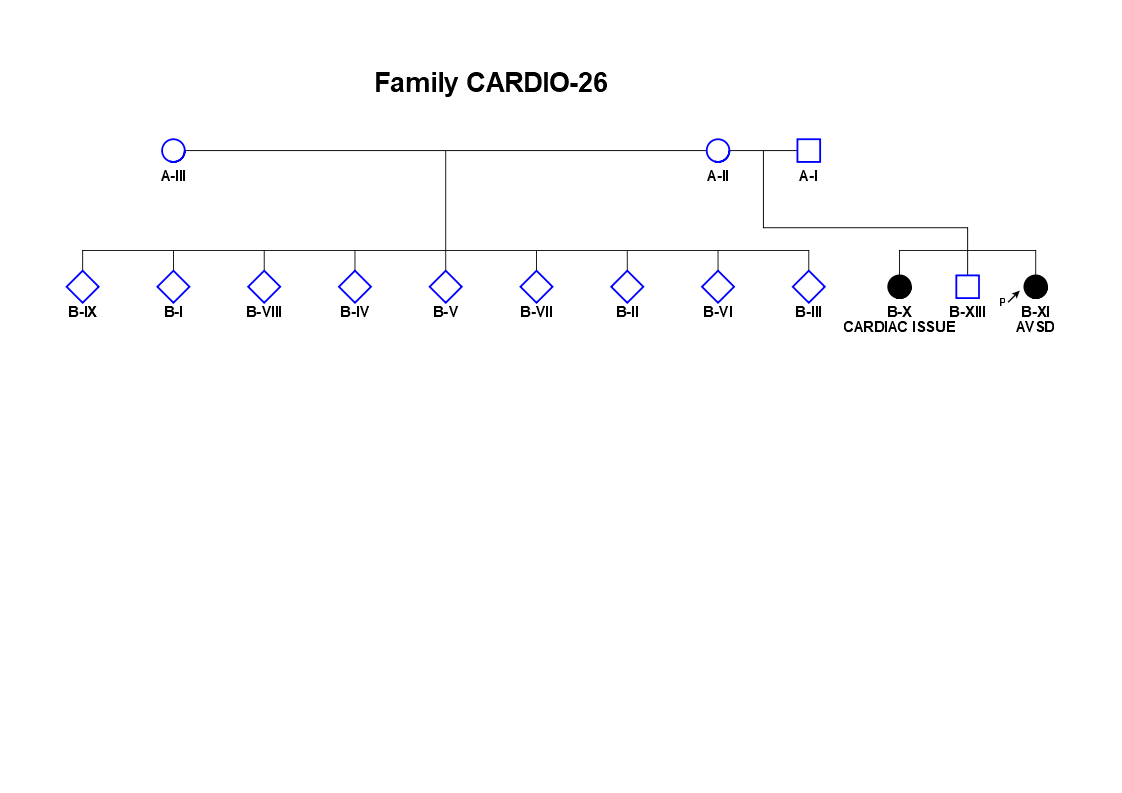

Supplement: Supplementary file 1 [file genes-13-01369-s001.zip › supplementary figures/pedigree/family-CARDIO-26.png]

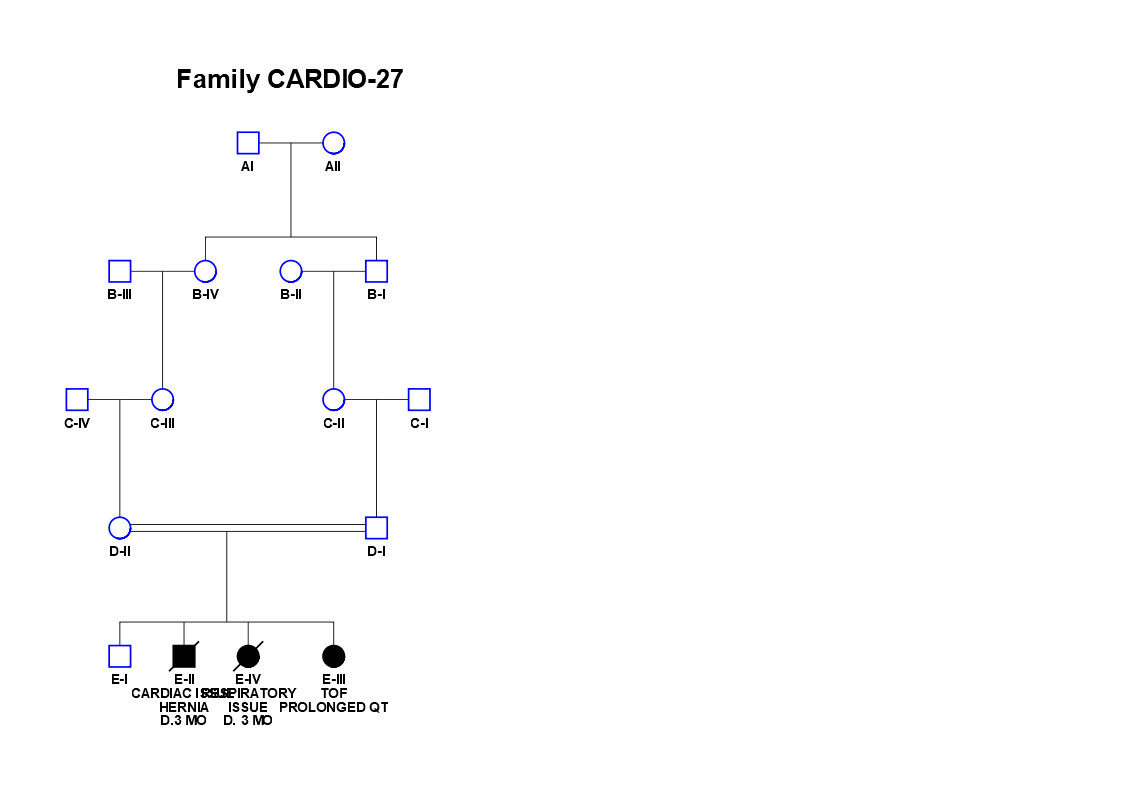

Supplement: Supplementary file 1 [file genes-13-01369-s001.zip › supplementary figures/pedigree/family-CARDIO-27.png]

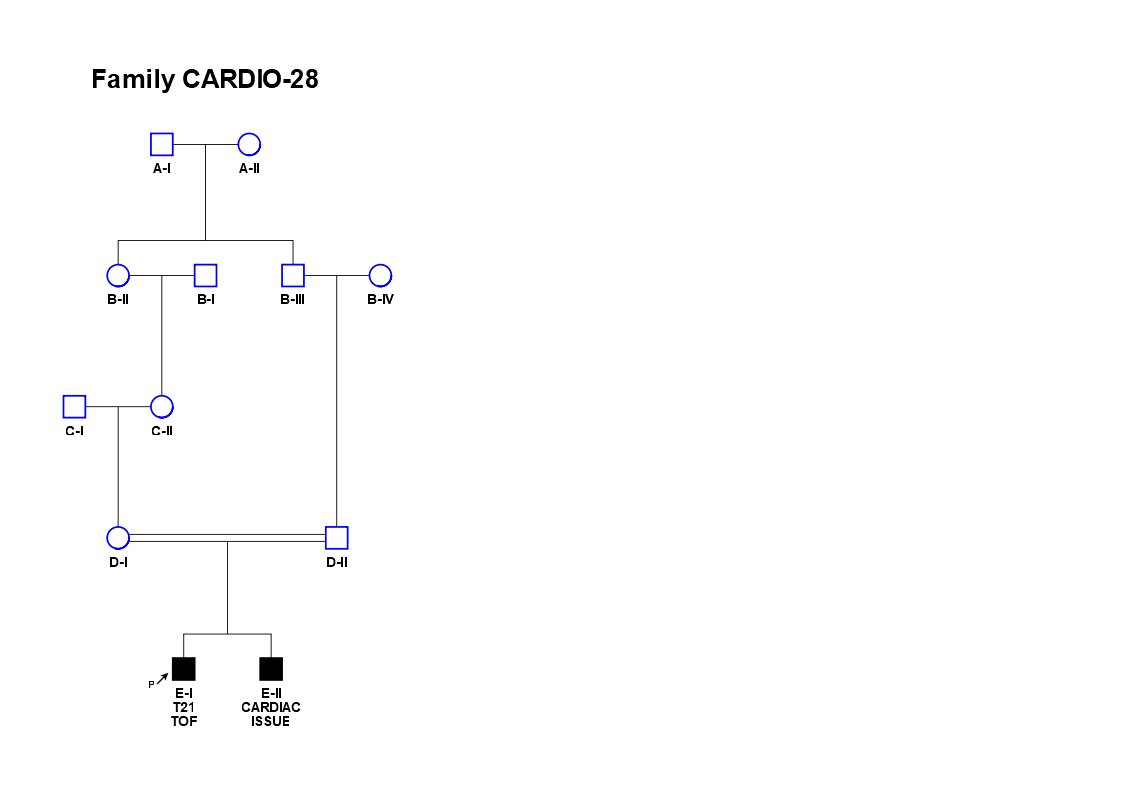

Supplement: Supplementary file 1 [file genes-13-01369-s001.zip › supplementary figures/pedigree/family-CARDIO-28.png]

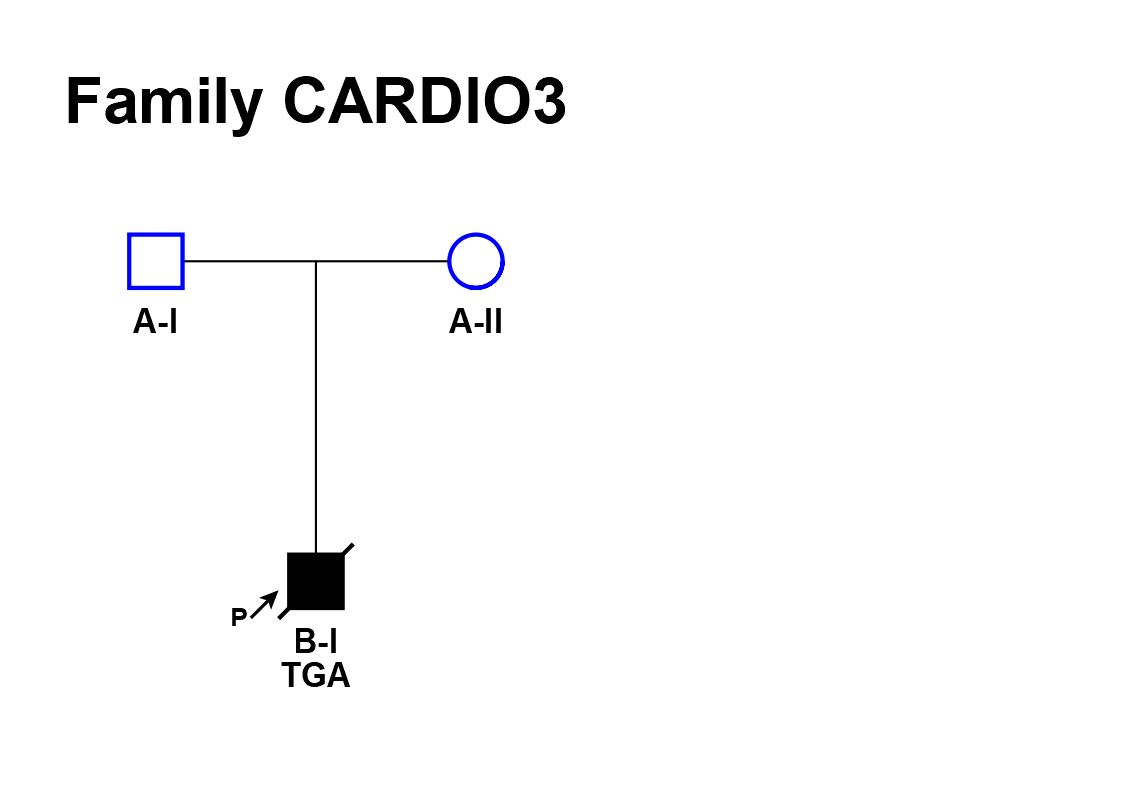

Supplement: Supplementary file 1 [file genes-13-01369-s001.zip › supplementary figures/pedigree/family-CARDIO3.png]

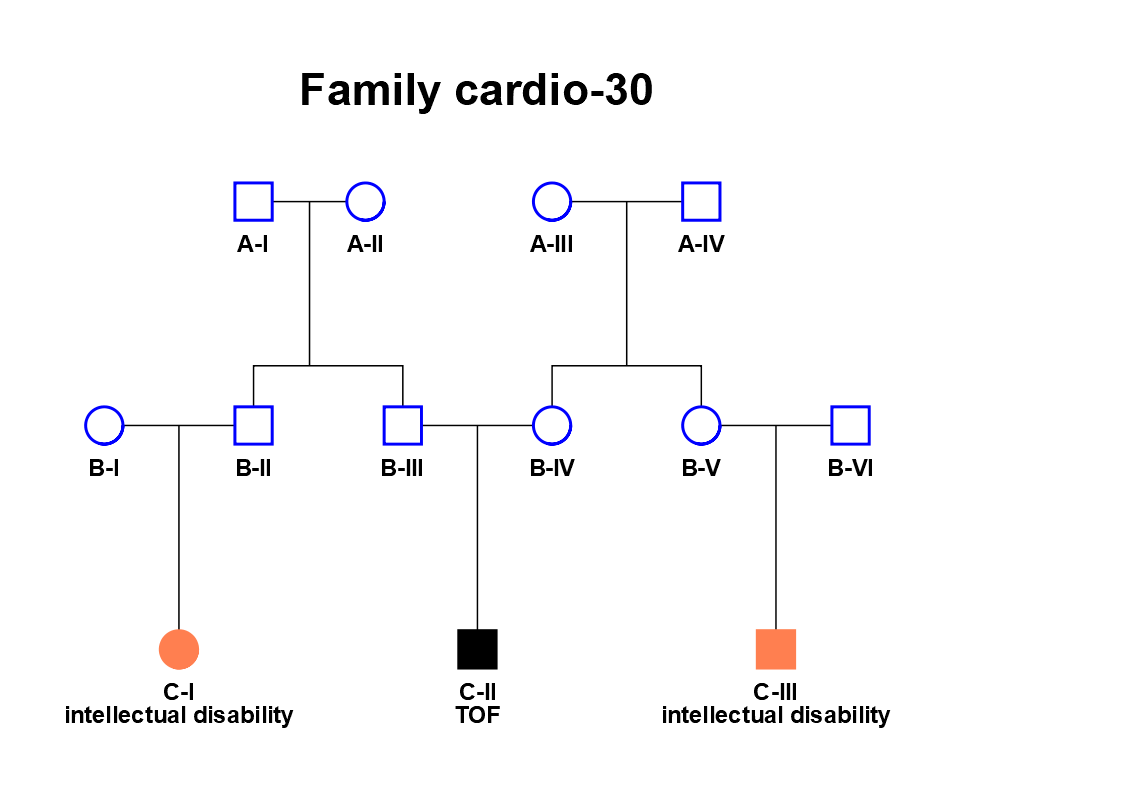

Supplement: Supplementary file 1 [file genes-13-01369-s001.zip › supplementary figures/pedigree/family-cardio-30.png]

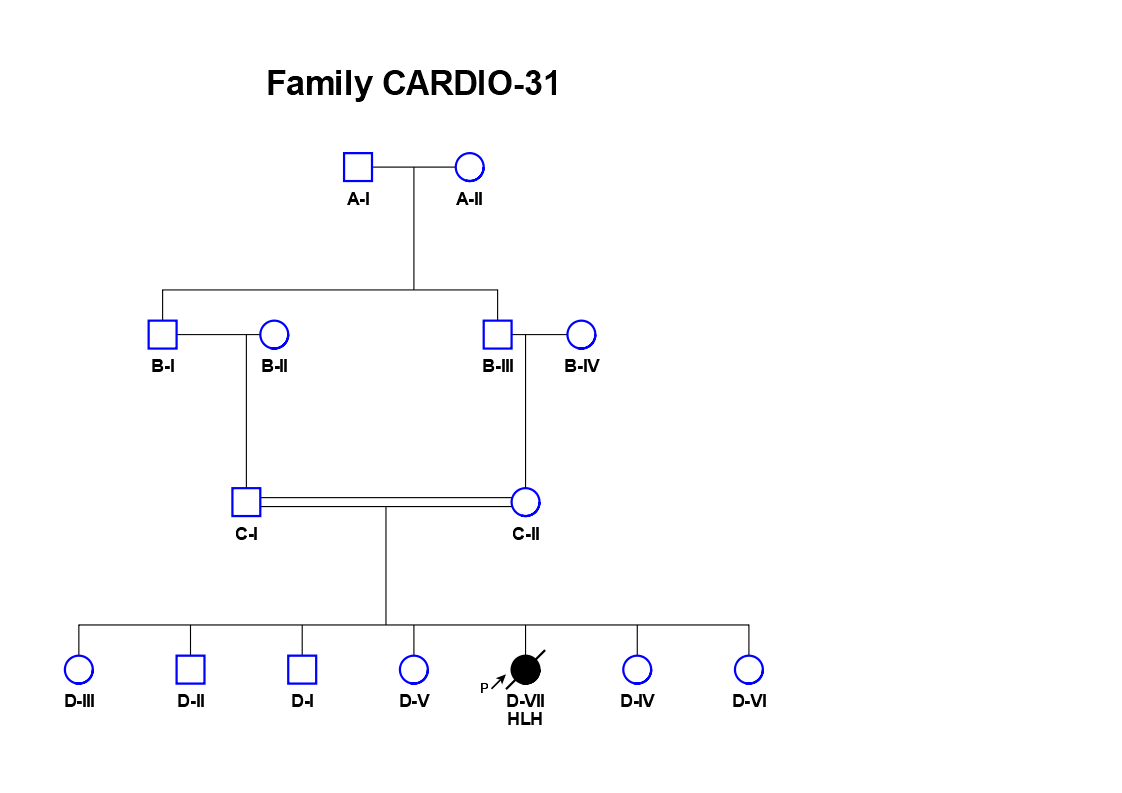

Supplement: Supplementary file 1 [file genes-13-01369-s001.zip › supplementary figures/pedigree/family-CARDIO-31.png]

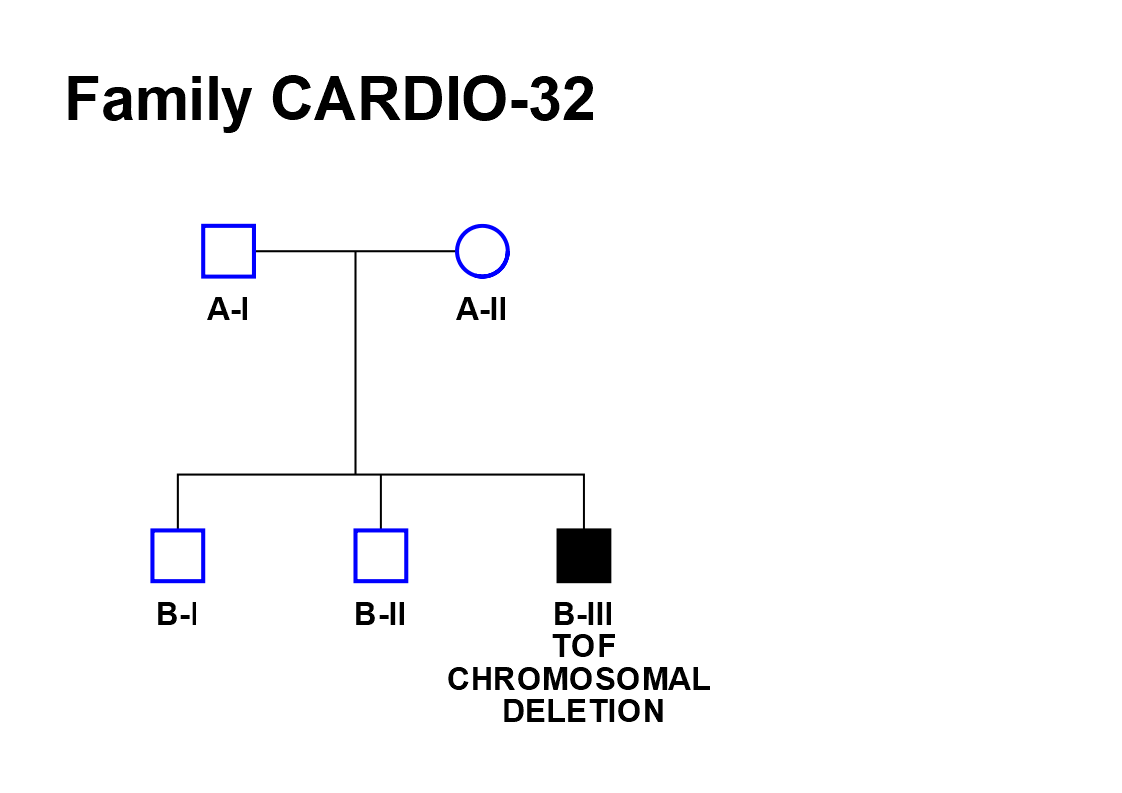

Supplement: Supplementary file 1 [file genes-13-01369-s001.zip › supplementary figures/pedigree/family-CARDIO-32.png]

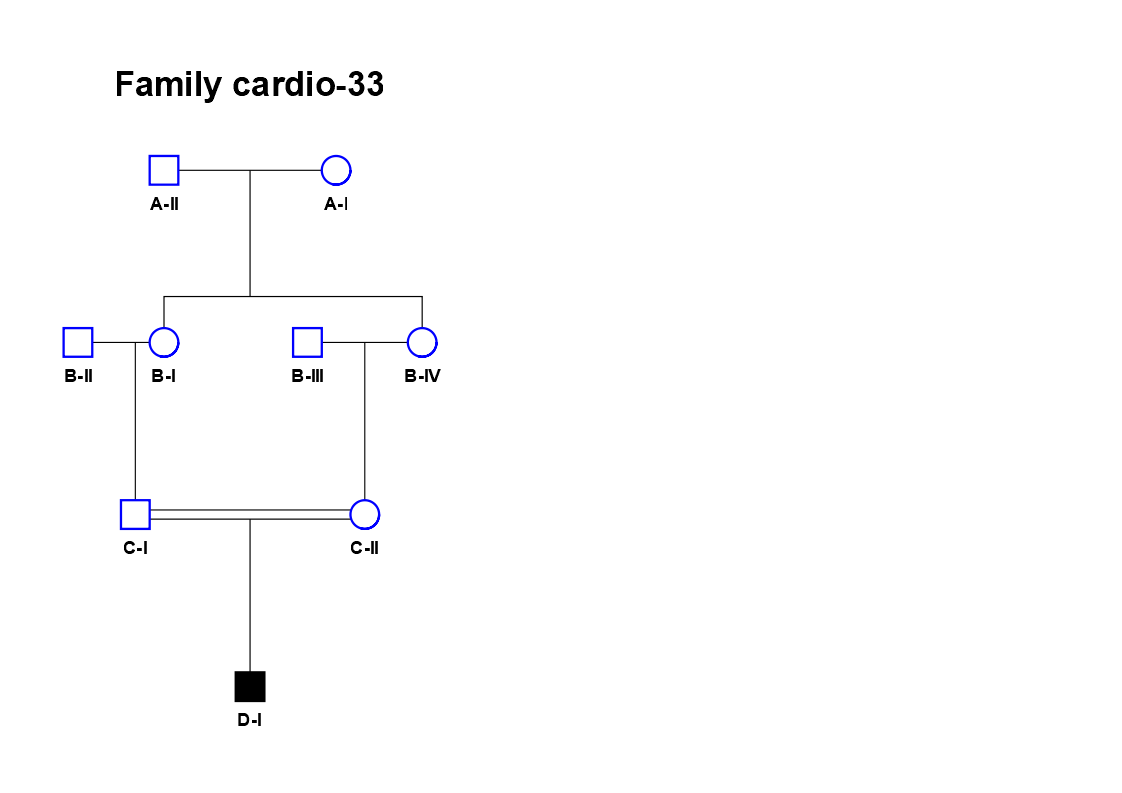

Supplement: Supplementary file 1 [file genes-13-01369-s001.zip › supplementary figures/pedigree/family-cardio-33.png]

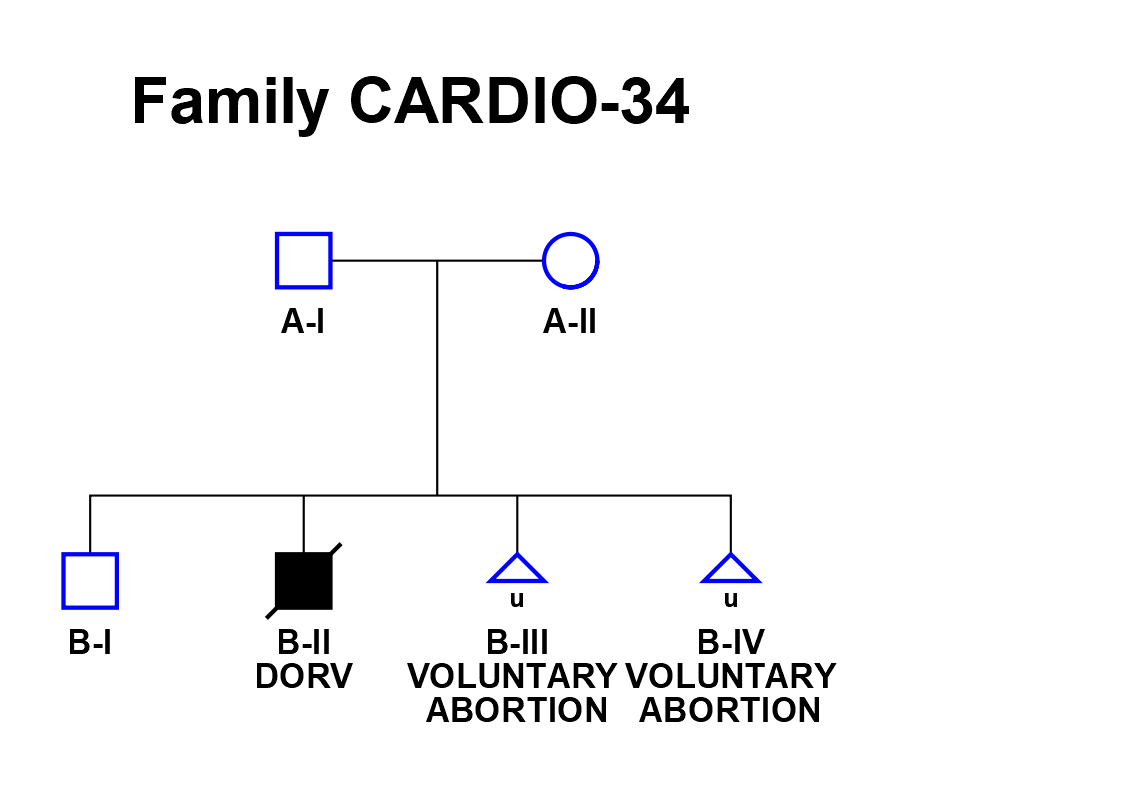

Supplement: Supplementary file 1 [file genes-13-01369-s001.zip › supplementary figures/pedigree/family-CARDIO-34.png]

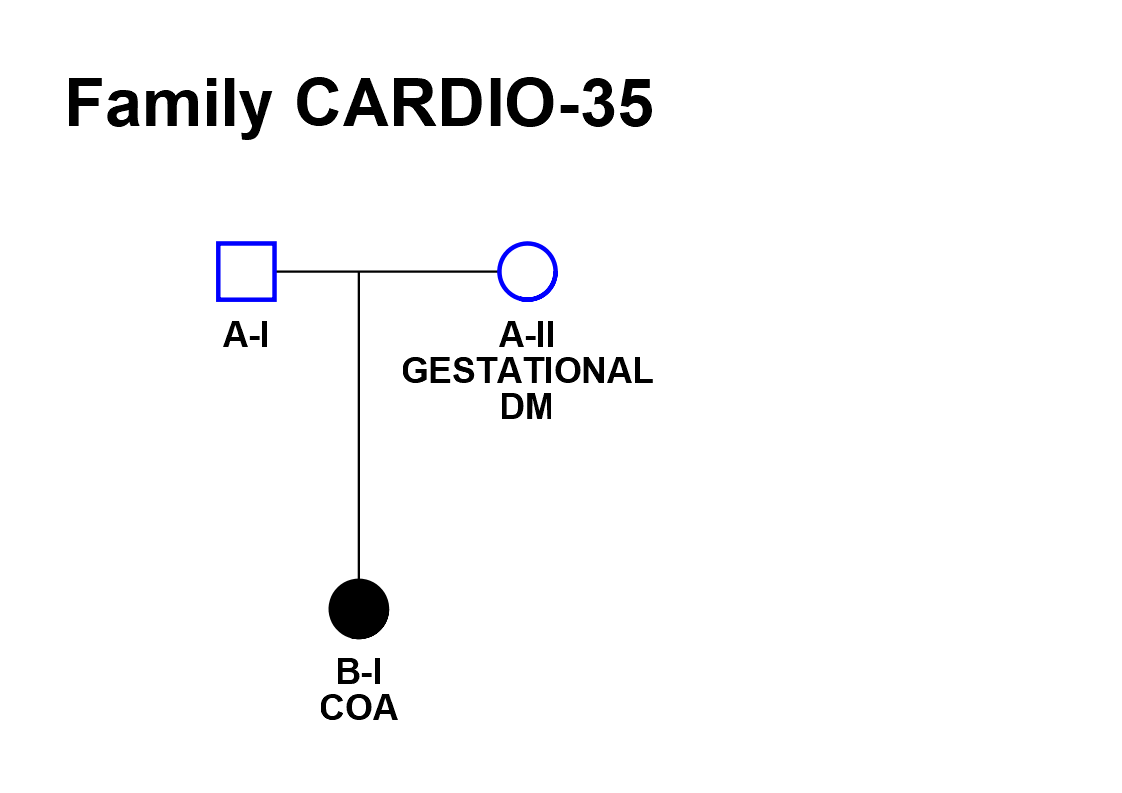

Supplement: Supplementary file 1 [file genes-13-01369-s001.zip › supplementary figures/pedigree/family-CARDIO-35.png]

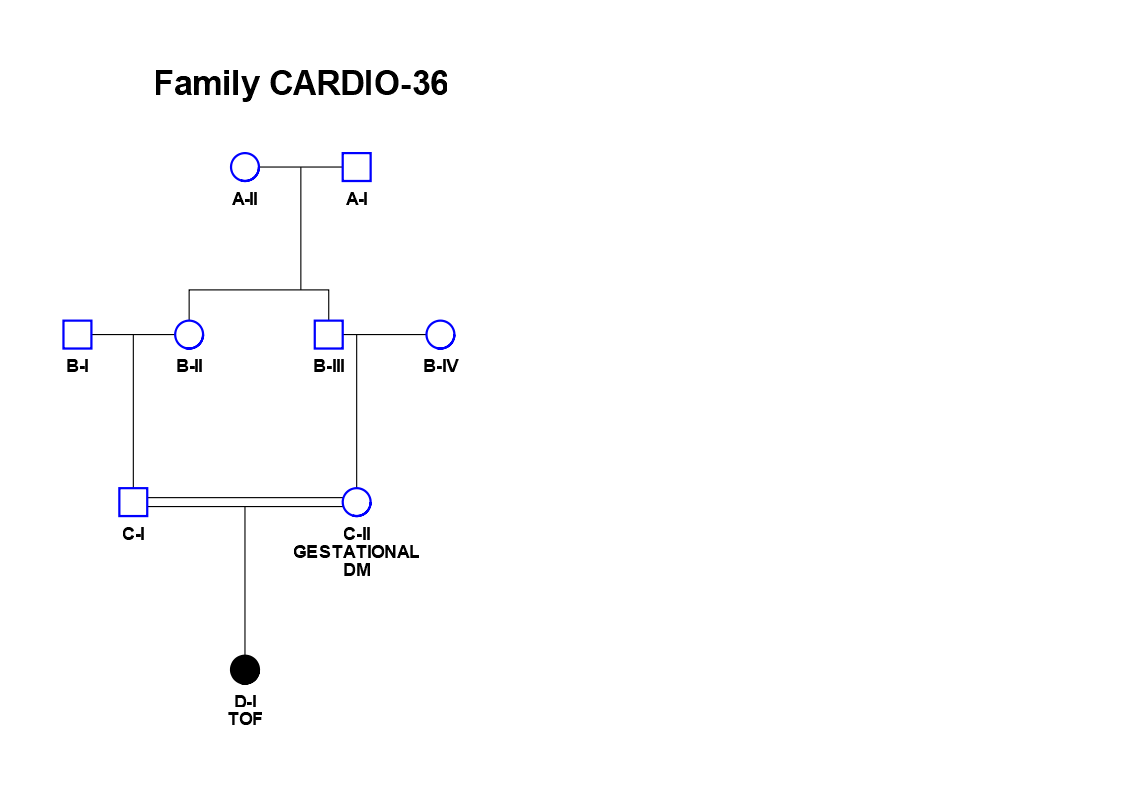

Supplement: Supplementary file 1 [file genes-13-01369-s001.zip › supplementary figures/pedigree/family-CARDIO-36.png]

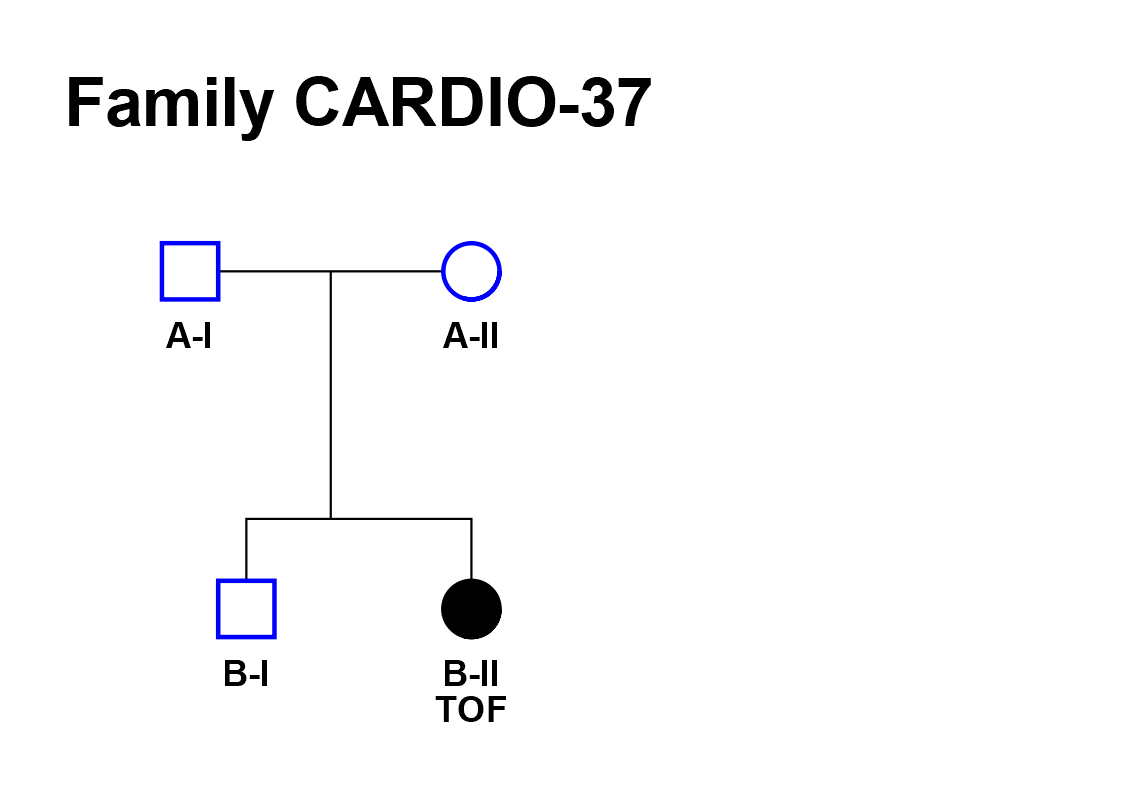

Supplement: Supplementary file 1 [file genes-13-01369-s001.zip › supplementary figures/pedigree/family-CARDIO-37.png]

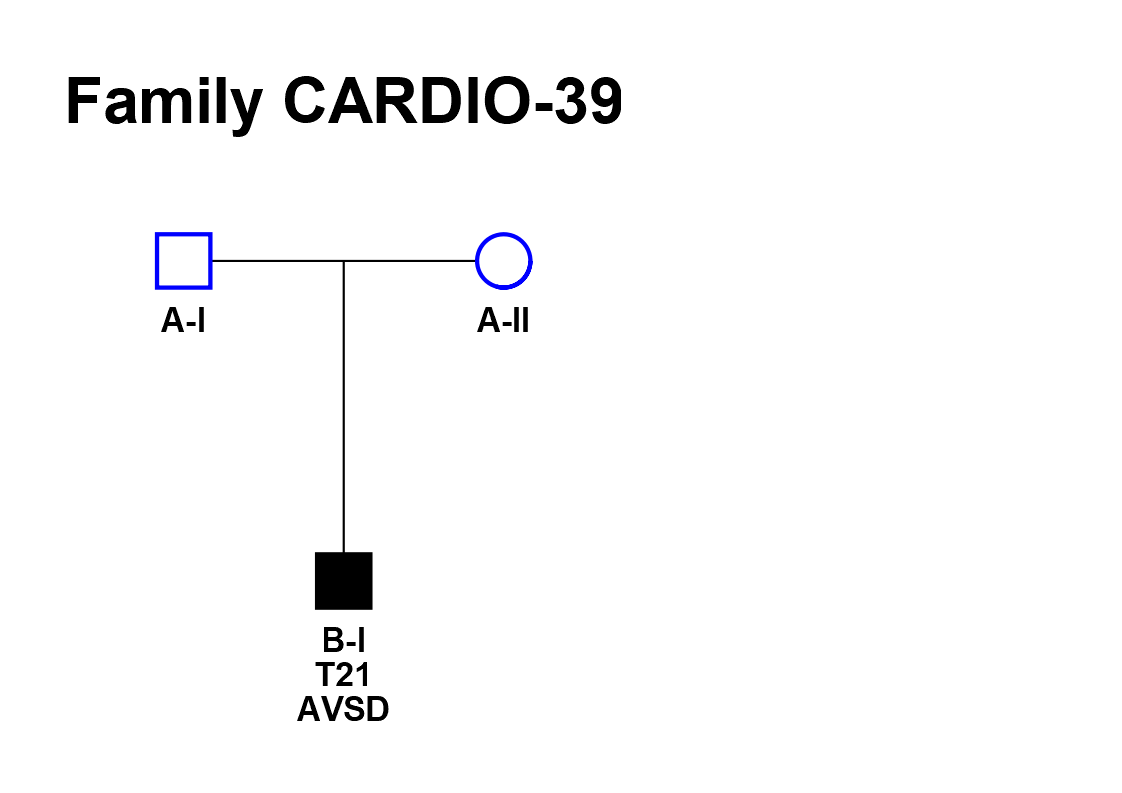

Supplement: Supplementary file 1 [file genes-13-01369-s001.zip › supplementary figures/pedigree/family-CARDIO-39.png]

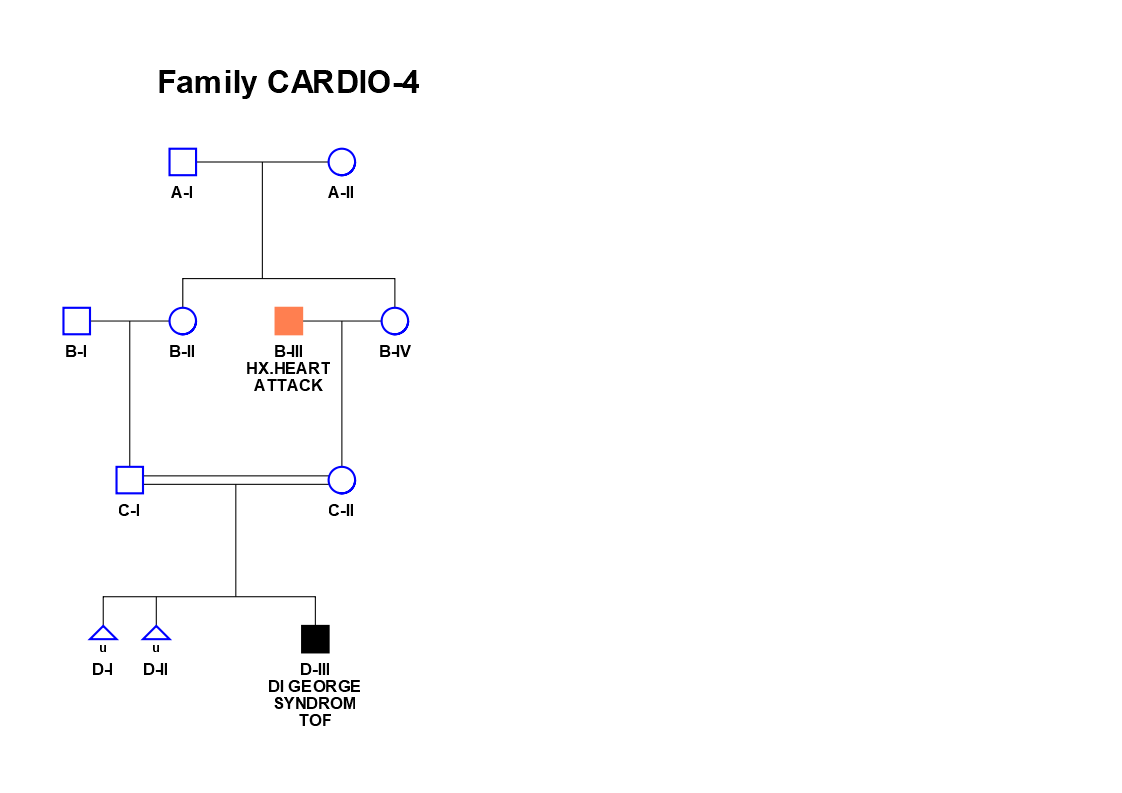

Supplement: Supplementary file 1 [file genes-13-01369-s001.zip › supplementary figures/pedigree/family-CARDIO-4.png]

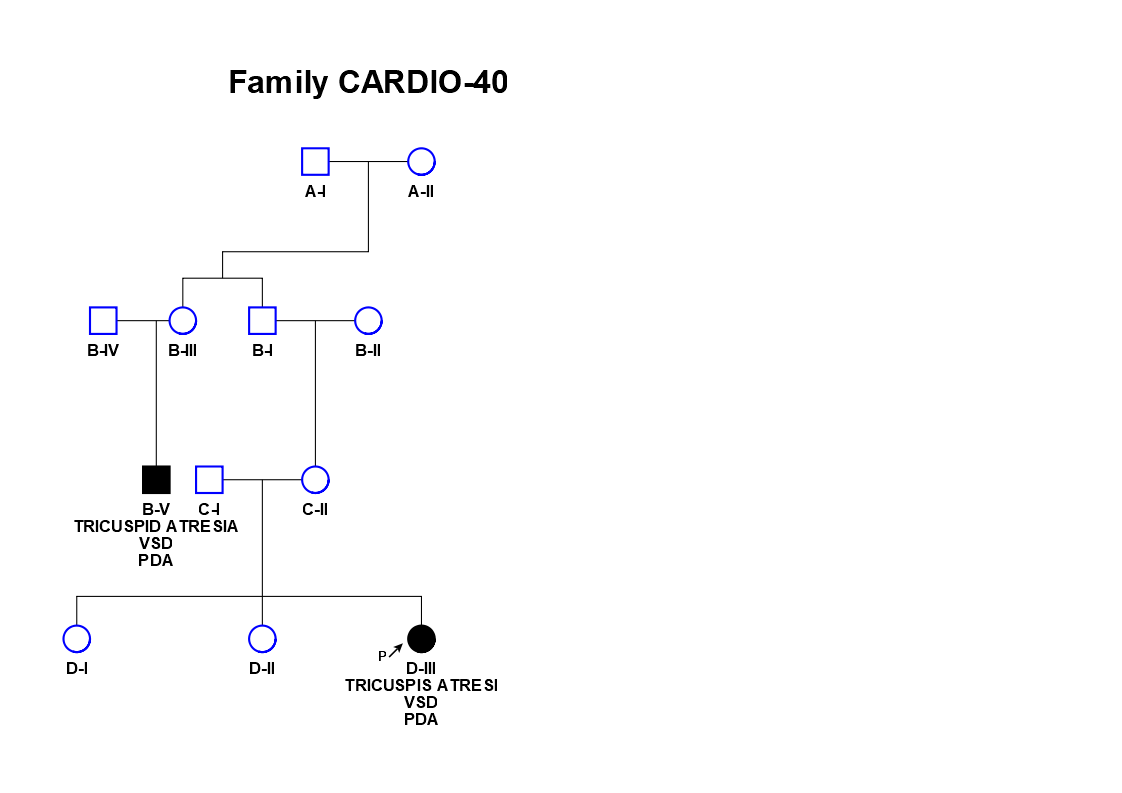

Supplement: Supplementary file 1 [file genes-13-01369-s001.zip › supplementary figures/pedigree/family-CARDIO-40.png]

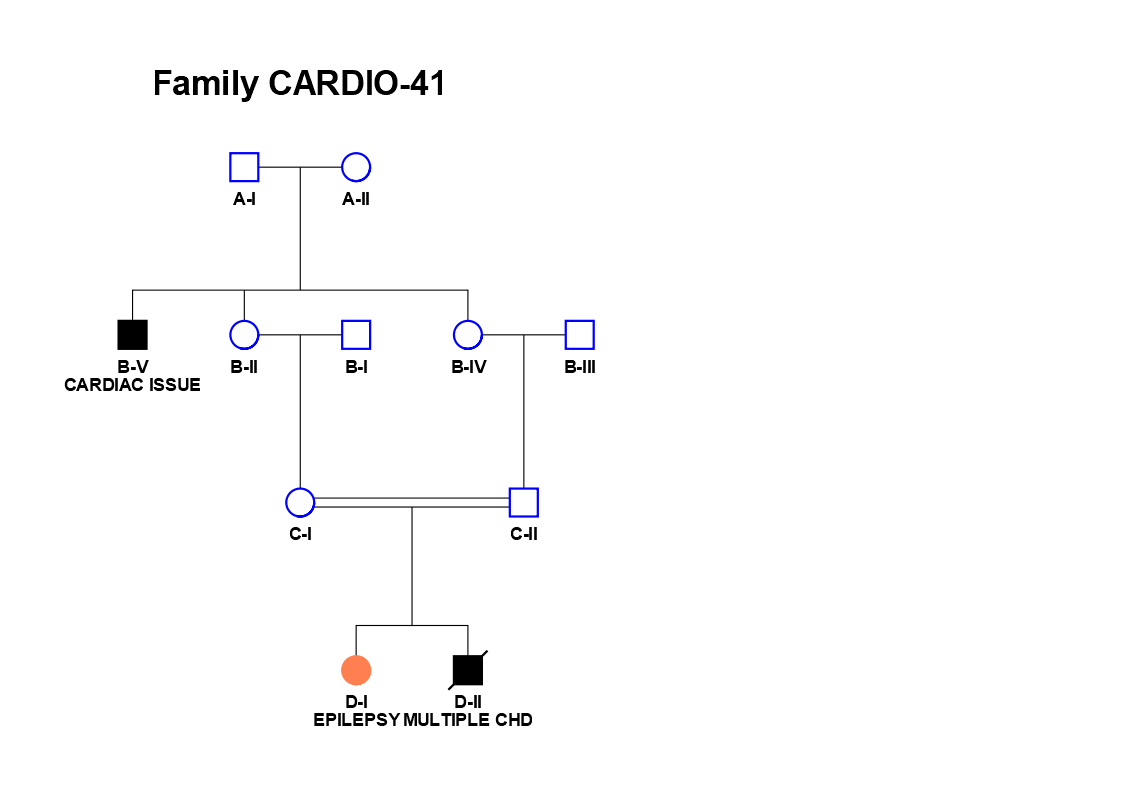

Supplement: Supplementary file 1 [file genes-13-01369-s001.zip › supplementary figures/pedigree/family-CARDIO-41.png]

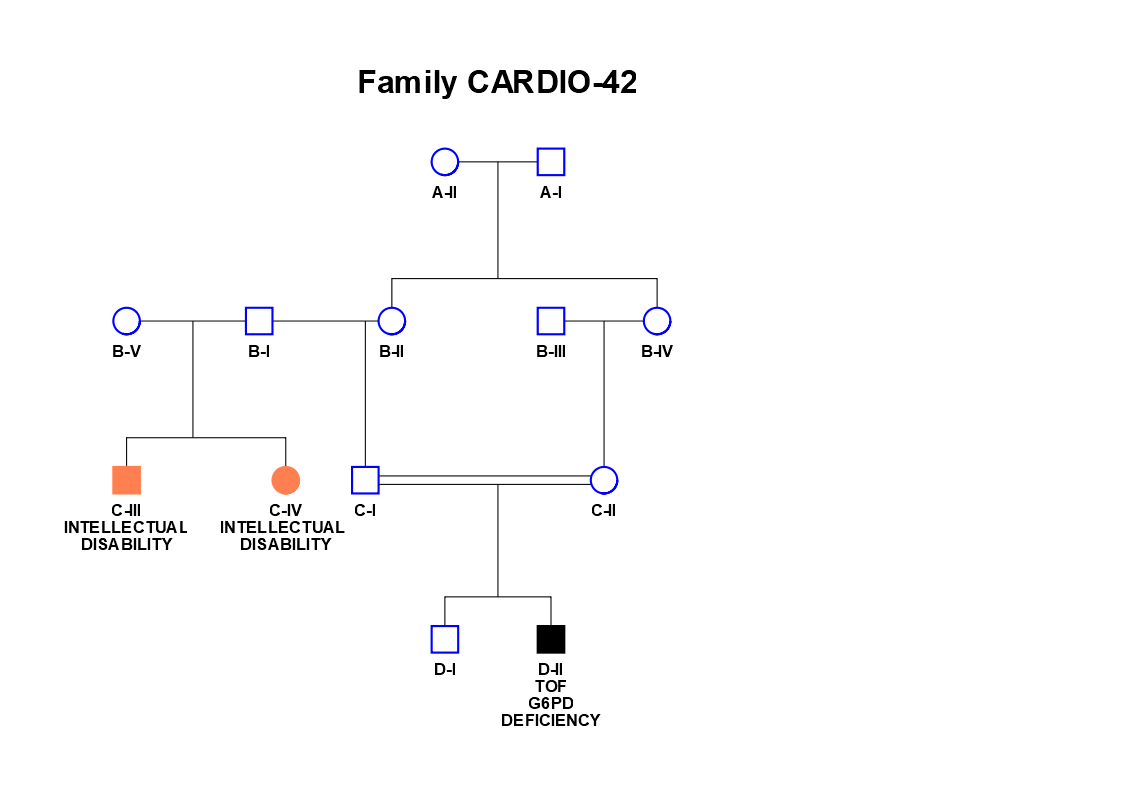

Supplement: Supplementary file 1 [file genes-13-01369-s001.zip › supplementary figures/pedigree/family-CARDIO-42.png]

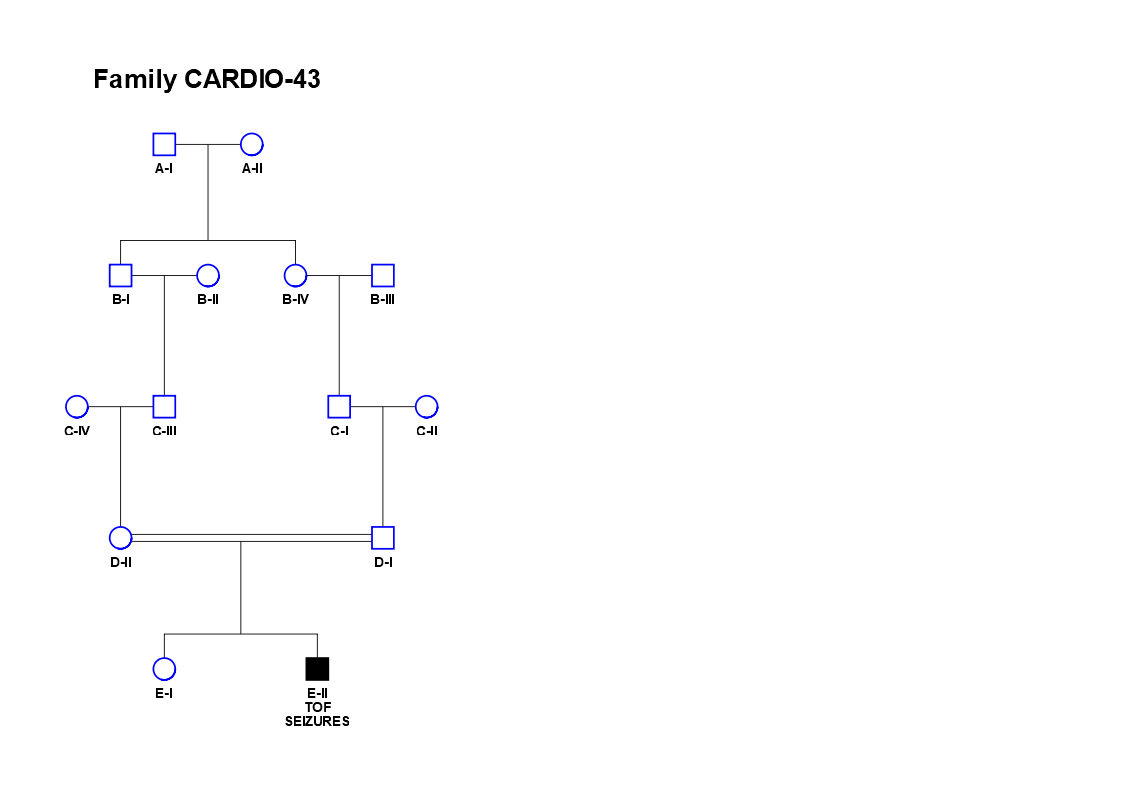

Supplement: Supplementary file 1 [file genes-13-01369-s001.zip › supplementary figures/pedigree/family-CARDIO-43.png]

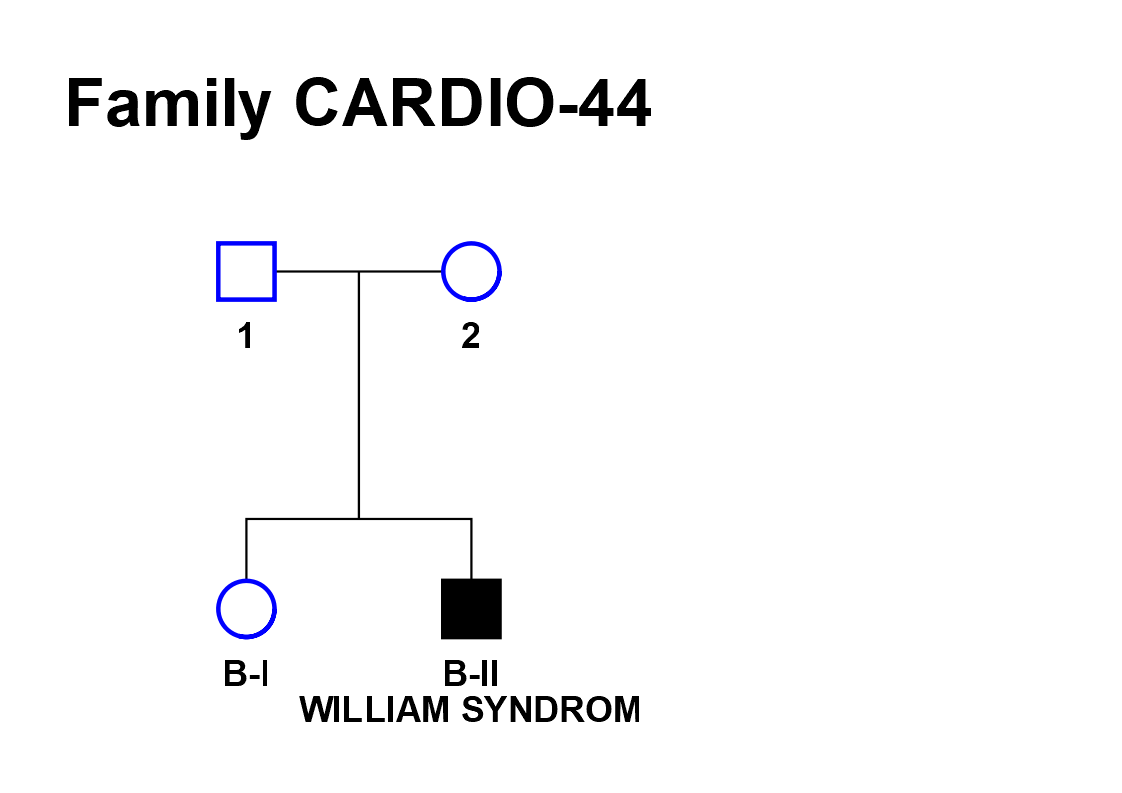

Supplement: Supplementary file 1 [file genes-13-01369-s001.zip › supplementary figures/pedigree/family-CARDIO-44.png]

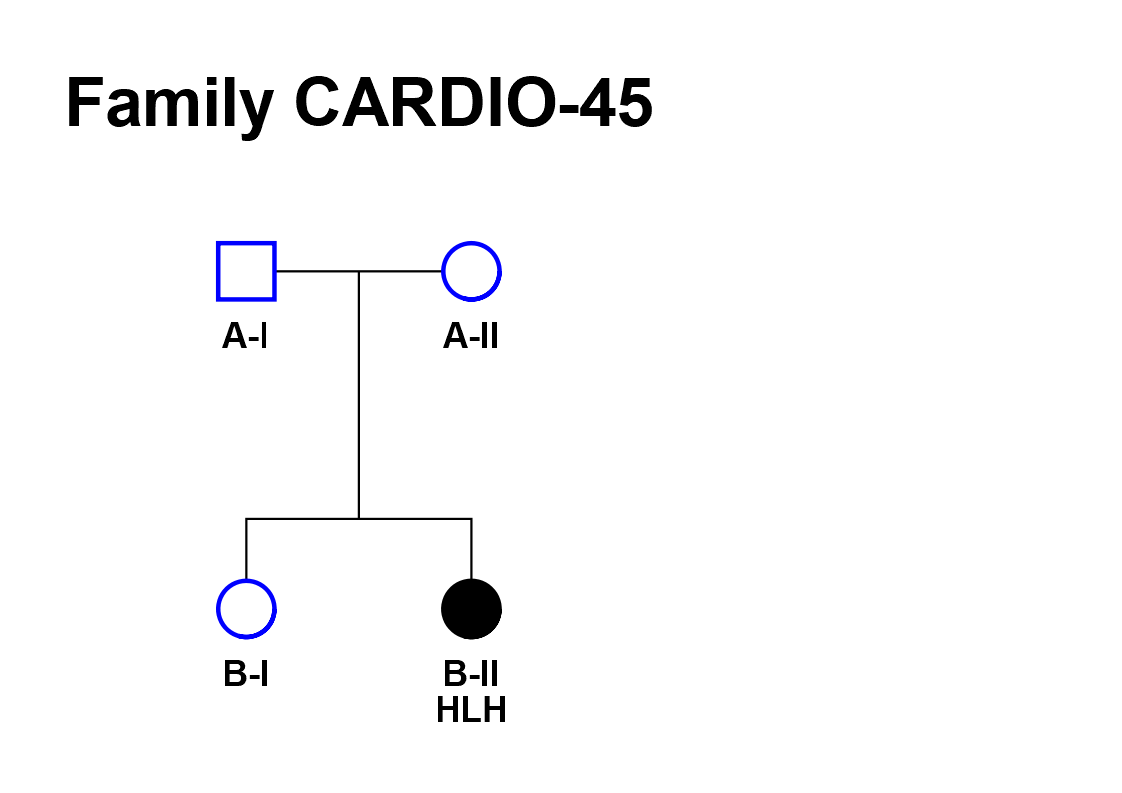

Supplement: Supplementary file 1 [file genes-13-01369-s001.zip › supplementary figures/pedigree/family-CARDIO-45.png]

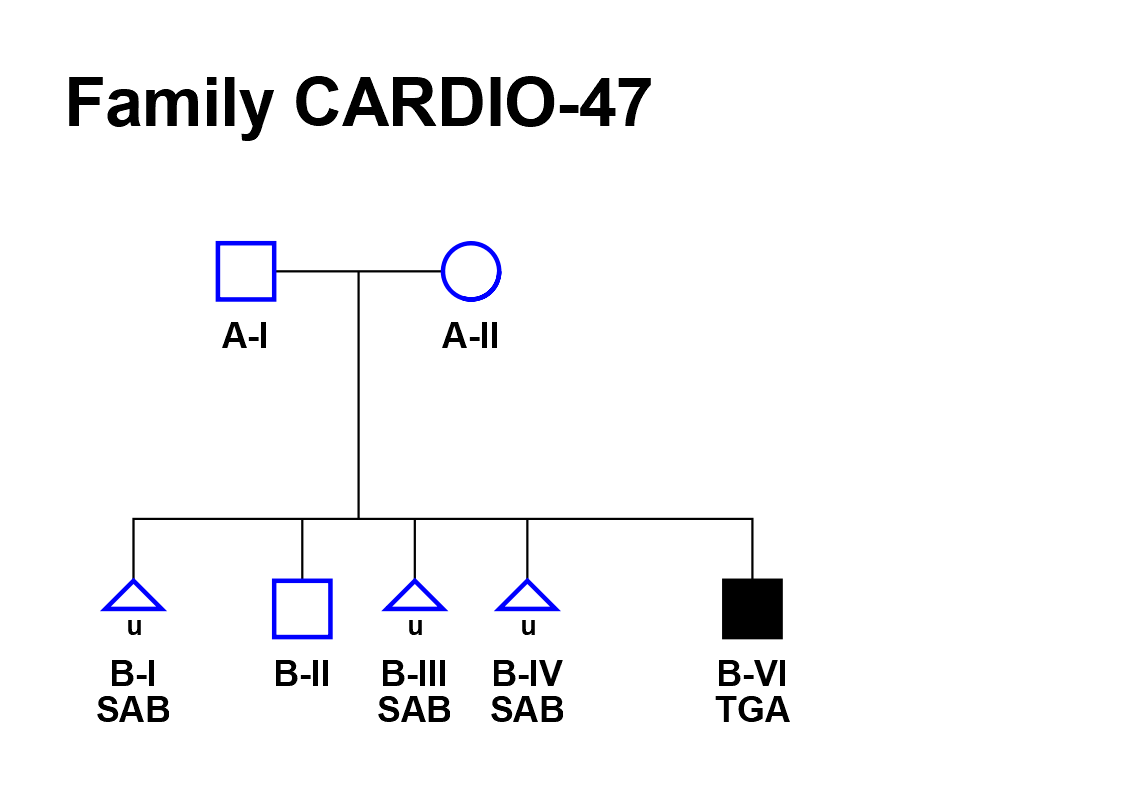

Supplement: Supplementary file 1 [file genes-13-01369-s001.zip › supplementary figures/pedigree/family-CARDIO-47.png]

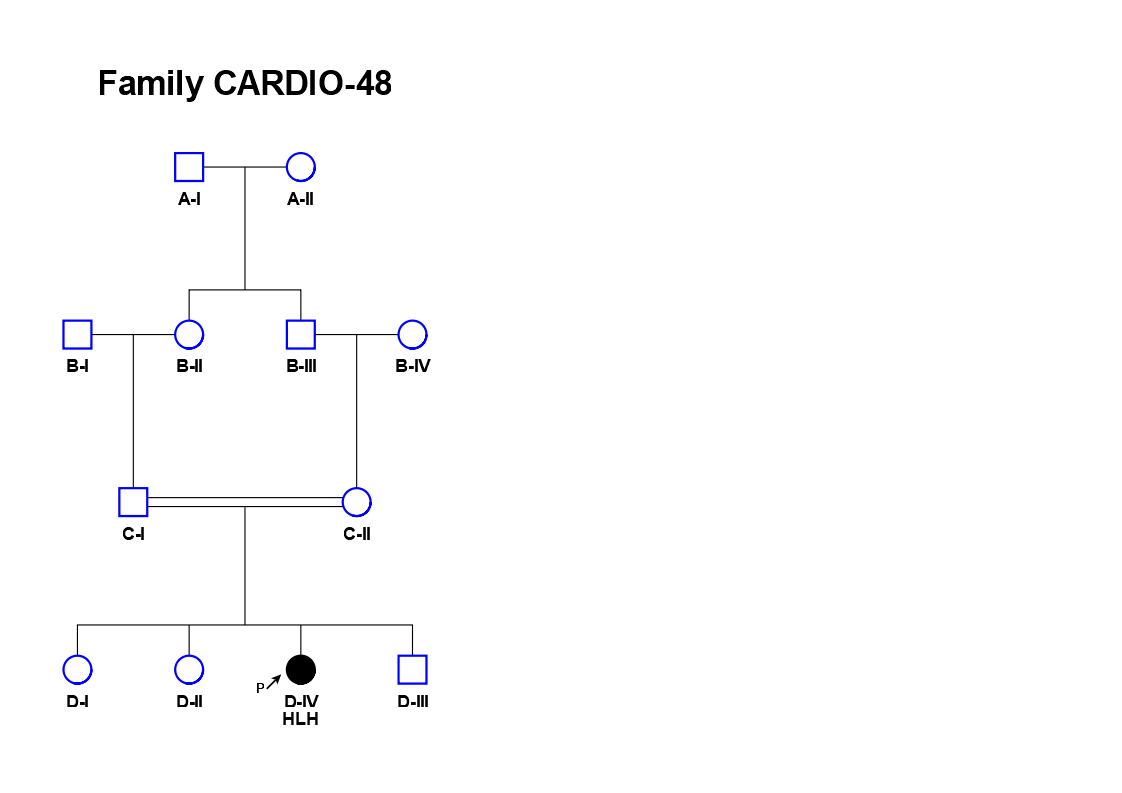

Supplement: Supplementary file 1 [file genes-13-01369-s001.zip › supplementary figures/pedigree/family-CARDIO-48.png]

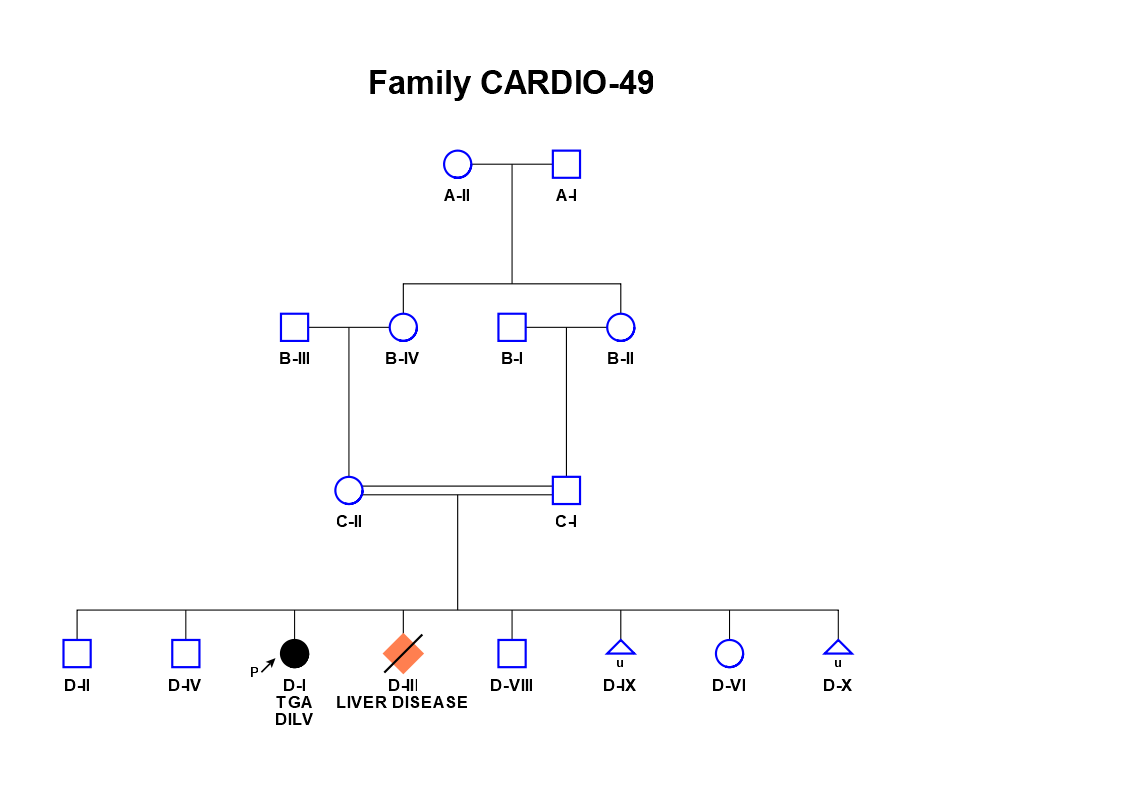

Supplement: Supplementary file 1 [file genes-13-01369-s001.zip › supplementary figures/pedigree/family-CARDIO-49.png]

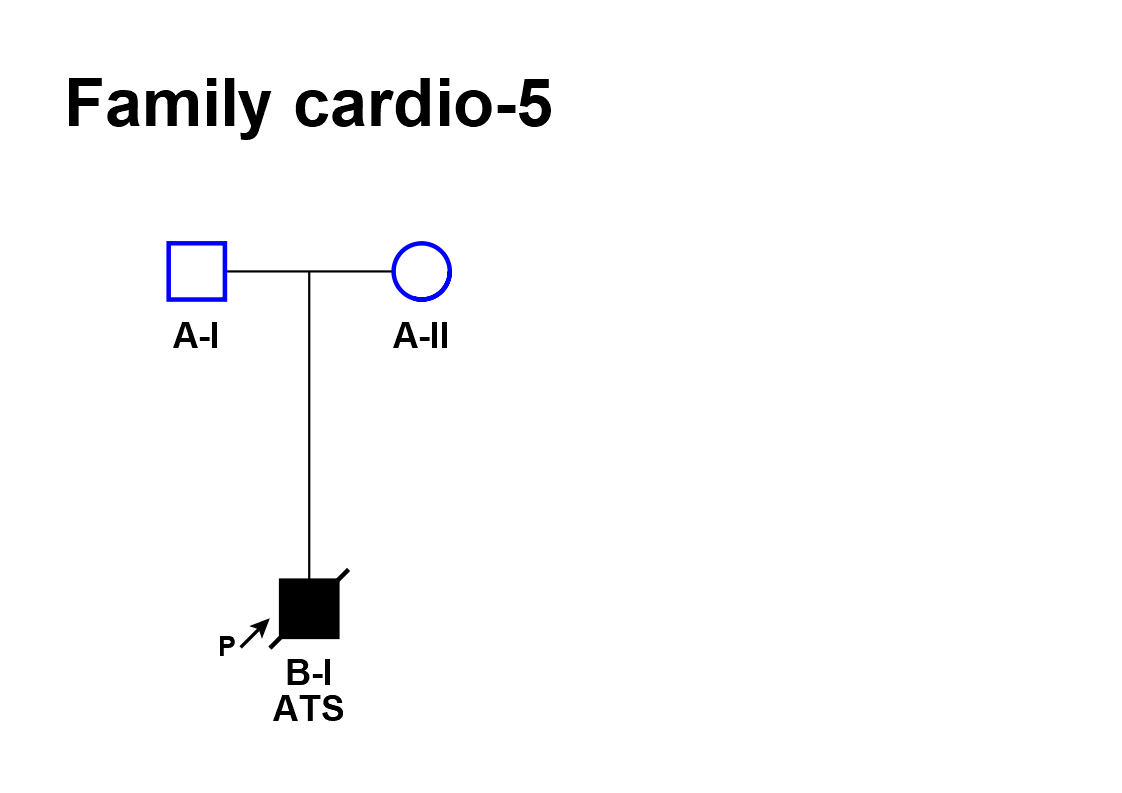

Supplement: Supplementary file 1 [file genes-13-01369-s001.zip › supplementary figures/pedigree/family-cardio-5.png]

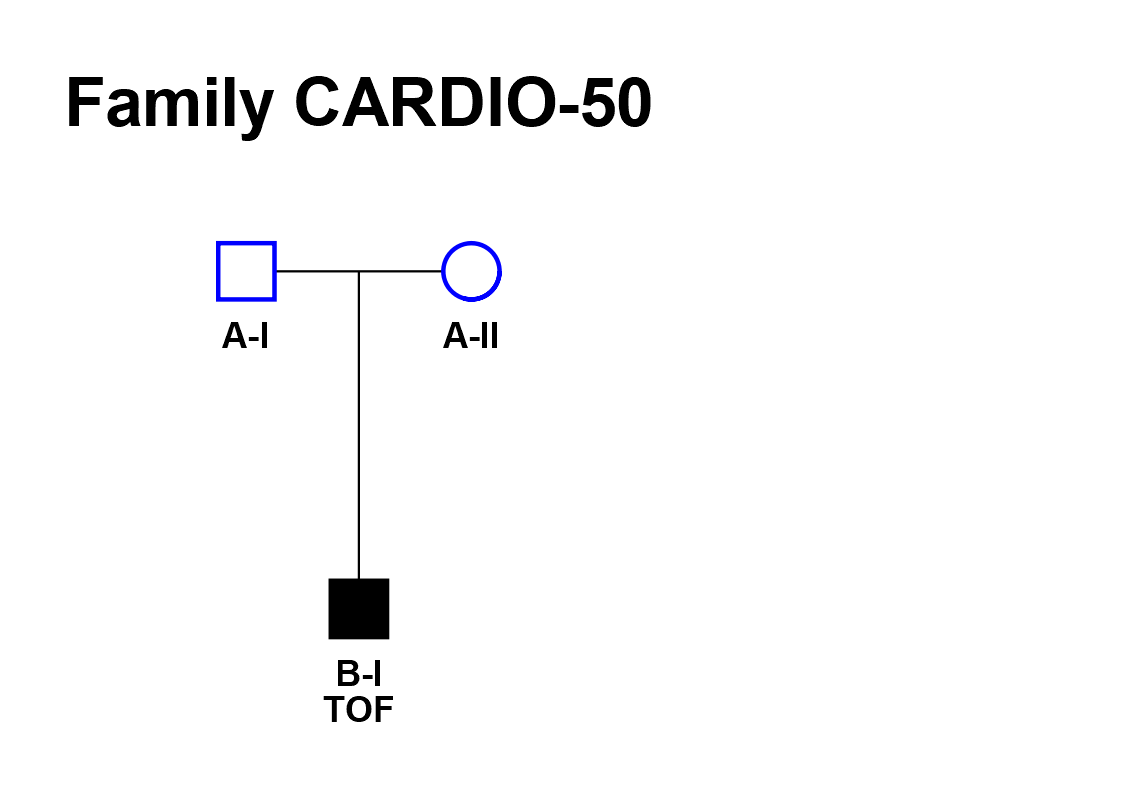

Supplement: Supplementary file 1 [file genes-13-01369-s001.zip › supplementary figures/pedigree/family-CARDIO-50.png]

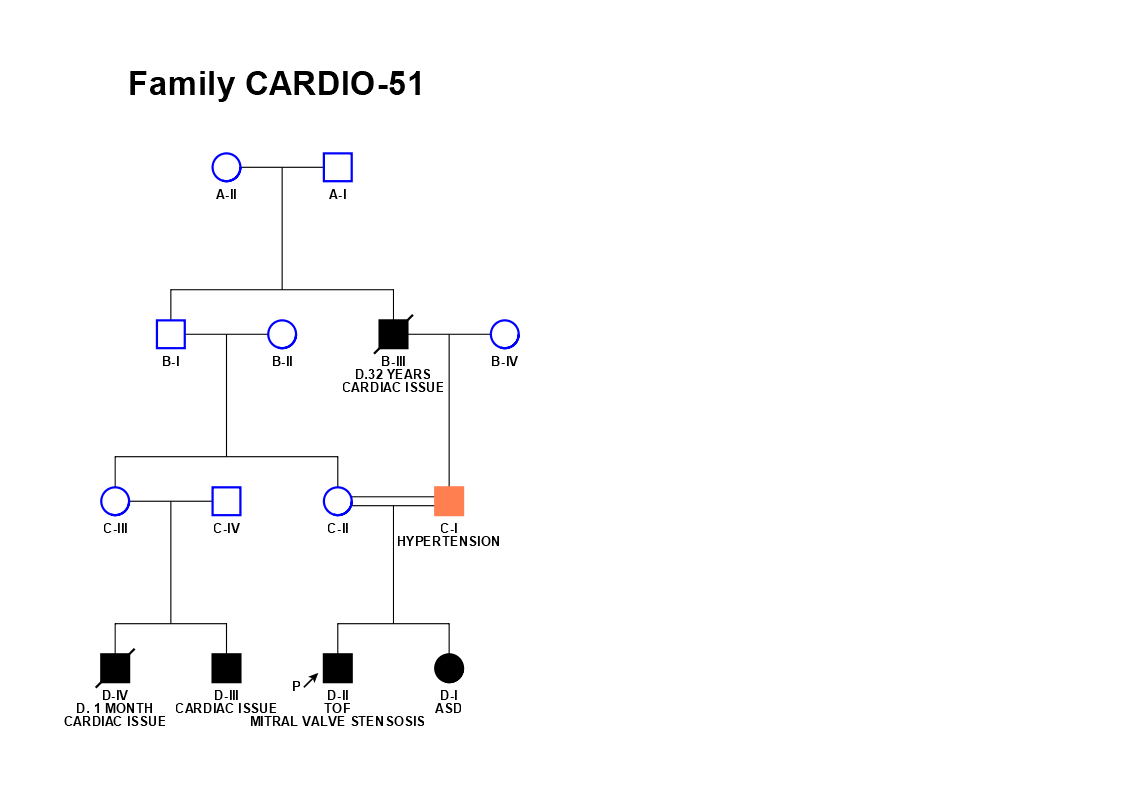

Supplement: Supplementary file 1 [file genes-13-01369-s001.zip › supplementary figures/pedigree/family-CARDIO-51 .png]

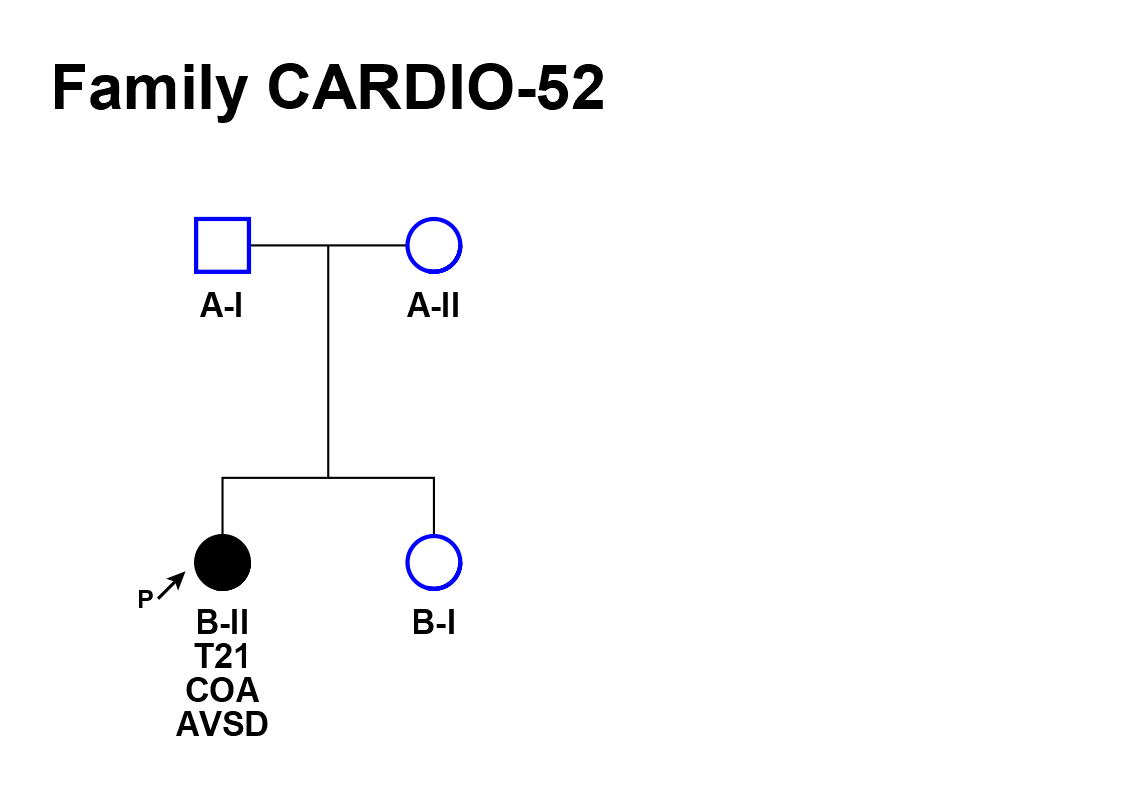

Supplement: Supplementary file 1 [file genes-13-01369-s001.zip › supplementary figures/pedigree/family-CARDIO-52.png]

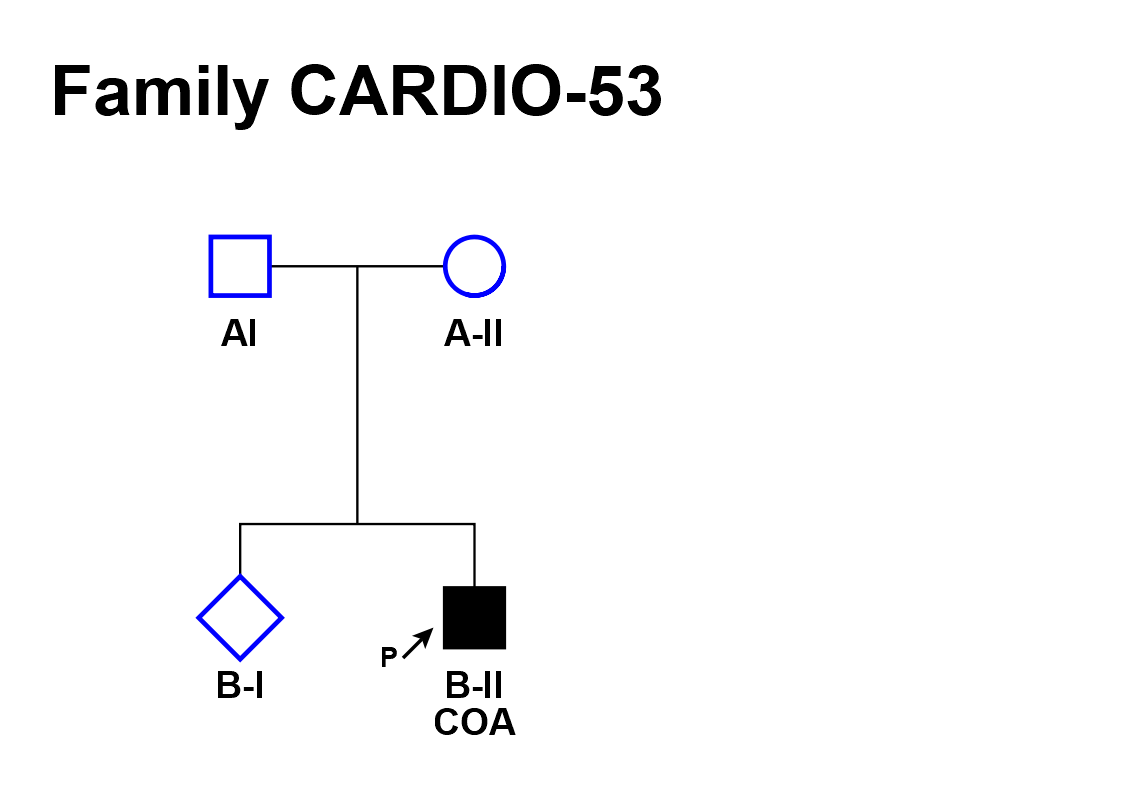

Supplement: Supplementary file 1 [file genes-13-01369-s001.zip › supplementary figures/pedigree/family-CARDIO-53.png]

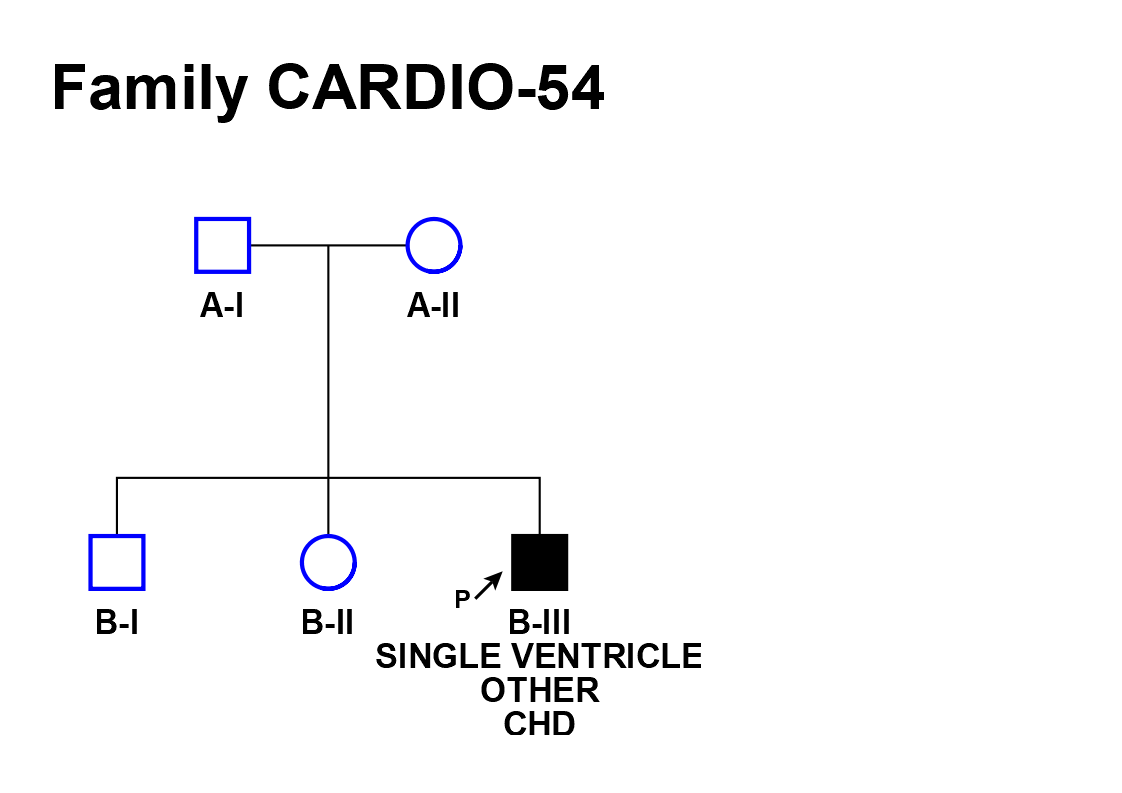

Supplement: Supplementary file 1 [file genes-13-01369-s001.zip › supplementary figures/pedigree/family-CARDIO-54.png]

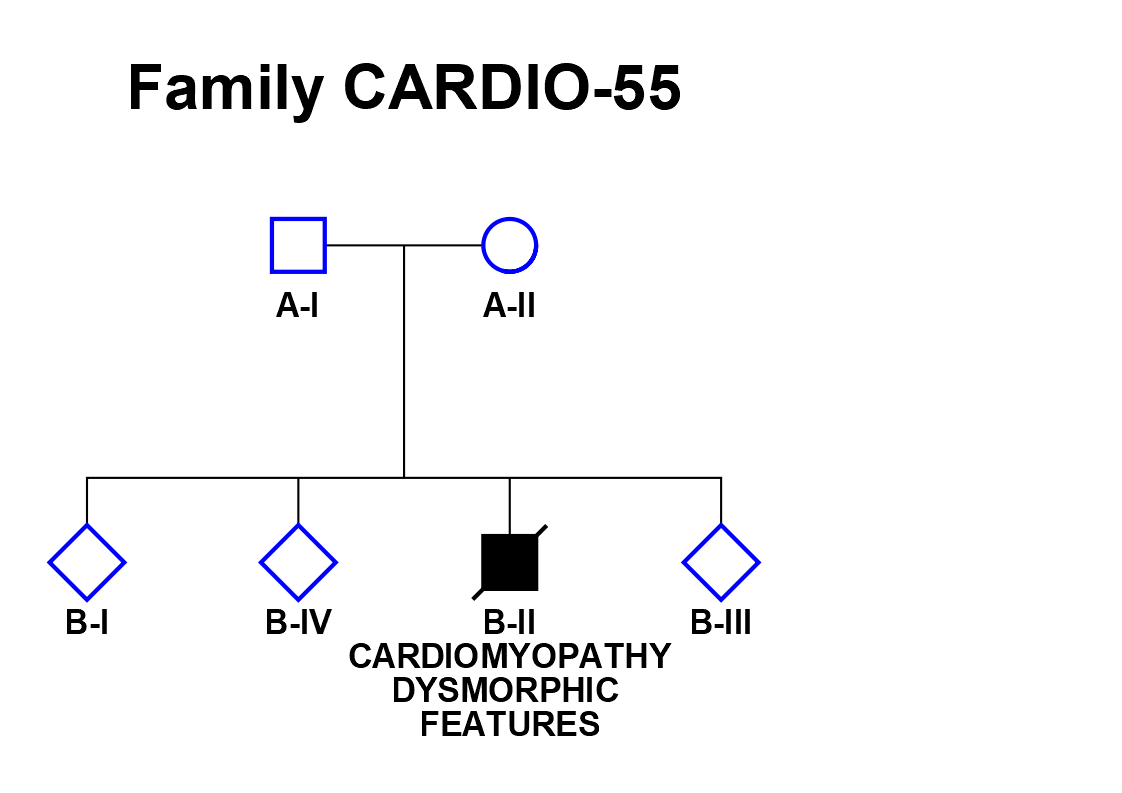

Supplement: Supplementary file 1 [file genes-13-01369-s001.zip › supplementary figures/pedigree/family-CARDIO-55.png]

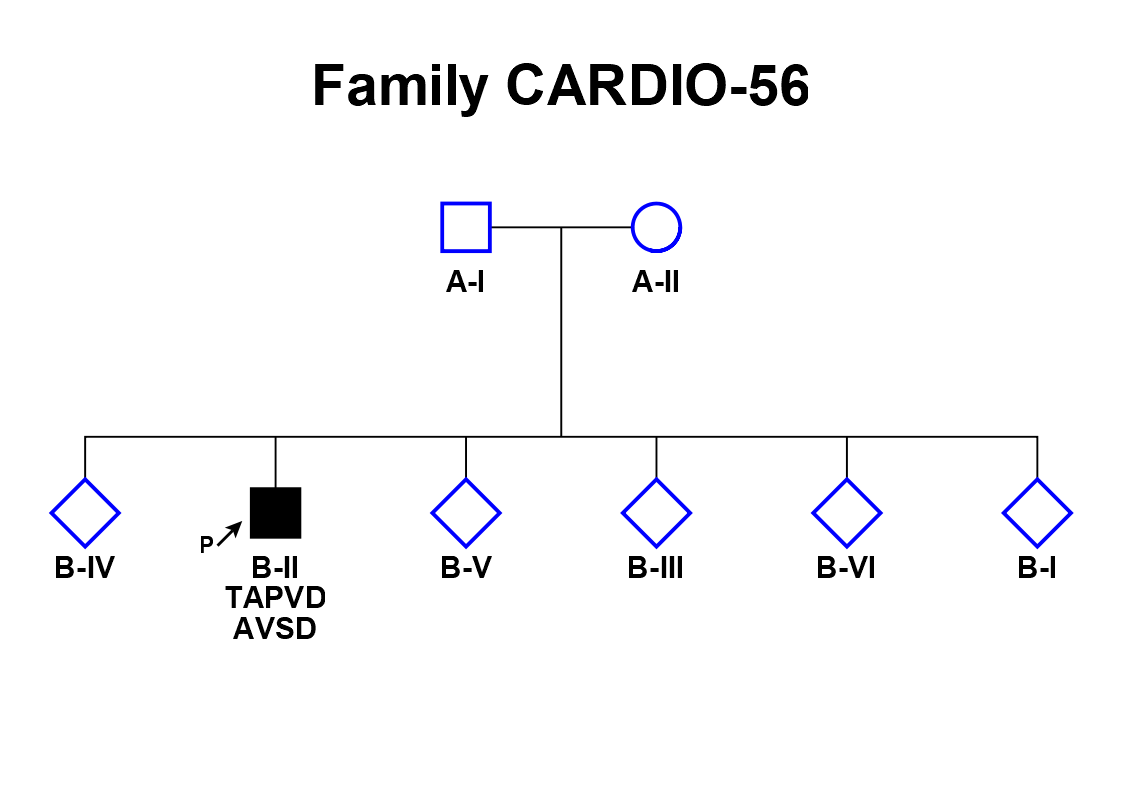

Supplement: Supplementary file 1 [file genes-13-01369-s001.zip › supplementary figures/pedigree/family-CARDIO-56.png]

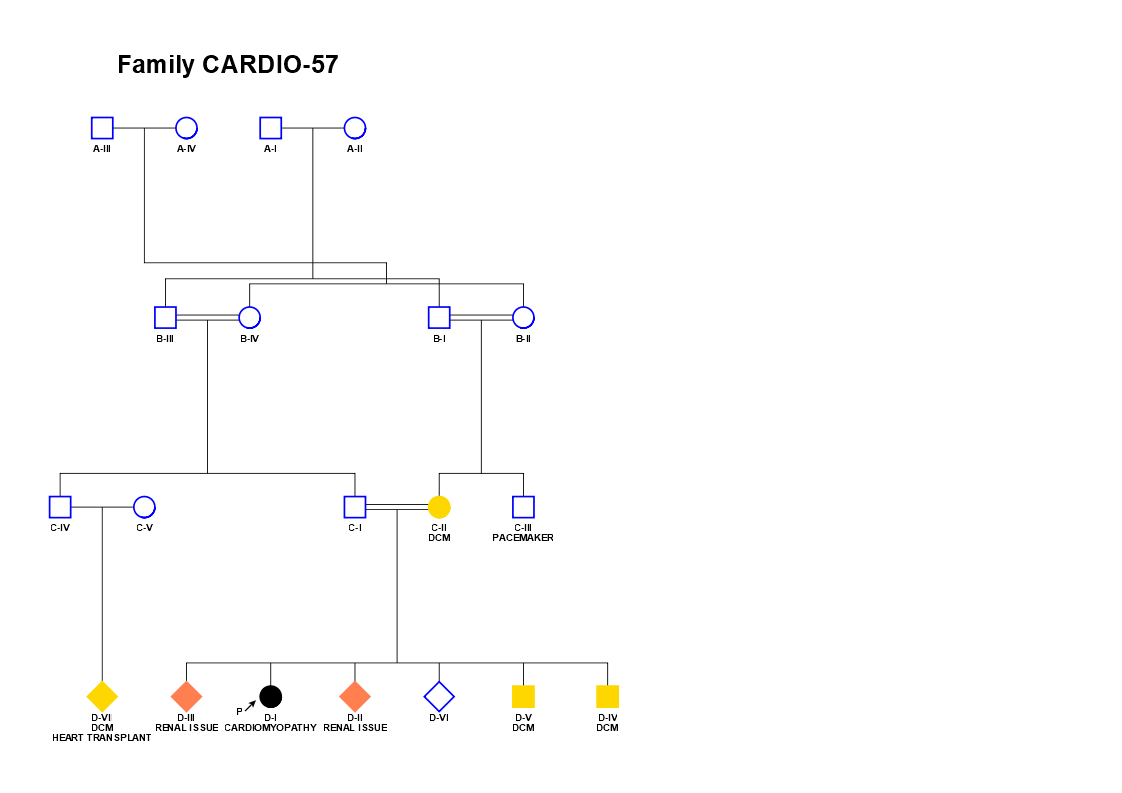

Supplement: Supplementary file 1 [file genes-13-01369-s001.zip › supplementary figures/pedigree/family-CARDIO-57.png]

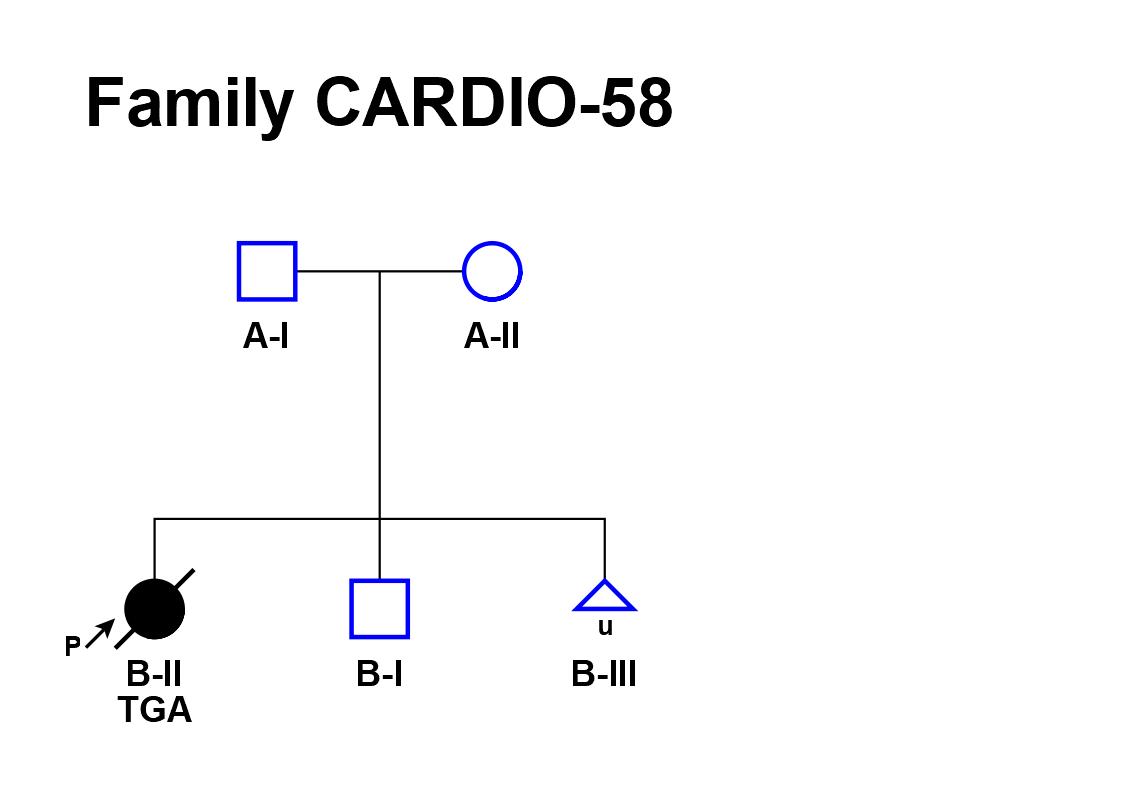

Supplement: Supplementary file 1 [file genes-13-01369-s001.zip › supplementary figures/pedigree/family-CARDIO-58.png]

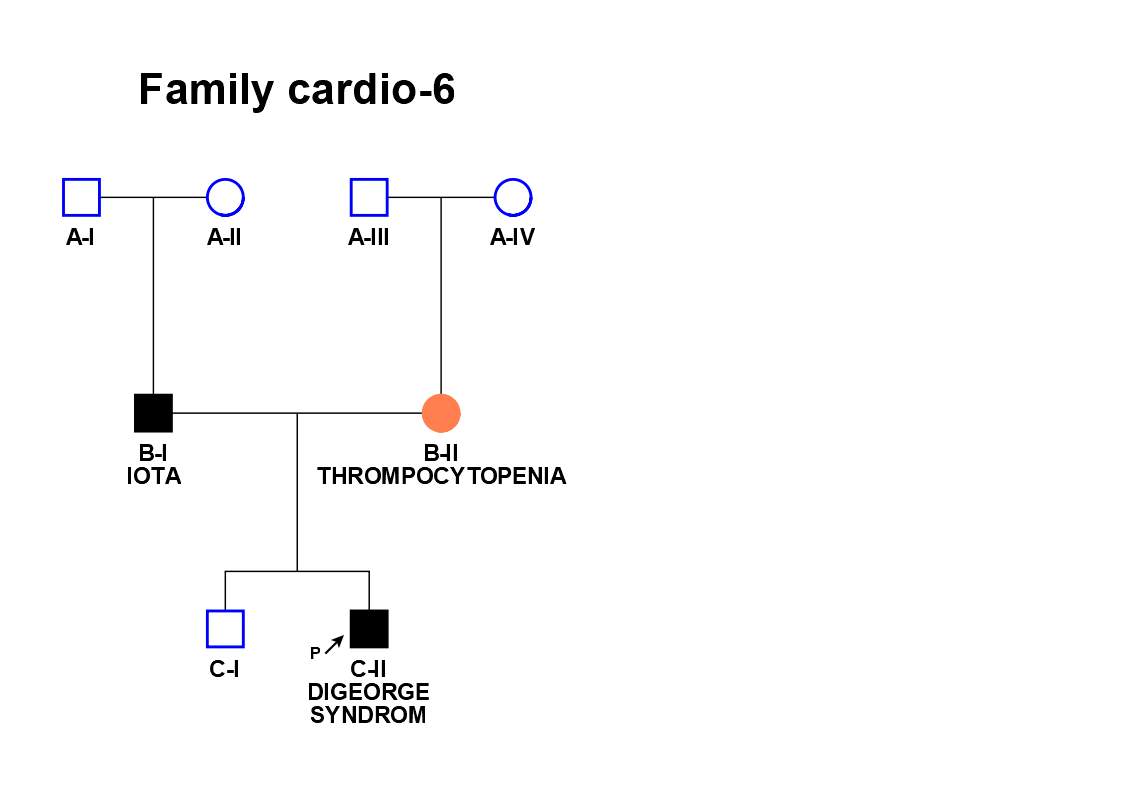

Supplement: Supplementary file 1 [file genes-13-01369-s001.zip › supplementary figures/pedigree/family-cardio-6.png]

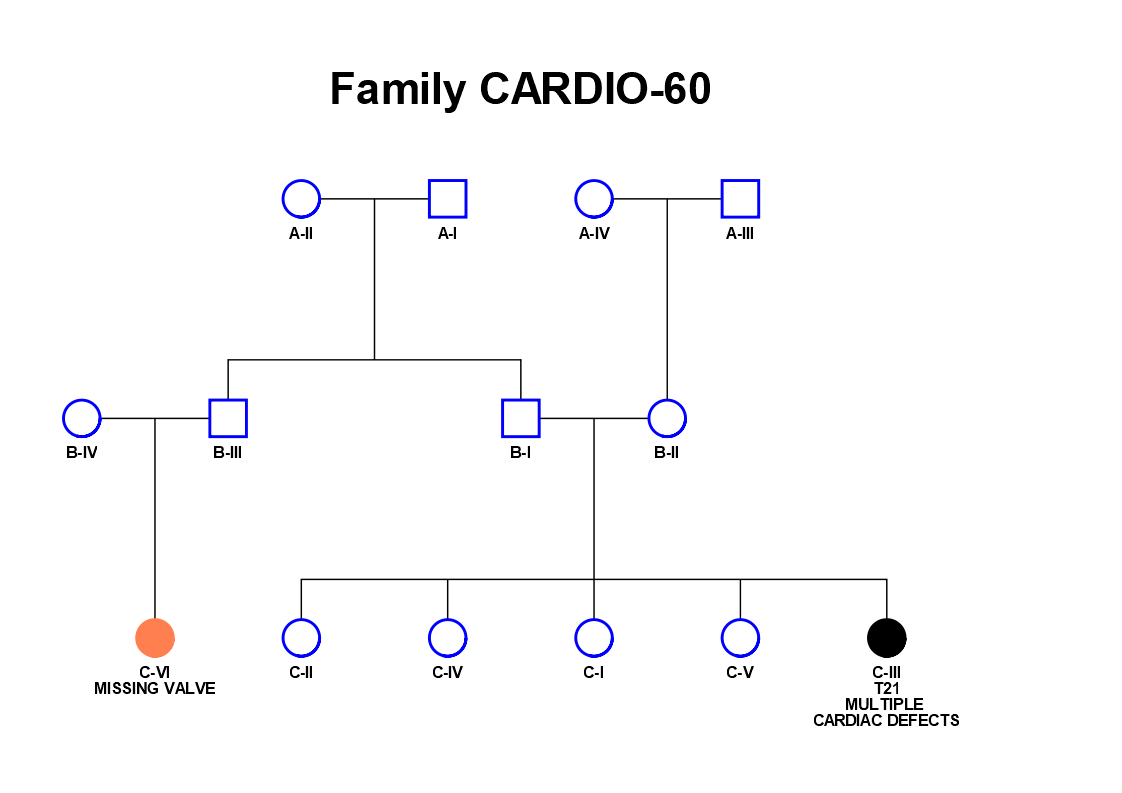

Supplement: Supplementary file 1 [file genes-13-01369-s001.zip › supplementary figures/pedigree/family-CARDIO-60.png]

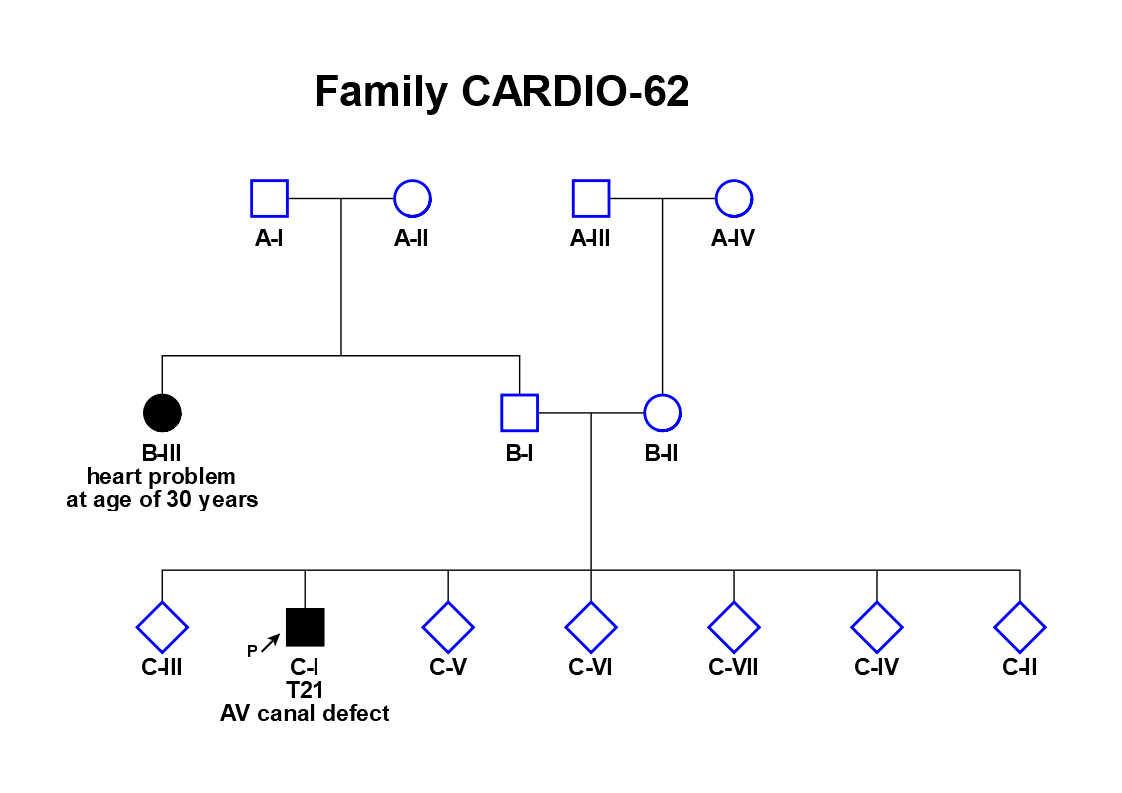

Supplement: Supplementary file 1 [file genes-13-01369-s001.zip › supplementary figures/pedigree/family-CARDIO-62.png]

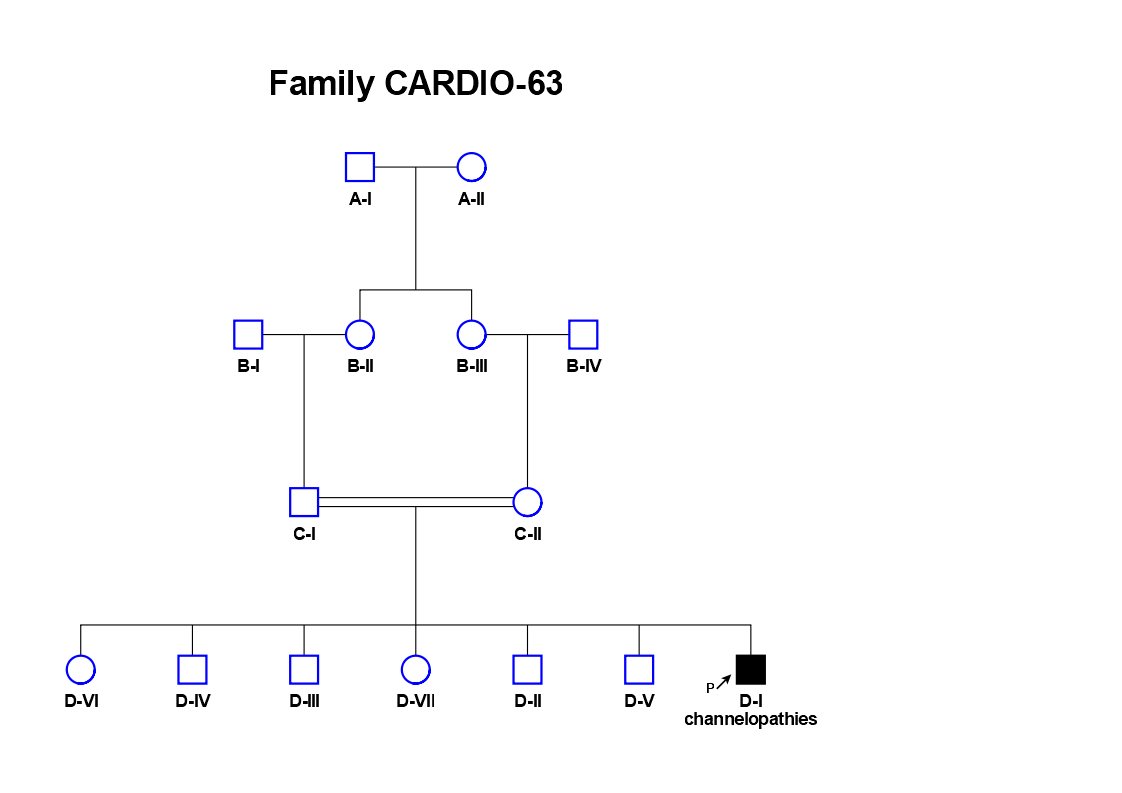

Supplement: Supplementary file 1 [file genes-13-01369-s001.zip › supplementary figures/pedigree/family-CARDIO-63.png]

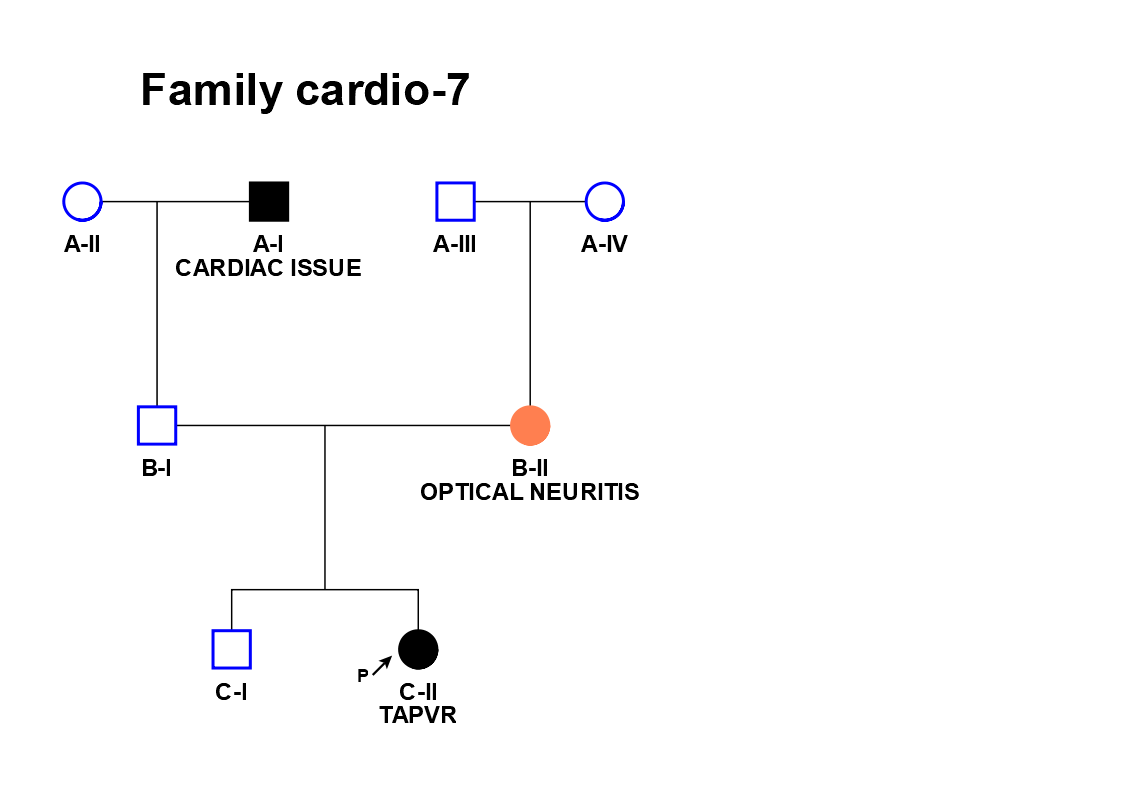

Supplement: Supplementary file 1 [file genes-13-01369-s001.zip › supplementary figures/pedigree/family-cardio-7.png]

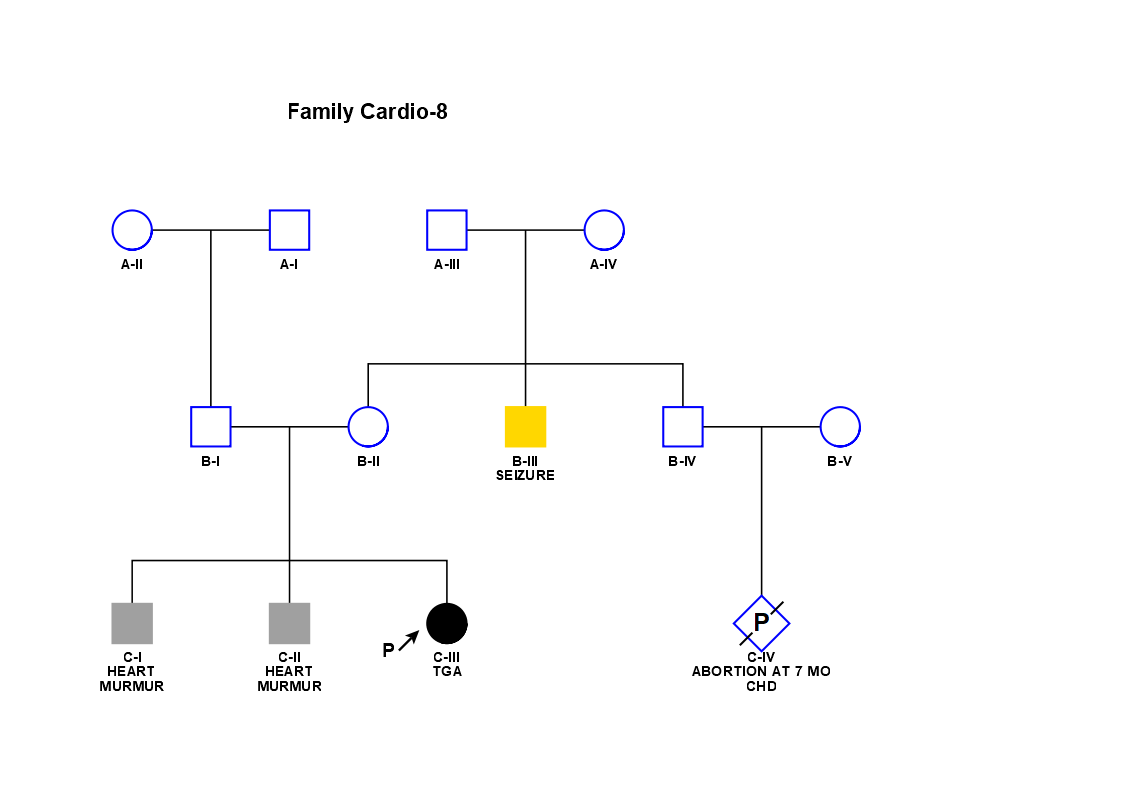

Supplement: Supplementary file 1 [file genes-13-01369-s001.zip › supplementary figures/pedigree/family-Cardio-8.png]

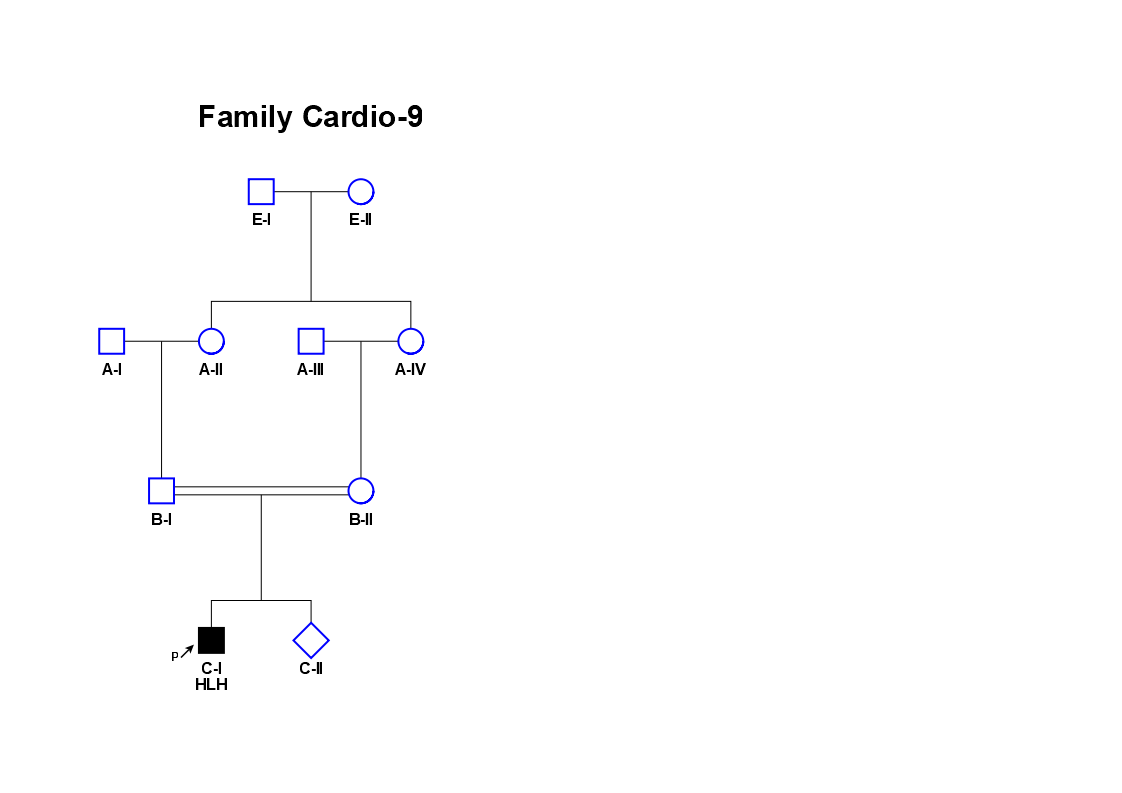

Supplement: Supplementary file 1 [file genes-13-01369-s001.zip › supplementary figures/pedigree/family-Cardio-9.png]
