# Supplementary material for: Investigation of Genetic Causes in Patients with Congenital Heart Disease in Qatar: Findings from the Sidra Cardiac Registry
Source: Genes (Basel). 2022 Jul 30;13(8):1369. doi: 10.3390/genes13081369 (PMC9407366; doi:10.3390/genes13081369)
Supplement: Supplementary file 1 [file genes-13-01369-s001.zip › supplementary tables/Suplemntary Tables compressed.zip!/Suplemntary Tables compressed/Table S3.docx]

| Table S3 :Summary of the shared variants identified in 24 CHD patients through whole exome sequencing (WES) analysis | | | | | | | | | | | | | | | | | |
| --- | --- | --- | --- | --- | --- | --- | --- | --- | --- | --- | --- | --- | --- | --- | --- | --- | --- |
| Patient ID | Cardiac Phenotype | Extra Cardiac phenotype | Age | Gender | Country of origin | Gene | Protein change | Nucleotide change | Gemini  segregation | Zygosity | Inheritance | ClinVar | ACMG/AMP classification | ACMG/AMP Sub classification | Reported in other studies | Reported cases country of origin | *References* |
| Cardio-2.A | TOF | None | 6 months | F | Qatar | *TTN* | p.Asn32797Ser | c.98390A>G | CH | Heterozygous | Paternal | Conflict interpretation | Likely benign | BP1, BP6, PP3 | Late onset progressive distal myopathy | European | [1] |
| Cardio-5.A | ATS | None | 2 months | M | Yemen | *SLC2A10* | p.Ser81Arg | c.243C>G | AR | Homozygous | Both parents | Pathogenic | Likely pathogenic | PM1,PM2,PP3,PP5 | ATS | Qatari, KSA | [2, 3] |
| Cardio-11.A | ASD | Squint, polydactyly | 3 months | F | India | *PRR12* | p.Gly1813Arg | c.5437G>A | AR | Homozygous | Both parents | None | US | PM1,BP1 | none | - | - |
| Cardio-12.A | multiple CHD | Failure to thrive | 20 days | F | Sri Lanka | *KMT2D* | p.Pro3665Ala | c.10993C>G | CH | Heterozygous | Maternal | Conflicting interpretation | US | BP1 | Kabuki syndrome | Unknown | [4] |
| Cardio-15.A | Shone's complex (hypoplastic transverse arch, VSD, PDA) | None |  | F | Pakistan | *FLT1* | - | c.1437-6delT | *de novo* | Heterozygous | None | None | none | - | none | - | - |
|  |  |  | 7 days |  |  | *CCDC141* | p.Val1457Ile | - | AR | Homozygous | Both parents | Likely Benign | Likely benign | BS1,BP6 | Reported  (unknown condition) | - | [4] |
| Cardio-16.A | TOF | None |  | M | Sudan | *SORBS2* | p.Phe1188Leu | c.3564C>A | Could not be identified | Heterozygous | Not reported * | None | US | PM1 | none | - | - |
|  |  |  | 9 months |  |  | *DYNC2H1* | p.Val1899Ile | c.5695G>A | CH | Heterozygous | Maternal | Conflicting interpretation | US | PM1,PM2 | Short rib Thoracic dysplasia | Multiple submitters(unknown ethnicities) | [5] |
|  |  |  |  |  |  | *BMP10* | p.Thr200Ser | c.599C>G | *de novo* | Heterozygous | None | None | US | PM1,BS1 | none | - | - |
|  |  |  |  |  |  | *KYNU* | p.Thr25Met | c.74C>T | *de novo* | Heterozygous | None | Benign | US | PM1,PM2,BP6 | none | - | - |
|  |  |  |  |  |  | *PLCD4* | p.Val364Met | c.1090G>A | *de novo* | Heterozygous | None | None | US | PM1,BP4 | none | - | - |
|  |  |  |  |  |  | *MLIP* | p.Pro459Arg | c.1376C>G | *de novo* | Heterozygous | None | None | US | PM1 | none | - | - |
|  |  |  |  |  |  | *PBX3* | p.His401Gln | c.1203T>A | *de novo* | Heterozygous | None | None | US | PM1,PM2 | none | - | - |
|  |  |  |  |  |  | *OR51E1* | p.Ala156Thr | c.466G>A | *de novo* | Heterozygous | None | None | US | PM1 | none | - | - |
| Cardio-18.A | Aortic stenosis & insufficiency, mild LVH, frequent PACs | none |  | M | Qatari | *CMYA5* | p.Ser2012Phe | c.6035C>T | AR | Homozygous | One copy was maternally inherited( all siblings are carrriers)** | Benign | likely benign | BS1,BP6 | Reported (unknown condition) | Unknown | [6] |
|  |  |  | 18 years |  |  | *CMYA5* | p.Glu584Ala | c.1751A>C | AR | Homozygous | One copy was maternally inherited( all siblings are carriers)** | Benign | likely benign | PM1,PM2 | Reported (unknown comnditon) | Unknown | [7] |
| Cardio-27.A | TOF | Prolonged QT interval, hearing loss |  | F | Yemen | *CHD7* | p.Arg2098* | c.6292C>T | *de novo* | Heterozygous | none | Pathogenic | Pathogenic | PVS1,PM2,PP3,PP5 | Immune deficiency, CHARGE syndrome | China | [8, 9] |
| Cardio-31.A | HLH | None |  | F | Sudan | *DNAH11* | c.9946-7T>C | - | AR | Homozygous | One copy paternally inherited *** | None | None | PM2 | none | - | - |
|  |  |  |  |  |  | *DNAAF3* | p.Val536Met | c.1606G>A | AR | Homozygous | One copy paternally inherited *** | Benign/likely benign | Likely Bening | PM1,BS1,BP1,BP4,BP6 | Hypertrophic cardiomyopathy, Dilated Cardiomyopathy, Recessive, Familial restrictive cardiomyopathy, Primary ciliary dyskinesia | - | - |
|  |  |  | 6 years |  |  | *DNAAF3* | p.Trp44Leu | c.131G>T | AR | Homozygous | One copy paternally inherited *** | Benign | likely benign | PM1,BS1,BP1,BP6 | Primary ciliary dyskinesia | - | - |
|  |  |  |  |  |  | *COL5A2* | p.Arg956Pro | c.2867G>C | CH | Heterozygous | Paternal | Benign | Likely benign | PM1,BS1,BP6 | Ehler Danlos Syndrome, cardiovascular phenotype | Unknown | [10] |
|  |  |  |  |  |  | *SMYD4* | p.Tyr295Cys | c.884A>G | AR | Homozygous | One copy paternally inherited *** | None | US | PM1 | none | - | - |
| Cardio-36.A | TOF | None |  | F | Sudan | *IFT172* | p.Val1204Leu | c.3610G>C | CH | heterozygous | Maternal | Benign /likely benign | US | BP6 | Retinitis pigmentosa 71,Short-rib thoracic dysplasia 10 with or without polydactyly | Unknown | [11] |
|  |  |  | 5 months |  |  | *IFT172* | p.Arg1134Leu | c.3401G>T | CH | Heterozygous | Paternal | Benign/likely benign | Likely benign | BS1,BP6 | Retinitis pigmentosa 71,Short-rib thoracic dysplasia 10 with or without polydactyly | Unknown | [11] |
|  |  |  |  |  |  | *SLC24A4* | p.Gln22His | c.66A>T | CH | Heterozygous | Paternal | None | US | PM1,PM2 | none | - | - |
|  |  |  |  |  |  | *ATP10D* | p.Arg266His | c.797G>A | AD | Heterozygous | Maternal(brother is also carrier) | None | US | PM1,PM3 | none | - | - |
|  |  |  |  |  |  | *CDH20* | p.Gln371Arg | c.1112A>G | AR | homozygous | Both parents | None | US | PM1,BS1 | none | - | - |
|  |  |  |  |  |  | *PLEC* | p.Ala1545Val | c.4634C>T | CH | Heterozygous | Maternal | Benign | US | PM1,PM2 | Limb-girdle muscular dystrophy, type 2Q.Epidermolysis bullosa simplex with muscular dystrophy | Unknown | [12] |
| Cardio-38.A | Poly Valvular disease, Mitral valve prolapse | None | 11 years | M | Qatar | *LRRC56* | p.Pro305His | c.914C>A | Could not be identified | Heterozygous | Not reported * | None | US | - | none | - | - |
| cardio-40.A | Tricuspid Atresia, restrictive VSD,Large PDA | None | 5 months | F | India | *DNAH9* | p.Arg1517Gln | c.4550G>A | CH | Heterozygous | Maternal | None | US | PM1 | Crisponi/CISS1-like and Bohring-Opitz like syndrome | Turkish | [13] |
|  |  |  |  |  |  | *SAAL1* | p.Pro95Leu | c.284C>T | CH | Heterozygous | Paternal | None | US | PM1 | none | - | - |
| Cardio-41.A | Fontan,Right isomerism,Interrupted IVC, mitral atresis, DORV,Large VSD, severe PS | None | 16 years | F | Yemen | *DYNC2H1* | p.Leu592Phe | c.1774C>T | AD | Heterozygous | Maternal | Uncertain significant | US | PM2,PP3 | Intellectual disability, Jeune thoracic dystrophy, Short-rib thoracic dysplasia 3 with or without polydactyly | Unknown | [14] |
| Cardio-42.A | TOF | None | 6 months | M | Kuwait | *DNAH5* | p.Asn1420Asp | c.4258A>G | CH | Heterozygous | Paternal | VUS | US | PM1,PM2 | Primary ciliary dyskinesia | Unknown | [15] |
| Cardio-47.A | TGA | None | 15 days | M | Egypt | *INPP5F* | p.Leu131Met | c.391C>A | CH | Heterozygous | Paternal | None | US | PM1,PM2 | None | - | - |
| Cardio-48.A | HLH | None |  | F | Pakistan | *SHROOM3* | p.Gln1623Lys | c.4867C>A | AR | Homozygous | Both parents | None | US | PM1 | None | - | - |
|  |  |  |  |  |  | *SCN10A* | p.Ser470Tyr | c.1409C>A | CH | Heterozygous | Maternal | None | US | BP4, BP1, BS1, PP3 | None | - | - |
|  |  |  | 20 days |  |  | *PDE4DIP* | p.Gly1542Arg | c.4624G>A | CH | Heterozygous | Paternal | Likely benign | US | PM2 | None | - | - |
| Cardio-49.A | DILV, TGA | None |  | F | Pakistan | *NUP210* | p.Pro1742Arg | c.5225C>G | CH | Heterozygous | Maternal | None | US | PM2 | None | - | - |
|  |  |  | 22 days |  |  | *COL6A2* | p.Arg784His | c.2351G>A | CH | Heterozygous | Maternal | Benign /likely benign | US | PM1,PM2,BP6 | None | - | - |
| Cardio-50.A | TOF | None | 4 months | M | India | *GLA* | p.Asp313Tyr | c.937G>T | UPD | Homozygous | Maternal | Conflicting interpretations of pathogenicity | US | BP6, PP3 | Hypertrophic cardiomyopathy, Fabry disease, Angiokeratoma corporis diffusum,sudden unexplained death, cardiac variant | Unknown | [16] |
| Cardio-53.A | COA | None | 3 months | M | Morocco | *KARS* | p.Glu120Gln | c.358G>C | CH | Heterozygous | Maternal | None | US | PM1,PM2 | None | - | - |
|  |  |  |  |  |  | *PLEC* | p.Gln2111His | c.6333G>C | CH | Heterozygous | Maternal | None | US | PM1,PM2,PP3 | none | - | - |
|  |  |  |  |  |  | *ANKS6* | p.Pro736Ala | c.2206C>G | CH | Heterozygous | Maternal | None | US | PM1,PM2,PP3,BP6 | None | - | - |

-Abbreviations:

TOF: tetralogy of Fallot, CHD: congenital cardiac defects.: HLH: hypoplastic left heart syndrome, ASD: atrial septal defect, ATSL arterial tortuosity syndrome, VSD: ventricular septal defect, PDA: patent ductus arteriousus,TAPVD: total anomalous pulmonary venous defect,DORV: double outlet right ventricle,TGA: transposition of great artery,DILV: double inlet left ventricle,COA: cortication of aorta F: female, M: Male, XLR: X-linked recessive, CH: compound heterozygous , AD: Autosomal dominant, AR: autosomal recessive,US: uncertain significance, UPD: uniparental disomy.

-ACMG/AMP sub-classification:

PM1: Located in a mutational hot spot and/or critical and well-established functional domain (e.g. active site of an enzyme) without benign variation

PM2: Absent from controls (or at extremely low frequency if recessive in Exome Sequencing Project, 1000 Genomes or ExAC

PM5: Novel missense change at an amino acid residue where a different missense change determined to be pathogenic has been seen before

BS1: Allele frequency is greater than expected for disorder

BP1: Missense variant in a gene for which primarily truncating variants are known to cause disease

BP4: Multiple lines of computational evidence suggest no impact on gene or gene product (conservation, evolutionary, splicing impact, etc)

BP6: Reputable source recently reports variant as benign but the evidence is not available to the laboratory to perform an independent evaluation

PVS1: Null variant (nonsense, frameshift, canonical +/−1 or 2 splice sites, initiation codon, single or multi-exon deletion) in a gene where loss of function (LOF)
is a known mechanism of disease

PS2: De novo (both maternity and paternity confirmed) in a patient with the disease and no family history

PP2: Missense variant in a gene that has a low rate of benign missense variation and where missense variants are a common mechanism of disease

PP3: Multiple lines of computational evidence support a deleterious effect on the gene or gene product (conservation, evolutionary, splicing impact, etc)

PP5: Reputable source recently reports variant as pathogenic but the evidence is not available to the laboratory to perform an independent evaluation

*: number of enrolled family member is not enough to identify the source of the variant.

**: father was not enrolled, could not identify the source of the other variant copy.

***: Mother was not enrolled, could not identify the source of the other variant copy.

References

1.	Savarese, Marco, Lorenzo Maggi, Anna Vihola, Per Harald Jonson, Giorgio Tasca, Lucia Ruggiero, Luca Bello, Francesca Magri, Teresa Giugliano, and Annalaura Torella. "Interpreting Genetic Variants in Titin in Patients with Muscle Disorders." *JAMA Neurol.* 75, no. 5 (2018): 557-65.

2.	Faiyaz‐Ul‐Haque, M, SHE Zaidi, AA Wahab, A Eltohami, MS Al‐Mureikhi, G Al‐Thani, VD Peltekova, L‐C Tsui, and AS Teebi. "Identification of a P. Ser81arg Encoding Mutation in Slc2a10 Gene of Arterial Tortuosity Syndrome Patients from 10 Qatari Families." *Clin. Genet.* 74, no. 2 (2008): 189-93.

3.	Faiyaz-Ul-Haque, Muhammad, Syed HE Zaidi, Nouriyah Al-Sanna, Abdulrahman Alswaid, Tariq Momenah, Namik Kaya, Fouad Al-Dayel, Issam Bouhoaigah, Mohammed Saliem, and Lap-Chee Tsui. "A Novel Missense and a Recurrent Mutation in Slc2a10 Gene of Patients Affected with Arterial Tortuosity Syndrome." *Atherosclerosis* 203, no. 2 (2009): 466-71.

4.	[VCV000773847.2], National Center for Biotechnology Information. <https://www.ncbi.nlm.nih.gov/clinvar/variation/VCV000773847.2> (

5.	National Center for Biotechnology Information. . "[Vcv000167010.9]." ClinVar, <https://www.ncbi.nlm.nih.gov/clinvar/variation/VCV000167010.9> (

6.	[VCV000722668.2], National Center for Biotechnology Information. ClinVar, <https://www.ncbi.nlm.nih.gov/clinvar/variation/VCV000722668.2> (

7.	Information, National Center for Biotechnology. "Clinvar; [Vcv000722667.2]." <https://www.ncbi.nlm.nih.gov/clinvar/variation/VCV000722667.2> (

8.	Zhu, Tianwen, Xiaohui Gong, Fei Bei, Li Ma, Jingjing Sun, Jian Wang, Gang Qiu, Jianhua Sun, Yu Sun, and Yongjun Zhang. "Primary Immunodeficiency‐Related Genes in Neonatal Intensive Care Unit Patients with Various Genetic Immune Abnormalities: A Multicentre Study in China." *Clin. Transl. Immunol* 10, no. 3 (2021): e1266.

9.	Sun, Yifan, Jingjing Sun, Na Li, Cheng Cai, Xiaohui Gong, and Li Ma. "Phenotypic Spectrum of Typical Charge Syndrome in a Chinese Male Neonate: A Case Report." *Transl. Pediatr.* 9, no. 2 (2020): 180.

10.	National Center for Biotechnology Information, [VCV000136948.9]. "Clinvar." <https://www.ncbi.nlm.nih.gov/clinvar/variation/VCV000136948.9> (

11.	[VCV000707217.7], National Center for Biotechnology Information. <https://www.ncbi.nlm.nih.gov/clinvar/variation/VCV000707217.7> (

12.	[VCV000129948.9], National Center for Biotechnology Information. "National Center for Biotechnology Information. [Vcv000129948.9]." ClinVar, <https://www.ncbi.nlm.nih.gov/clinvar/variation/VCV000129948.9> (

13.	Kanthi, Anil, Malavika Hebbar, Stephanie L Bielas, Katta M Girisha, and Anju Shukla. "Bi-Allelic C. 181_183deltgt in Btb Domain of Klhl7 Is Associated with Overlapping Phenotypes of Crisponi/Ciss1-Like and Bohring-Opitz Like Syndrome." *Eur. J. Med. Genet.* 62, no. 6 (2019): 103528.

14.	[VCV000216489.13], National Center for Biotechnology Information. <https://www.ncbi.nlm.nih.gov/clinvar/variation/VCV000216489.13> (

15.	National Center for Biotechnology Information, [VCV000258032.9]. "National Center for Biotechnology Information,[Vcv000258032.9]." <https://www.ncbi.nlm.nih.gov/clinvar/variation/VCV000258032.9> (

16.	[VCV000010738.32], National Center for Biotechnology Information. "National Center for Biotechnology Information. [Vcv000010738.32]." ClinVar, <https://www.ncbi.nlm.nih.gov/clinvar/variation/VCV000010738.32> (
